# Tumor vascular status controls oxygen delivery facilitated by infused polymerized hemoglobins with varying oxygen affinity

**DOI:** 10.1371/journal.pcbi.1008157

**Published:** 2020-08-20

**Authors:** Donald A. Belcher, Alfredo Lucas, Pedro Cabrales, Andre F. Palmer

**Affiliations:** 1 William G. Lowrie Department of Chemical and Biomolecular Engineering, The Ohio State University, Columbus, Ohio, United States of America; 2 Department of Bioengineering, University of California, San Diego, La Jolla, California, United States of America; University of Michigan, UNITED STATES

## Abstract

Oxygen (O_2_) delivery facilitated by hemoglobin (Hb)-based O_2_ carriers (HBOCs) is a promising strategy to increase the effectiveness of chemotherapeutics for treatment of solid tumors. However, the heterogeneous vascular structures present within tumors complicates evaluating the oxygenation potential of HBOCs within the tumor microenvironment. To account for spatial variations in the vasculature and tumor tissue that occur during tumor growth, we used a computational model to develop artificial tumor constructs. With these simulated tumors, we performed a polymerized human hemoglobin (hHb) (PolyhHb) enhanced oxygenation simulation accounting for differences in the physiologic characteristics of human and mouse blood. The results from this model were used to determine the potential effectiveness of different treatment options including a top load (low volume) and exchange (large volume) infusion of a tense quaternary state (T-State) PolyhHb, relaxed quaternary state (R-State) PolyhHb, and a non O_2_ carrying control. Principal component analysis (PCA) revealed correlations between the different regimes of effectiveness within the different simulated dosage options. In general, we found that infusion of T-State PolyhHb is more likely to decrease tissue hypoxia and modulate the metabolic rate of O_2_ consumption. Though the developed models are not a definitive descriptor of O_2_ carrier interaction in tumor capillary networks, we accounted for factors such as non-uniform vascular density and permeability that limit the applicability of O_2_ carriers during infusion. Finally, we have used these validated computational models to establish potential benchmarks to guide tumor treatment during translation of PolyhHb mediated therapies into clinical applications.

## Author summary

High rates of oxygen consumption and abnormal vascularization lead to low oxygen levels within solid tumors. The lack of oxygen results in resistance to chemotherapies and increased rates of cancer progression. Hemoglobin-based oxygen carriers have the potential to increase the amount of oxygen delivered to tumors, which may make chemotherapies more effective. Unfortunately, translating experimental results from mice to humans is complicated by allometric scaling between mice and humans. To predict how these therapies may perform differently between human and murine systems, we computationally predicted how hemoglobin-based oxygen delivery varies between the two organisms. Our model accounts for how variations in the tumor vascular network impact the performance of hemoglobin-based oxygen carriers. This model also allows us to assess how the oxygen affinity of hemoglobin-based oxygen carriers affects the oxygenation of hypoxic tissue. The results of these models help us predict how results from murine models may translate to humans. Also, our models help to highlight what clinically-measurable tumor properties should be measured to predict the effectiveness of hemoglobin-based oxygen carriers in biological systems.

## Introduction

**Fig 1:**
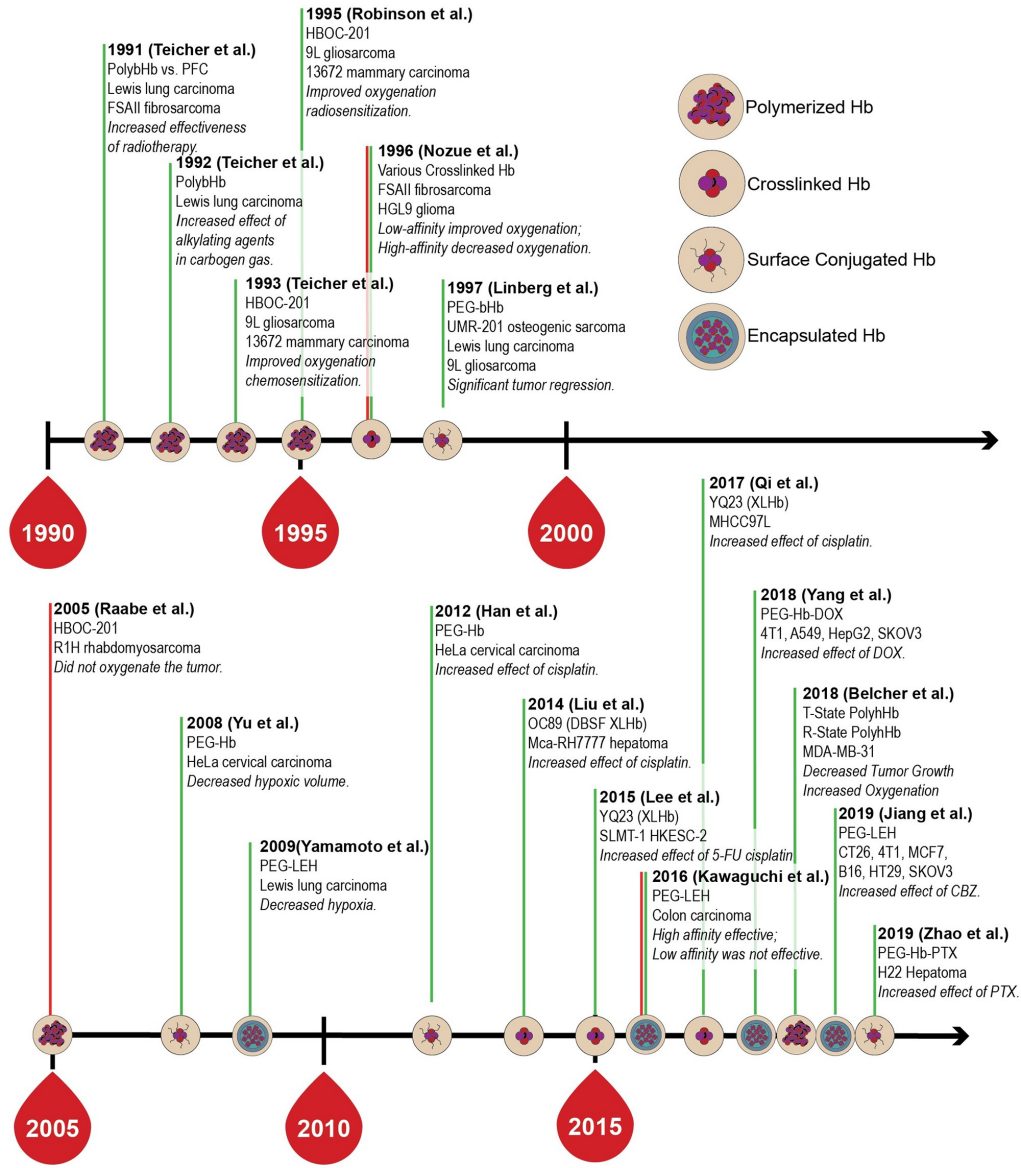
Time-line for the assessment of various HBOCs in the treatment of solid tumors. Green lines indicate positive results, red lines indicate negative results.

The use of hemoglobin (Hb)-based oxygen (O_2_) carriers (HBOCs) as a cancer chemosensitizing agent has been studied for a variety of prior generation HBOCs including crosslinked Hb (XLHb) [1–4],polymerized Hb (PolyHb) [5–12], surface conjugated Hb [13–16], and liposome encapsulated Hb [17–20]. In general, these studies have shown that HBOCs are effective at increasing O_2_ delivery to tumor tissue. However, recent studies have demonstrated that HBOCs may not be efficacious for all tumor types or at specific dosage levels [21]. A time-line for the assessment of HBOC performance in oxygenating solid tissues is shown in Fig 1.

Despite three decades of work, no HBOC has translated into clinical treatment of tumor hypoxia. The stagnated development of HBOCs for use as a chemosensitizing agent is a result of both (1) inherent toxicity of previous generations of HBOCs and the (2) inconsistencies in hypoxia reduction in solid tumors. The toxicity of previous generations of HBOCs is associated with elevated renal toxicity and hypertension resulting from abundant stroma-free low molecular weight Hbs (< 250 kDa) present within previous generations of HBOCs [22]. The presence of these low molecular weight species resulted in clinical failures during implementation of early generation HBOCs [23] Recently, HBOCs with higher molecular weights (> 250 kDa) that are safer to infuse have been developed [12, 24, 25].

The ultimate goal of HBOC modulated oxygenation to tumors is to increase the effectiveness of O_2_-dependent therapies such as chemotherapy. However, there are still concerns over inconsistent hypoxia reduction after HBOC infusion [21]. This inconsistency in O_2_ transfer is likely a result of variations in vascularization and blood flow within tumors. Thus, quantifying how HBOC modulated oxygenation varies with clinically measurable properties of tumor vascularization may help guide development of clinical benchmarks of HBOC co-administration.

Unfortunately, determining these features with animal models alone necessitates the implementation of complex experimental methodology. For example, intravital microscopy techniques are able to examine transient changes within the tumor vascular structure. However, these models are typically geometrically limited to approximately two-dimensional tumor growth within chamber window models. Larger three-dimensional tumor growth relevant to human tissue is instead obtained by implanting tumors within host tissue and waiting for growth. Transiently measuring complete O_2_ dynamics within the tumor structure might be performed with needle electrodes, positron emission tomography [26], and magnetic resonance imaging [27]. Another potential method to assess O_2_ transport is optical mammography, an absorbance-based technique that determines the concentrations of Hb, oxygenated Hb (oxyHb), and deoxygenated Hb (deoxyHb) in breast tissue [28, 29]. Unfortunately, the spatial resolution of these methods are inadequate to resolve arterioles, venules, and capillaries. Instead these methods only allow us to estimate average O_2_ and Hb concentrations within the bulk of tumor tissue.

**Fig 2:**
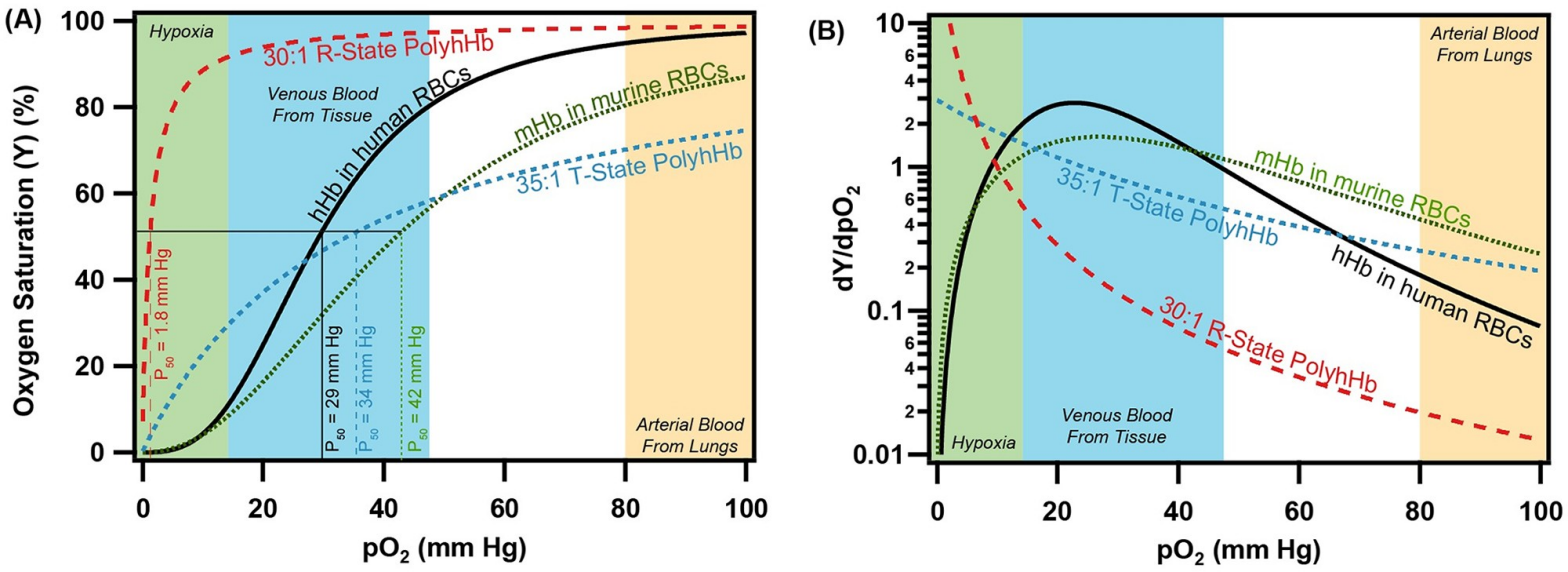
OEC and O_2_ offloading plot for various O_2_ carrying species used in the simulations. (A) The OEC is shown for human hemoglobin (hHb) in human RBCs, mouse hemoglobin (mHb) in mouse RBCs, 30:1 R-State PolyhHb, and 35:1 T-State PolyhHb. (B) The O_2_ offloading plot as a function of the pO_2_ is shown for the same species. Approximate pO_2_ regions for arterial and venous blood under normoxic conditions have been included on this graph for reference.

Furthermore, commonly used small animal models, such as mice, have distinct physiologic differences in O_2_ transport when compared to humans. These differences can be summarized by three alterations in fluid and mass transport when compared to humans: (1) decreased mouse red blood cell (RBC) size [30], (2) increased metabolic rate of O_2_ consumption in the mouse host tissue [31–33], and (3) decreased O_2_ affinity of mouse Hb in RBCs [34]. In mice, the decreased O_2_ affinity of Hb in RBCs may result in reduced O_2_ release from HBOC species in circulation when compared to HBOC performance in humans. An example of these changes in the O_2_ equilibrium curvess (OECs) and the O_2_ offloading plot as a function of partial pressure of dissolved O_2_ (pO_2_) is shown in Fig 2. The O_2_ offloading plot demonstrates that both low O_2_ affinity tense quaternary state (T-State) and high O_2_ affinity relaxed quaternary state (R-State) polymerized human hemoglobin (hHb) (PolyhHb) may have lower rates of O_2_ offloading compared to mouse RBCs under normoxic conditions. The O_2_ offloading of both PolyhHb species only surpass O_2_ offloading of mouse Hb in RBCs under hypoxic conditions (< 15 mm Hg). Though this increase in hypoxic O_2_ offloading should be maintained under hypoxic conditions in humans, low O_2_ affinity T-State PolyhHb should facilitate increased O_2_ offloading in the arteries and arterioles of human subjects [12]. This fundamental difference in HBOC O_2_ delivery potential may impact how results are translated from pre-clinical mouse models of HBOC infusion to clinical applications of HBOC infusion for cancer treatment. Thus understanding how the O_2_ affinity of HBOCs influence O_2_ transfer in humans is vital to understand how tumor oxygenation status may change if HBOCs are applied clinically.

From experimental studies of HBOC tumor treatment, we know that infusion of PolyHb results in a tumor growth delay [5–12]. This effect is thought to occur due to modulation of O_2_ delivery at the host-tumor tissue interface. Unfortunately, there is an absence of clinically available data of these interfacial regions with adequate capillary resolution. Hence, computationally evaluating HBOC facilitated O_2_ mass transport in a connected host tissue-tumor microenvironment is an exciting method to address how changes in the vasculature and host organism impact O_2_ delivery modulation. However, any computational model we develop must be able to translate the physical behavior of the O_2_ carriers to host and tumor properties that are clinically measurable with current low-resolution imaging techniques. We hypothesize that parameters such as the concentration of oxyHb, regional blood volume (*RBV*), and regional blood flow (*RBF*) may predict how HBOCs modulate O_2_ delivery to the tumor. Thus, this study aims to investigate how clinically measurable benchmarks might be useful to determine the oxygenation potential of T-State and R-State PolyhHb. To aid with predicting the effectiveness of PolyhHb within the heterogeneous tumor mass, we have redesigned an existing multi-scale 3D computational model of solid tumor growth and oxygenation to rapidly screen *in silico* the library of PolyhHbs in multiple simulated breast cancer tumors prior to *in vivo* analysis [35–39]. This newly developed model incorporates non-linear O_2_ transport from both Hb in RBCs and HBOC in plasma. With this *in-silico* model, we explored how properties of the tumor micro-environment, including tumor growth, tumor density, and tumor location influence the oxygenation performance of various PolyhHbs. This is especially important because vascularization within solid tumors has a significant effect on O_2_ and nutrient transport [40, 41]. Additionally, we can use these models to create physiological representations of both human and mouse tumors. We can then use these tumor models to assess how HBOC delivery in tumors may change when applied clinically.

## Results

**Fig 3:**
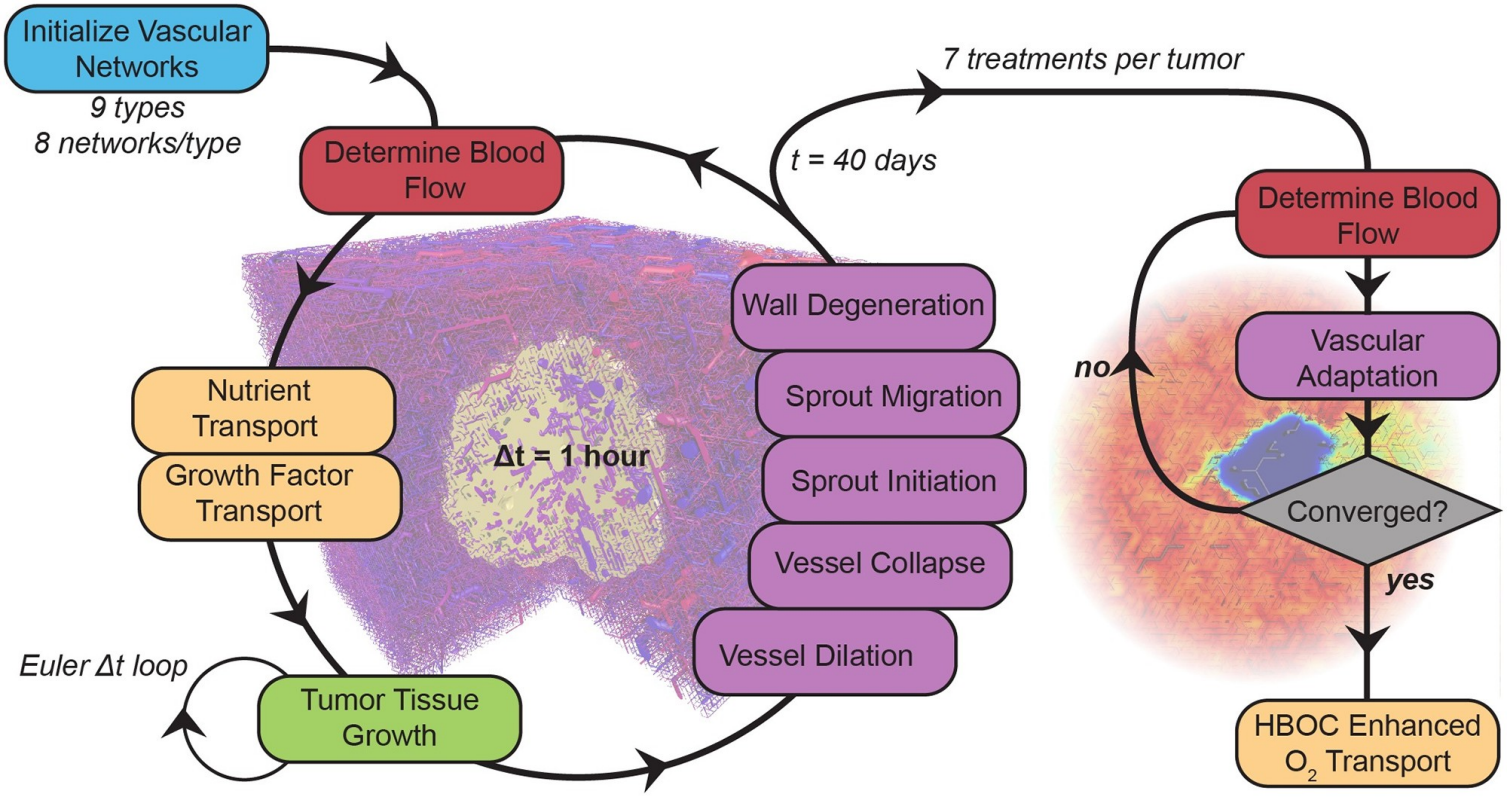
Model flow diagram. At each time step, blood flow, distribution of nutrients/growth factors and vascular remodeling are computed. The tissue phase remodels at shorter time steps within the main loop. The resulting artificial tumors are transferred to the infusion model. Hemodilution and the HBOC enhanced viscosity modulates flow and vascular adaptation until the microvascular system is stable. After this, HBOC enhanced oxygenation is modeled.

To test the oxygenation potential of PolyhHb within tumor microvascular networks, we implemented a modified version of the Tumorcode artificial tumor construct simulation framework [39] that incorporates O_2_ transport from both Hb in RBCs and HBOCs. A flowchart of the simulation is shown in Fig 3.

### Artificial tumor construct growth and properties

For these simulations, we began by generating artificial blood vessel networks with a variety of host tissues using constraint centered optimization on a face-centered cubic lattice. The method we use for generating the three-dimensional vascular structure follows methods previously described [39, 42, 43]. Variations in host structure approximate differences in experimental cohorts. Mouse and human 3D tumor constructs were generated using the continuum model for tumor growth and the simplified vascular adaptation model described in the S1 Appendix. Each tumor was generated within 6 mm wide cubic artificial vascular networks with 9 different artery/vein node configurations (Type A-I) (72 vascular beds). With the generated artificial vessel networks, we simulated 40 days of tumor growth with a multiphase continuum model for tumor expansion. This model of tumor tissue expansion consists of 5 phases: normal tissue, tumor tissue, necrotic tissue, extra-cellular matrix, and interstitial fluid. Both tumor growth and vascular remodeling were coordinated with a system of diffusible species representing nutrients and growth factors (i.e. O_2_ and vascular endothelial growth factor (VEGF)) throughout the tissue space. During tumor growth, we model vascular deterioration and VEGF mediated angiogenesis modulated by changes in vascular fluid flow and VEGF stimulated vascular expansion throughout the vascular network.

**Fig 4:**
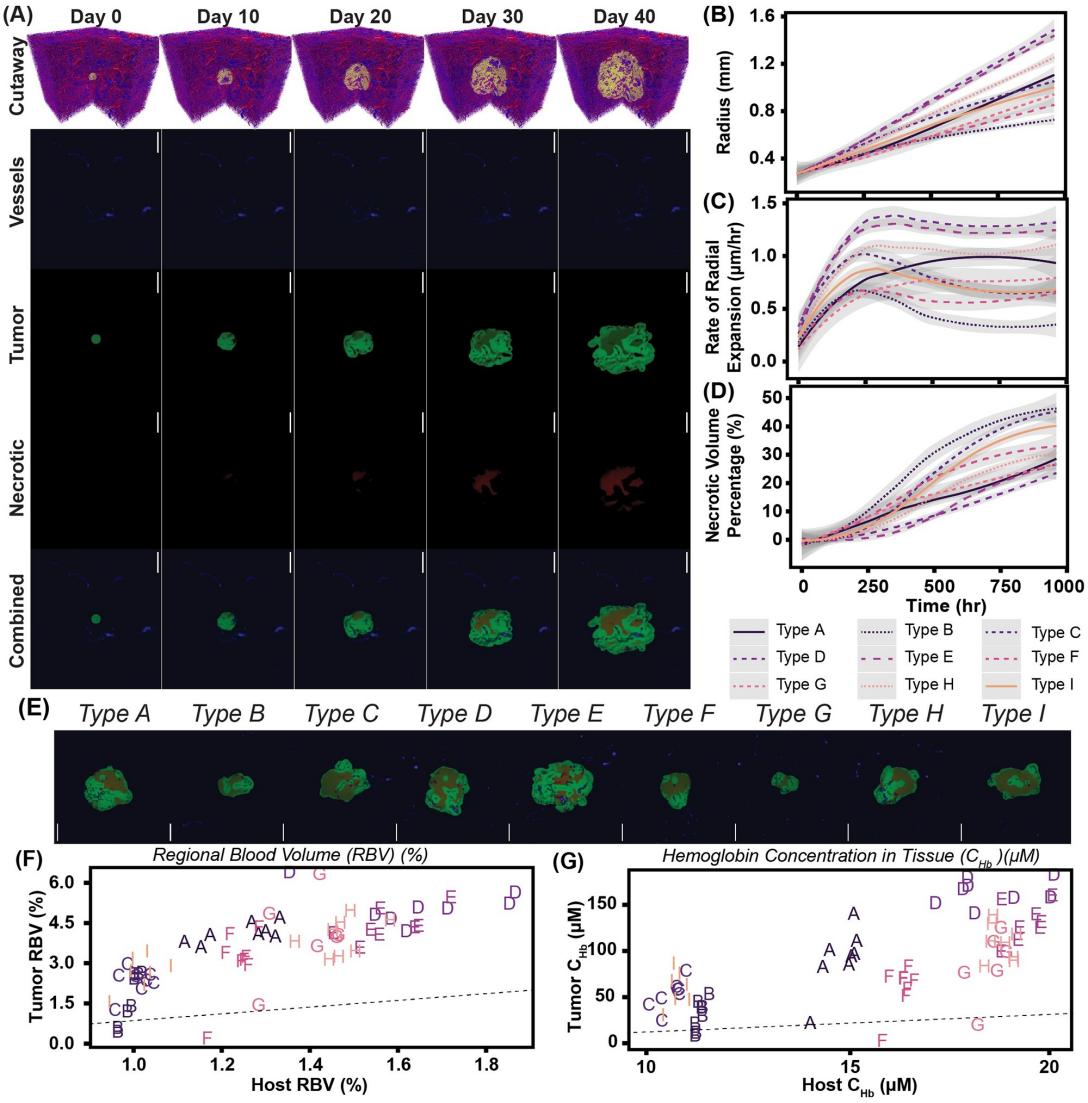
Artificial mouse tumor growth and resulting biophysical properties after 40 days of growth. (A) Visualization of cutaways, vessel (blue), tumor (green) and necrotic (red) volume fraction cross sections for artificial mouse tumor growth over 40 days. The tumor shown here was grown in a type A vascular bed. Also shown in this figure are the (B) radius, (C) rate of radial expansion, and (D) necrotic volume percentages for each vessel bed type (Type A-I). Shaded areas in these plots represent a 95% confidence interval across each type of vessel bed configuration. (E) Visualization of combined volume fraction cross sections for selected tumors from each of the vessel bed types (Type A-I). Comparison of (F) *RBV* and (G) *C*_*Hb*,*tis*_ between the tumor and host tissue in the artificial tumor constructs. The letter labels indicate the vessel configuration. The dashed line separates the tumor properties greater than and less than the host properties. For all tumor cross sections the scale bar is 1 mm.

Cross sections of depicting progression of the vascular network (blue), tumor tissue (green), and necrotic tissue (red) over a 40 day growth period for a tumor grown in a mouse type A vascular bed were used to examine the heterogenity of tumor growth. To quantify how artificial tumors progressed over 40 days of growth, various tumor properties including tumor radius, rate of radial expansion, tumor sphericity, necrotic volume%, *RBV* and Hb concentration in the tissue (*C*_*Hb*,*tis*_) concentration in the tissue (*C*_*Hb*,*tis*_) were recorded every 48 simulated hours during the growth of each artificial tumor construct. During tumor growth there was a correlative relationship in development of tumor *RBV* and tumor *C*_*Hb*,*tis*_ during tumor expansion. The rate of radial expansion for Type D, E, and H tumors was higher than other vessel configurations. Visual examination of the combined tumor volume percentages (vascular:blue, tumor:green, necrotic:red) at the endpoint (t = 40 days)confirmed that the generated artificial tumors are topologically diverse with significant variations between vessel bed configurations. After visually confirming the heterogeneous composition of the artificial tumor constructs, the effect of tumor growth on remodeling of the vasculature was assessed with volume averaged properties. In general, tumor growth led to an increase in microvascular density (*MVD*), *RBV*, vascular surface density, and *C*_*Hb*,*tis*_. A summary of these results is shown in Fig 4. Additional information on the cohort of artificially generated tumors can be found in the S2 Appendix.

### Polymerized hemoglobin enhanced oxygenation

With the resulting artificially constructed tumor constructs, we simulated a PolyhHb mediated infusion with vascular adaptation and PolyhHb enhanced oxygenation. Here we simulated 7 infusion conditions for each artificially generated tumor construct. We simulated two dosing levels: a large volume exchange infusion and a low volume top-load infusion. To better examine performance of the materials, we analyzed a non-O_2_ carrying control, a 35:1 T-State PolyhHb, and a 30:1 R-State PolyhHb. An unsupplemented baseline condition was also simulated to compare how the various infusions modulate O_2_ delivery. To confirm if simulations were accurately modeling the O_2_ distribution within the tumor we validated the simulation with intravital vascular pO_2_ and Hb/PolyhHb saturation gathered from a mouse chamber window model as described in the S6 Appendix.

### Principal component analysis

**Fig 5:**
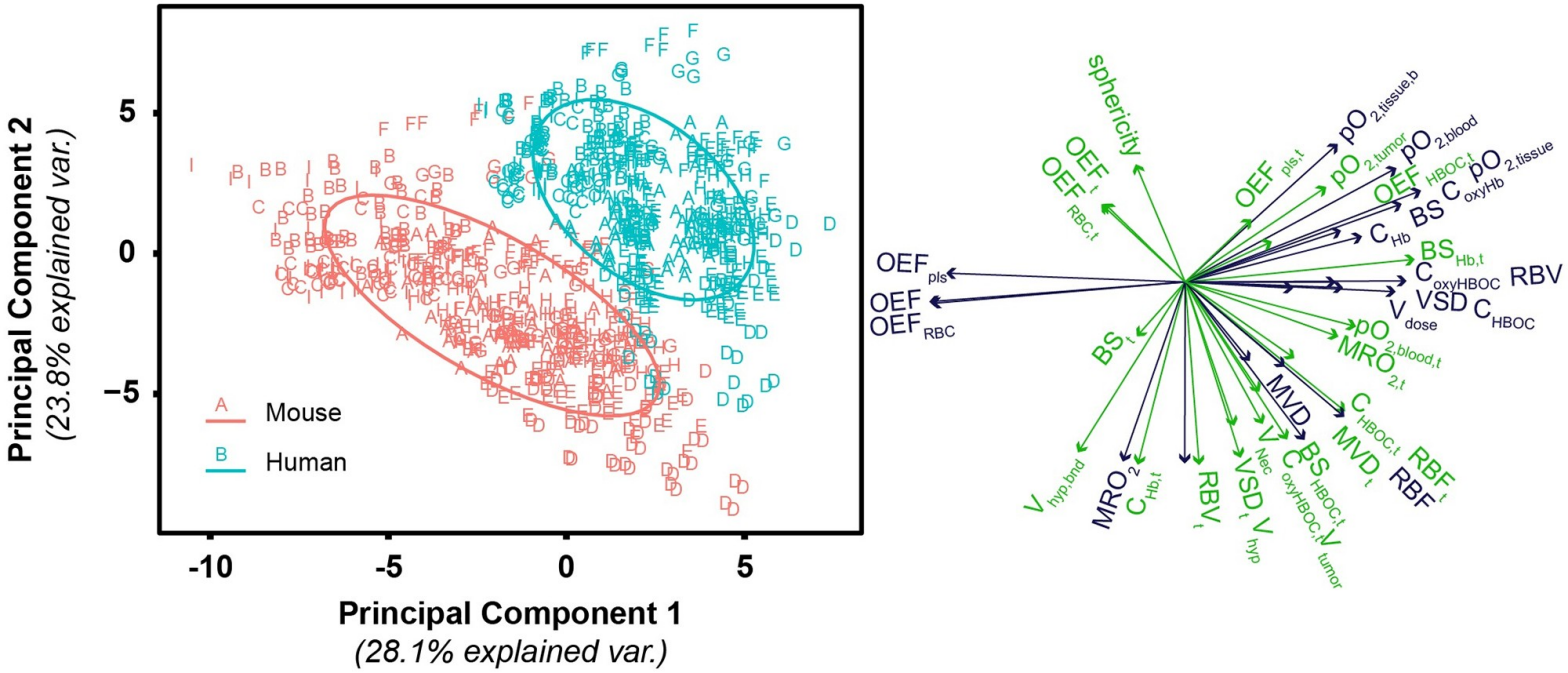
PCA biplot for principal components 1 and 2 of the mouse and human tumor model. In this figure, groupings are organized by organism type (human, mouse). Left: PC scores for tissue and O_2_ delivery for each of the simulated tumor treatments. Ellipses are drawn around each group with 68% of the normal probability. Right: Loading plots relating how each parameter influences the corresponding principal component. Green vectors indicate tumor properties while blue vectors indicate host tissue properties. Labels indicate the corresponding tumor property.

To better predict correlation and grouping of spatially averaged and clinically measurable parameters in the simulation, principal component analysis (PCA) was performed on the entire dataset of baseline and post-infusion tumors. Differences between mouse and human O_2_ transport simulations are shown in Fig 5. The properties that separate the mouse and human tumor groups (tumor O_2_ extraction fraction from HBOCs (*OEF*_*HBOC*_, tumor tissue pO_2_, and tumor blood saturation) are primarily linked to O_2_ transport in the tumor. In comparison, properties that are relatively unchanged between the human and mouse group are overall O_2_ extraction fraction (*OEF*), *MVD*, and *RBF*. There is significantly greater variance in artificially generated human tumors compared to artificially generated mouse tumors. Because of these differences, the data-set for the following analysis of tumor treatment are split between the two host organisms.

**Fig 6:**
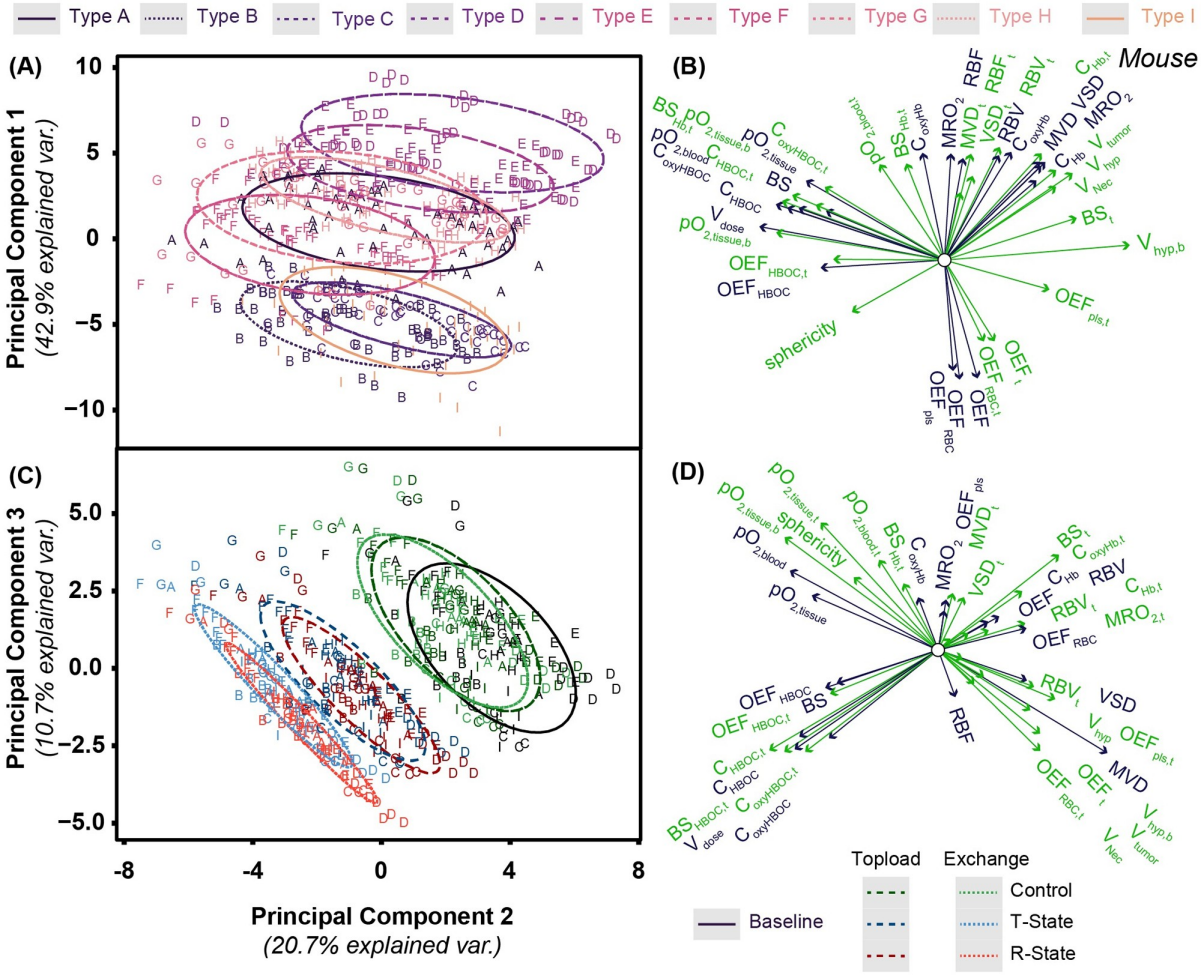
PCA biplot for principal components 1 and 2 for the analysis of the mouse model. PC scores for tissue properties and O_2_ delivery for each of the simulated mouse tumor treatments for (A) principal component 1 and (C) principal component 3 as a function of principal component 2. Grouping in panel A is based on tumor vascular bed type. Grouping in panel B is based on dosing type. Ellipses are drawn around each group with 68% of the normal probability. Loading plots relating how each parameter influences the corresponding principal component is shown for (B) principal component 1 and (D) principal component 3 as a function of principal component 2. Loading vectors with magnitude less than 0.5 have been excluded from this plot. Green vectors indicate tumor properties while blue vectors indicate host tissue properties. Labels on each vector indicate the corresponding tumor property.

In mouse tumors, architectural and bulk properties of the tumor account for approximately 63% of the explained variance. Changes based on treatment type account for around 31% of the explained variance. A biplot for principal component 1 (PC1), principal component 2 (PC2), and principal component 3 (PC3) which shows these effects can be found in Fig 6. For PC1 and PC2,vascular bed type is used to group the tumors. In general, the different types of tumors are differentiated by *OEF*, O_2_ extraction fraction from plasma (*OEF*_*plas*_), O_2_ extraction fraction from Hb in RBCs *OEF*_*Hb*_, *RBV*, *RBF*, *MVD*, tumor volume, and necrotic volume percentage. In different vascular beds, there was differentiation based on O_2_ properties, including hypoxic volume percentage and pO_2_.

PC3 and PC2 account for approximately 31% of the variance in the simulated mouse tumors. Non-O_2_ carrying control groups overlap with the baseline group. All PolyhHb infusion groups are separate from baseline and control groups. However, there is no significant difference between T-State and R-State PolyhHb at similar infusion dose volumes. Separation between these groups is dependent on (*OEF*_*HBOC*_, metabolic rate of O_2_ consumption (*MRO*_2_), and tumor tissue Hb saturation. Within these groups, the increasing dosages correlate with an increase in PolyhHb saturation and blood saturation. There is also a decrease in *C*_*Hb*,*tis*_ with increasing dose volume.

**Fig 7:**
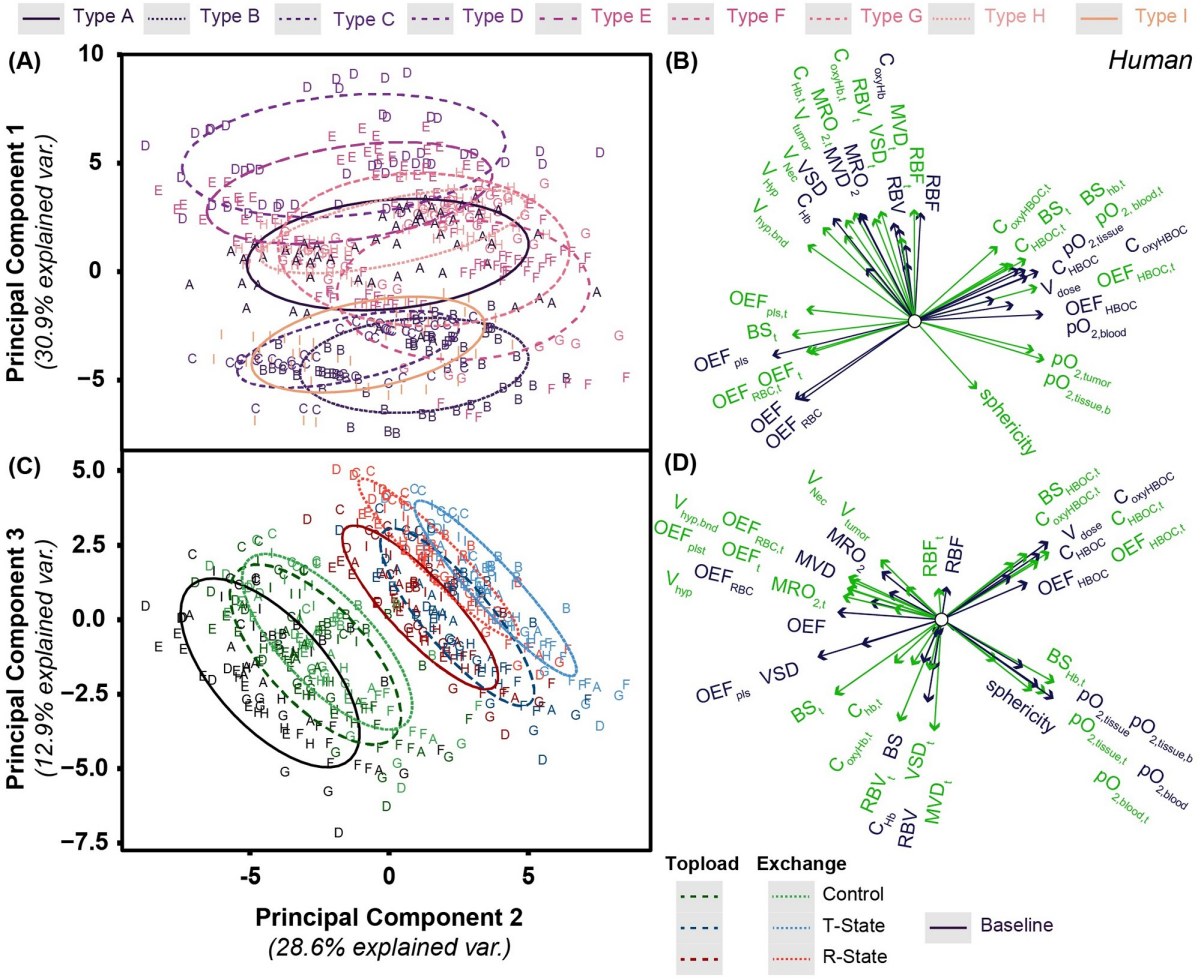
PCA biplot for principal components 1 and 2 for the analysis of the human model. PC scores for tissue properties and O_2_ delivery for each of the simulated mouse tumor treatments for (A) principal component 1 and (C) principal component 3 as a function of principal component 2. Grouping in panel A is based on tumor vascular bed type. Grouping in panel B is based on dosing type. Ellipses are drawn around each group with 68% of the normal probability. Loading plots relating how each parameter influences the corresponding principal component is shown for (B) principal component 1 and (D) principal component 3 as a function of principal component 2. Loading vectors with magnitude less than 0.5 have been excluded from this plot. Green vectors indicate tumor properties while blue vectors indicate host tissue properties. Labels on each vector indicate the corresponding tumor property.

In addition to PCA performed on mouse tumor data, PCA was performed on data from simulated human tumor constructs. A biplot of this analysis is shown in Fig 7. Compared to mouse data, there were similar trends observed in grouping and loading vectors of PC1 and PC2 depending on vascular bed configuration. For human tumors, there is significantly tighter grouping in loading vectors associated with vascular fluid flow and tissue oxygenation.

### Comparison of simulated results

**Fig 8:**
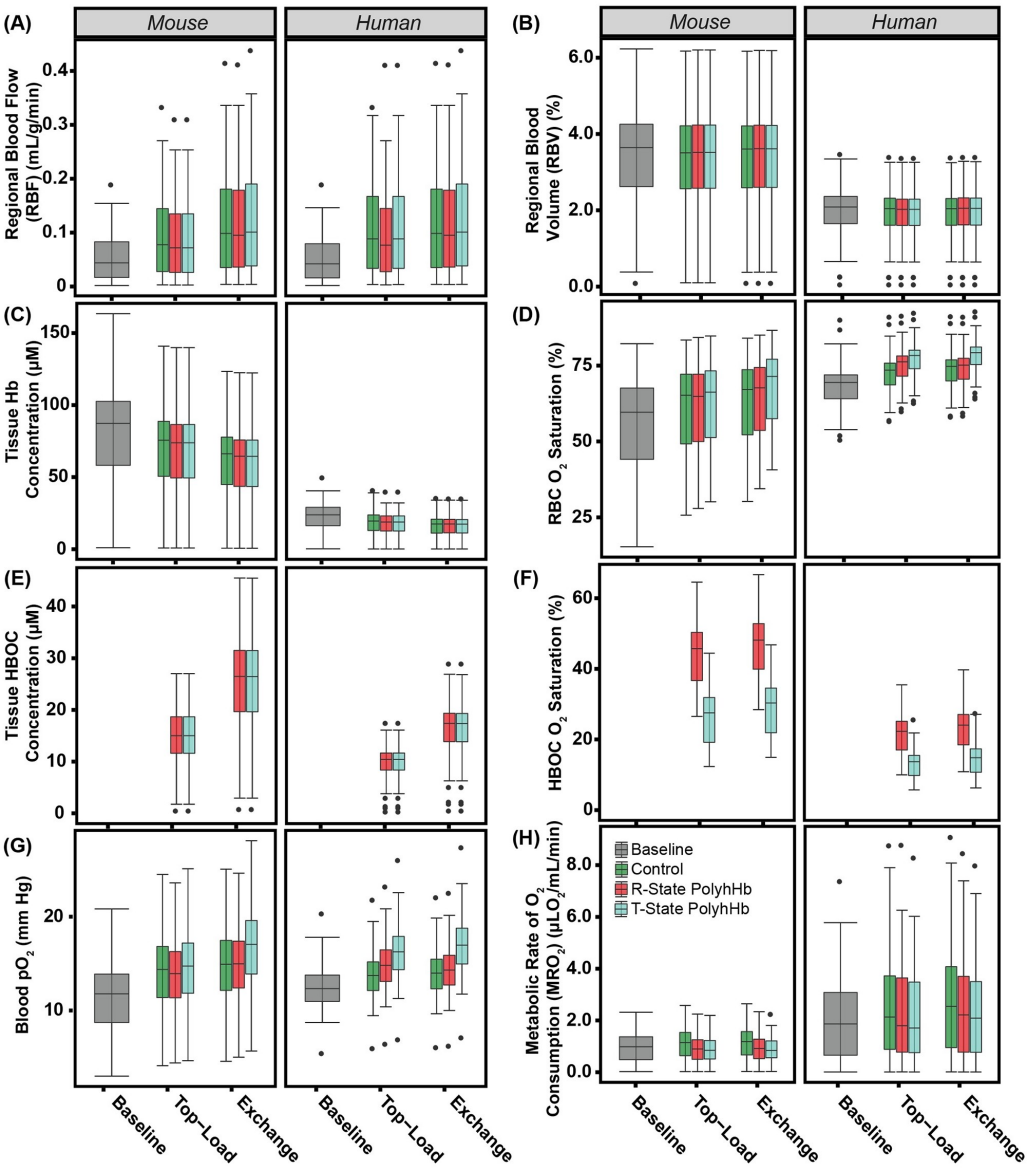
Changes in tumor vascular architecture and Hb/PolyhHb concentrations for the simulated PolyhHb enhanced infusion model. Variations in the (A) *RBV*, (B) *RBF*, (C) *C*_*Hb*,*tis*_, (D) Hb O_2_ saturation in RBC, (E) PolyhHb (HBOC) concentration, (F) PolyhHb (HBOC) O_2_ saturation, (G) blood pO_2_, and (H) *MRO*_2_ for the baseline, top-load, and exchange infusion of the control, 30:1 R-State PolyhHb, and 35:1 T-State PolyhHb.

Changes in the vascular architecture of artificial tumors and resulting changes in Hb/PolyhHb perfused tumor constructs after simulated infusions are shown in Fig 8. Both top-load and exchange infusion models result in significant (p<0.05) increases in *RBF*. Additionally, exchange infusion significantly (p<0.05) increases *RBF* compared to top-load infusion. After top-load infusion, there is no significant change in *RBF* between different simulated treatment types. However, after exchange infusion, there is a significant difference between all simulated treatment types and the control. The 30:1 R-State PolyhHb treatment has the least improvement in *RBF* compared to other exchange infusion treatment options.

As expected from simulated hemodilution, all treatment types significantly (p<0.05) decreased *C*_*Hb*,*tis*_. However, there are no significant differences between the control and PolyhHb treatments at the same dosage volume. Despite observing a decrease in *C*_*Hb*,*tis*_, there is also a significant (p<0.05) increase in O_2_ saturation of Hb in RBCs for all treatments compared to the baseline. During 35:1 T-State PolyhHb infusion, Hb saturation was significantly (p<0.05) greater than all other treatment options in both human and mouse models. Even though dose volume has a significant (p<0.05) effect on PolyhHb concentration in the tumor, there is negligible difference between the concentration of T-State and R-State PolyhHb at the same dose volume. As expected from the reduced *RBV* in the human model, the corresponding PolyhHb concentration is lower in human models compared to mouse models at corresponding dose volumes. Due to the low O_2_ affinity of T-State PolyhHb, the concentration of O_2_ saturated T-State PolyhHb is significantly(p<0.05) less than the concentration of O_2_ saturated R-State PolyhHb.

Additionally, vascular pO_2_ within the artificial tumor significantly (p<0.05) increased in both top-load and exchange infusion models compared to the baseline. In mouse top-load dose simulations, there was no significant changes in vascular pO_2_ between T-State PolyhHb, R-State PolyhHb, and the control. However, in the exchange infusion model, there is a significant (p<0.05) increase in T-State PolyhHb infusion compared to both R-State PolyhHb and control solutions.

**Fig 9:**
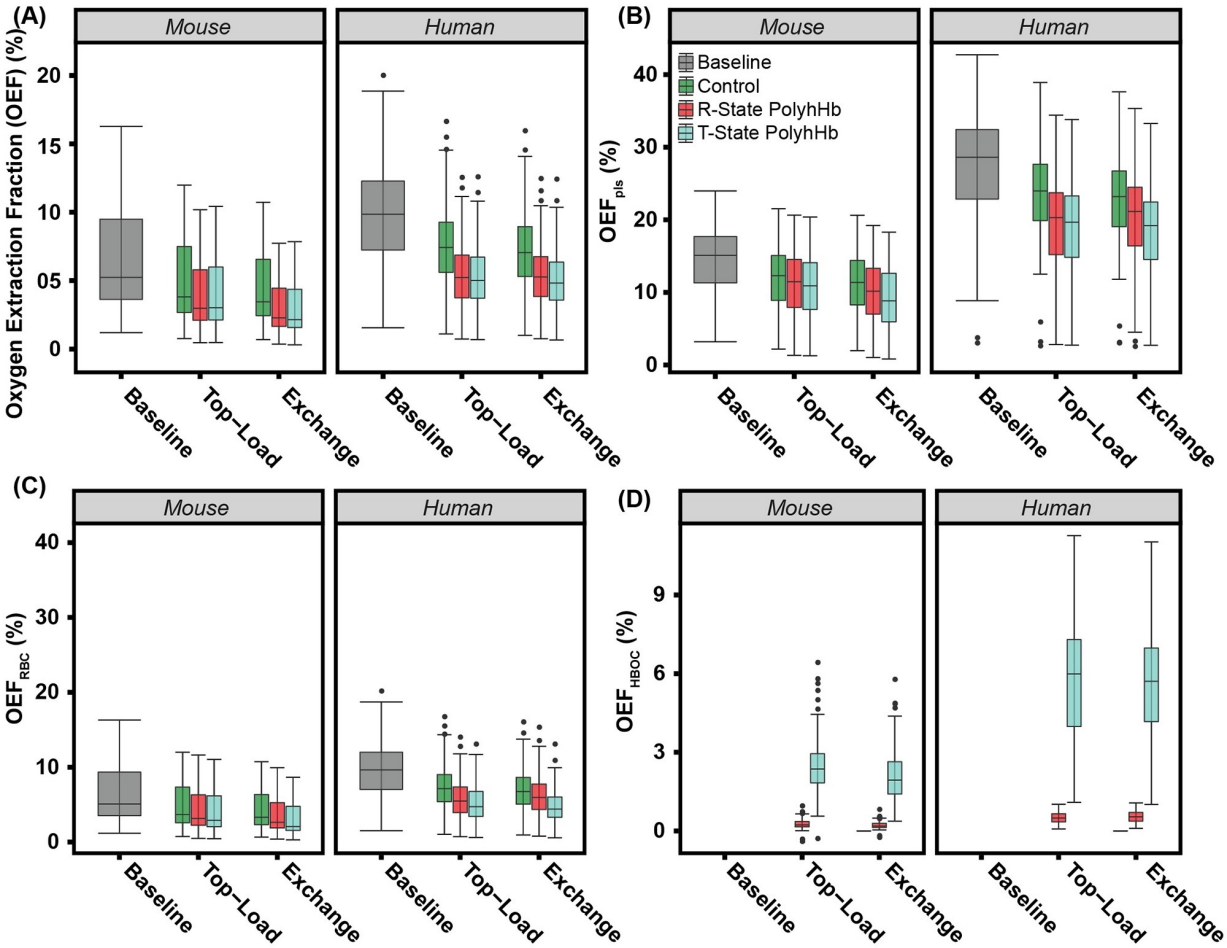
Changes in *OEF* for the simulated PolyhHb enhanced infusion model. Variations in (A) *OEF*, (B) Blood pO_2_, (C) *OEF*_*plas*_, (D) *OEF*_*Hb*_ O_2_ saturation in RBC, and (E) *OEF*_*HBOC*_ (HBOC) concentration infusion of the control, 30:1 R-State PolyhHb, and 35:1 T-State PolyhHb.

The resulting changes in the *OEF*, *OEF*_*plas*_, *OEF*_*Hb*_, and *OEF*_*HBOC*_ are shown in Fig 9. Both top-load and exchange infusion models led to significant (p<0.05) decreases in *OEF* in both mouse and human models. Despite the drastic difference in *MRO*_2_, there is relatively little difference in *OEF* between mouse and human models. Infusion of 30:1 R-State PolyhHb and 35:1 T-State PolyhHb significantly (p<0.05) decreased *OEF* compared to the non O_2_ carrying control infusions. There is no significant difference in total *OEF* between T-State and R-State PolyhHb. There are similar dose-dependent decreases in *OEF*_*plas*_ and *OEF*_*Hb*_. However, T-State PolyhHb led to a significant (p<0.05) decrease in *OEF*_*plas*_ and the *OEF*_*Hb*_ compared to R-State PolyhHb. This decrease is offset by a significant (p<0.05) increase in T-State PolyhHb *OEF*_*HBOC*_ compared to R-State PolyhHb.

**Fig 10:**
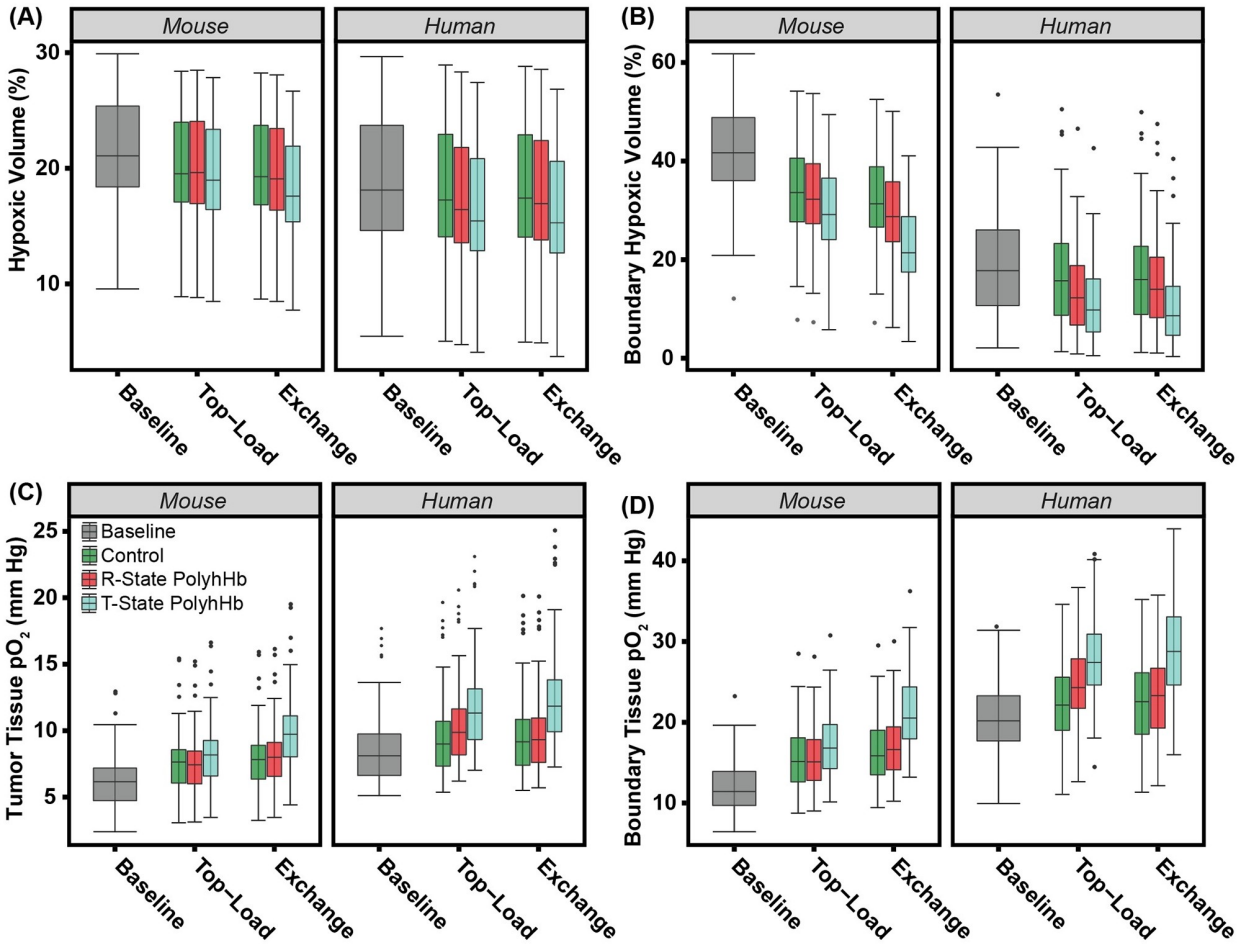
Changes in tumor hypoxia for the simulated PolyhHb enhanced infusion model. Variations in the (A) tumor tissue pO_2_, (B) boundary tissue pO_2_, (C) hypoxic fraction, and (D) boundary hypoxic fraction for infusion of the control, 30:1 R-State PolyhHb, and 35:1 T-State PolyhHb.

We calculated hypoxic volume fractions by taking the volume fraction of normal and tumor cells that are below a hypoxic threshold (pO_2_ < 5 mm Hg). To match with literature approximations, we did not consider necrotic cells as part of the hypoxic volume. This is because necrotic cells are dead and thus do not have active hypoxia-inducible factors [44]. Changes in pO_2_ and hypoxic volume fractions can be found in Fig 10. Tumor tissue pO_2_ was significantly (p<0.05) higher in human tissue compared to mouse tissue. In both top-load and exchange infusions, there is a significant (p<0.05) increase in tumor tissue pO_2_ and significant (p<0.05) decrease in hypoxic volume fraction for both control and PolyHb infusions. Infusion of 35:1 T-State PolyhHb resulted in a significant (p<0.05) increase in tumor tissue pO_2_ compared to infusion of 30:1 R-State PolyhHb and control in both top-load and exchange infusion models. Despite observing this difference, there are no significant changes in hypoxic volume fraction between 35:1 T-State PolyhHb, 30:1 R-State PolyhHb, and control at both dose volumes.

In addition to the overall hypoxic fraction within the tumor for all of the treatment solutions, we also examined hypoxia within the tumor/host boundary region (volume fraction of tumor cells (*ϕ*_*T*_) > 0 and volume fraction of necrotic cells (*ϕ*_*D*_) = 0). In this region, both the top-load and exchange infusion models led to a significant (p<0.05) increase in tissue pO_2_ and decrease in hypoxic volume fraction compared to baseline. In the exchange infusion model, 30:1 R-State PolyhHb infusion resulted in a significant (p<0.05) increase in tissue pO_2_ and the hypoxic volume fraction compared to the control.

### Performance thresholds

For this analysis, we compared the percent variation to the baseline conditions for each of the simulated infusion models. The percent change was calculated compared to the baseline (Δ% = 100 ⋅ (*V* − *V*_*BL*_)/*V*_*BL*_). For each of these systems, the properties of the baseline and post-infusion tumors led to a percent change in the hypoxic volume and *MRO*_2_. For brevity, variables that did not significantly correlate with changes in the tumor oxygenation results were excluded from this analysis.

**Fig 11:**
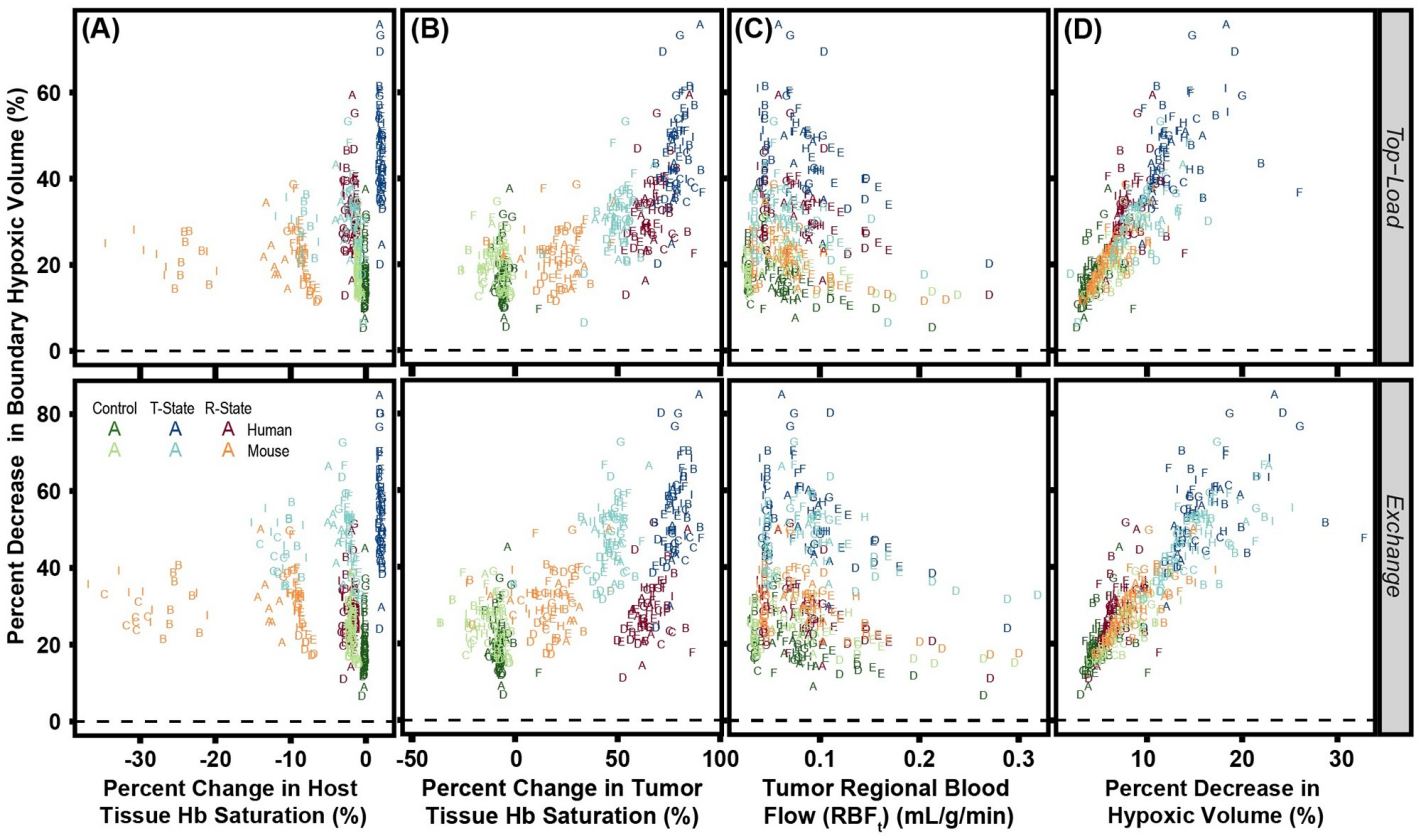
The effect of (A) host tissue Hb saturation, (B) tumor tissue Hb saturation, (C) tumor *RBF* and (D) percent decrease in total tumor hypoxic volume on the percent changes in the boundary hypoxic volume. The baseline (unsupplemented) condition is depicted as the dashed line at zero. Letters labeling each data point indicate the vessel bed configuration for that artificial tumor construct. The non O_2_ carrying control is green, R-State PolyhHb is red, and T-State PolyhHb is blue.

The effects of percent change in host tissue Hb saturation, percent change in tumor tissue Hb saturation, tumor *RBF*, and percent change in hypoxic volume are shown in Fig 11. The changes in boundary hypoxic fractions for 30:1 R-State PolyhHb infusion is clustered closer to the non O_2_ carrying control than to 35:1 T-State PolyhHb for both mouse and human models. Top-load infusion of R-State PolyhHb result in equivalent hypoxic fraction reduction compared to the non-O_2_ carrying control. In top-load infusion models we see relatively little correlation between *RBV* and the percent changes in the hypoxic volume when the *RBV* is above 0.1 mL/g/min. Decreases in the regional blood flow typically leads to greater reductions in the hypoxic fraction. Distinct groups were formed based upon Hb saturation decreases after infusion of R-State PolyhHb, T-State PolyhHb, or the non O_2_ carrying control. Like the total tumor hypoxic volume, decreases in Hb O_2_ saturation resulted in further decreases in the boundary hypoxic fraction for T-State PolyhHb and R-State PolyhHb. For mice, decreases in the percent change of the tissue Hb saturation led to increased boundary hypoxic reduction for T-State PolyhHb but not for R-State PolyhHb. In the human model, PolyhHb and both control solutions, the host tissue blood saturation had relatively little change on boundary hypoxic volumes. This indicates that even though changes in host tissue blood saturation may be predictive in small animal models. They will likely fail is used as performance thresholds when translated to clinical trials. Fortunately, the trends in boundary hypoxic reduction as a function of the percent change in tumor Hb saturation remains relatively unchanged. Additionally, there is a linear correlation between the percent decrease in the boundary and total hypoxic volume.

**Fig 12:**
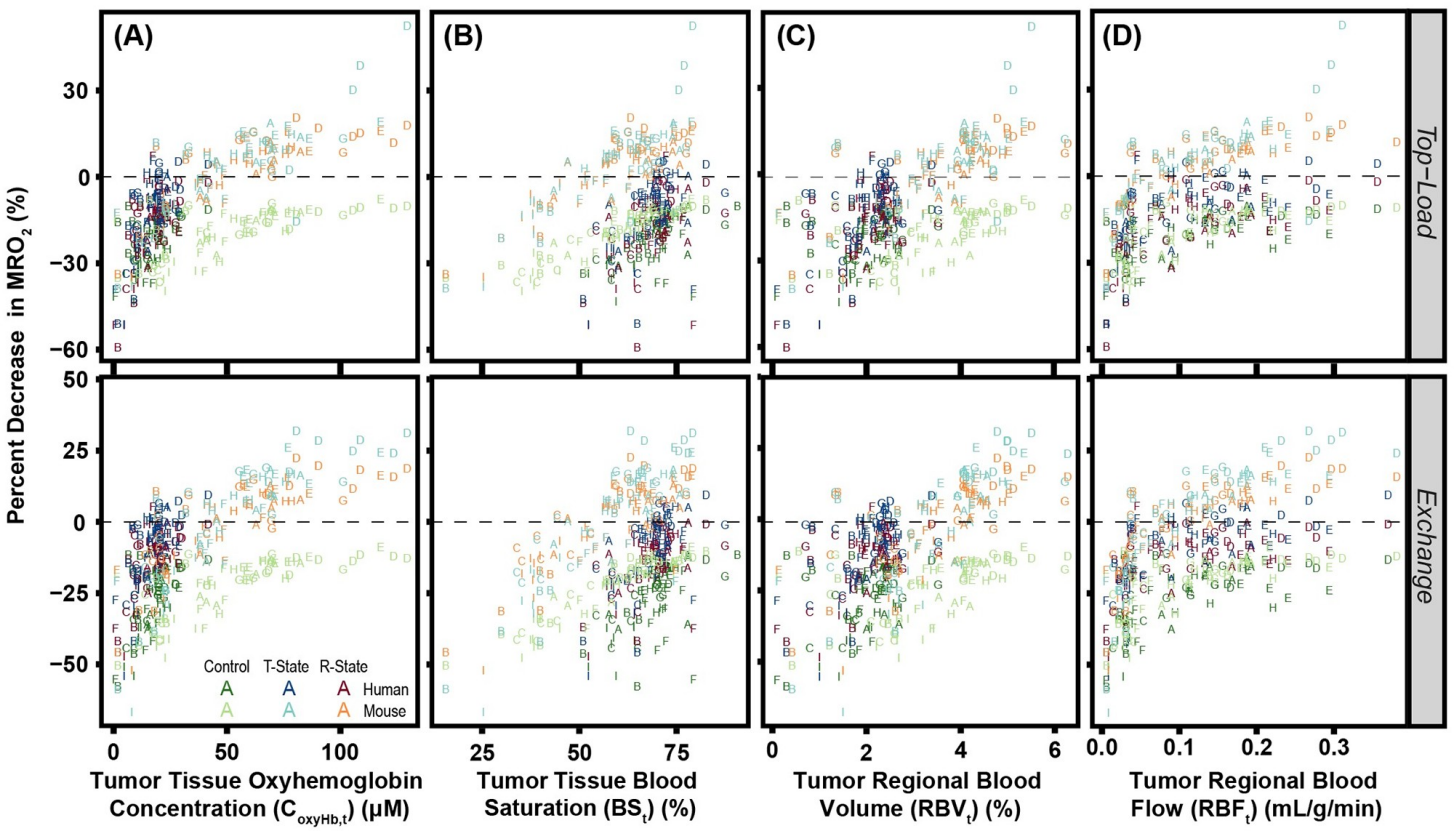
The effect tumor (A) oxyHb concentration, (B) tissue blood saturation, (C) *RBV*, and (D)*RBF* on the percent changes in *MRO*_2_. The baseline (unsupplemented) condition is depicted as the dashed line at zero. Letters labeling each data point indicate the vessel bed configuration for that artificial tumor construct.

The effects of tumor oxyHb concentration, tissue blood saturation, *RBV* and *RBF* on changes in *MRO*_2_ are shown in Fig 12. Unlike the hypoxic fraction, *MRO*_2_ can either increase or decrease depending on dose volume, infused material, and tumor vessel bed configuration. In general, *MRO*_2_ was increased in peripheral (type A, B, C, and I) vessel beds, whereas *MRO*_2_ was decreased in distal and proximal (type D—H) vessel beds. This implies that tumor location relative to arteries and veins has a substantial effect on the resulting changes in *MRO*_2_. Infusion of non O_2_ carrying controls always increases the *MRO*_2_ in both mouse and human models. This effect is dose dependent with increased deviation from the baseline after exchange infusion. For both R-State and T-State PolyhHb, there are similar increases in the peripheral tumors. For these vascular beds, infusion of R-State PolyhHb does not lead to significant change in *MRO*_2_. After infusion of T-State PolyhHb, *MRO*_2_ dramatically decreased in the distal and proximal tumors.

In the human model, pre-infusion tissue oxyHb concentration and *RBV* are insufficient to significantly decrease the *MRO*_2_. Similar to the effect of oxygenated Hb, we found that increasing pre-infusion O_2_ saturation led to a decrease in *MRO*_2_ change. Unlike *RBV* and oxyHb concentration, human and mouse data are not in agreement. At similar blood saturation values, the human model predicts an increase in *MRO*_2_ whereas the mouse model predicts a decrease. For each treatment option increasing the *RBF* decreases the change in the *MRO*_2_. For mouse tumors, we observed that *RBF* values greater than 0.1 mL/g/min may lead to reduction in *MRO*_2_.

## Discussion

The artificial tumors constructs generated in this study have significant variations in microvascular architecture. Despite observing a plateau in expansion rate, tumor growth is still approximately exponential. This is expected given that low tumor volumes (6.97 ± 5.11 mm^3^) place them within the exponential region of tumor xenograft growth [45, 46]. The tumors need to be at least ten times larger before host tissue nutrient supply limits growth. Fortunately, the tumor growth profile we observed matches data obtained with flat panel detector volume computed tomography (fpVCT) [47]. After simulating 40 days of tumor growth, most of the generated tumors have non-spheroid shapes with protrusions, oblong shapes, multiple clusters, and concave regions. As expected, tumor sections closer to arteries and large diameter arterioles tend to have less necrosis. Clusters of tumor growth have formed around these vessels as a result of increased regional O_2_ supply. These structural changes are similar to variations in the bulk architectural properties of *in vivo* tumors.

Despite HBOC nitric oxide (NO) scavenging altering shear sensitivity in the vascular network, there is relatively little variations in *RBV* after simulated HBOC administration. This likely results from hemodilution decreasing blood viscosity and thus increasing *RBF*. The increased *RBF* increases the shear modulus and stabilizes the vascular network.

### Oxygen transport

In mouse tumors we found that the angle between *C*_*Hb*,*tis*_ and *RBV* loading vectors is relatively small, which indicates these factors are closely correlated. This is expected, given that increasing blood volume will also lead to an increase in the total volume of RBCs and thus the total mass of Hb found within the tumor. In contrast, the blood pO_2_ vector is orthogonal to the necrotic percentage vector, which indicates that these variables are likely not correlated. Given that necrotic tissue does not consume O_2_ (maximum rate of O_2_ consumption (*V*_*M*_ = 0), we anticipated that increases in tumor necrotic fraction would have little effect on blood pO_2_. The various bulk tumor properties including tumor volume, *RBV*, *C*_*Hb*,*tis*_ and *RBF* are all tightly correlated. Additionally, tissue oxygenation variables are all partially correlated with both dose volume and bulk tumor properties. This indicates that both tumor structural properties and dose volumes influence tumor oxygenation.

Compared to the control grouping in mouse tumors, the control group in human tumors was more similar to the PolyhHb treatment group. Additionally, in human tumors there was no clear separation between different volumes of PolyhHb infusion. Both R-State PolyhHb treatment groups are closer to the control when compared to the corresponding dose of T-State PolyhHb. This difference between the two PolyhHb species may indicate that T-State PolyhHb leads to more alterations in tissue oxygenation compared to R-State PolyhHb infusion. This effect is likely results from the O_2_ affinity of T-State PolyhHb compared to the O_2_ affinity of Hb in mouse and human RBCs. Because both T-State and R-State have lower O_2_ affinity when compared to Hb in mouse RBCs they have similar performance. However, when compared to both R-State PolyhHb and the Hb in human RBCs, T-State PolyhHb has lower O_2_ affinity. Additional evidence for these effects is demonstrated in alterations to O_2_ saturation and *OEF* from Hb in RBCs and PolyhHb.

The values for RBC Hb O_2_ saturation in the human model is comparable to experimental data measured by Grosenick *et al.* (74 ± 12%) [28]. The baseline RBC Hb O_2_ saturation in the mouse model is significantly lower than the human RBC Hb O_2_ saturation. However it is comparable to baseline RBC Hb O_2_ saturation as measured in colon carcinomas in mice [18]. The change in Hb O_2_ saturation after T-State and R-State infusion determined in these simulations is also in agreement with the the O_2_ saturation of a low affinity (16 ± 10%) and high affinity (38 ± 8%) PEG-LEH as obtained with near infrared spectrocopy in a mouse colon carcinoma [18].

Similar to values of RBC Hb O_2_ saturation, PolyhHb O_2_ saturation is significantly lower in the human model when compared to the mouse model. This is likely a result of decreased *RBV* in the tumor coupled with an increase in RBC Hb O_2_ affinity in the human tumor model. Despite having a similar average vascular pO_2_ compared to the control, R-State PolyhHb appears to alleviate severe hypoxia is some tumors. After infusion of R-State PolyhHb in mouse tumors, the lowest average vascular pO_2_ increased to 6.15 mm Hg from the baseline value of 2.4 mm Hg. This increase was more than what was observed after simulated infusion of a T-State PolyhHb (4.15 mm Hg). It is important to note that these conditions were observed primarily within the poorly vascularized type B, C, and I tumors, which may not be applicable to more mature tumors. In the human model, we instead observed that R-State PolyhHb increased blood pO_2_ relative to the control similar to T-State PolyhHb in mice. In addition, T-State PolyhHb continued this trend with further increases in relative blood pO_2_ compared to both the control and R-State PolyhHb in the human model.

Finally, the *OEF* vectors and *RBF* vectors are divergent, which indicates that these variables are negatively correlated. Increasing *RBF* decreases blood residence time within the tumor and increases O_2_ mass transport through tumor tissue, which would lead to a decrease in O_2_ extracted from blood. If we compare these loading vectors, increasing dose volume is positively correlated with increasing tumor and host tissue pO_2_. The dose appears to be more closely correlated with boundary tumor tissue pO_2_ than with host tissue pO_2_. Although dose volume is also correlated with *OEF*_*HBOC*_ it is not correlated with the remaining *OEF*s. This is likely because tissue O_2_ demand is relatively unchanged even after increasing simulated dose volume. In the human model, T-State PolyhHb had much greater O_2_ extraction. In comparison, R-State PolyhHb had relatively similar O_2_ extraction in the mouse and human models. Additionally, the dose volume has negligible effect on *OEF*_*HBOC*_.

Despite delivering a greater fraction of its O_2_, T-State PolyhHb still delivers significantly less of its O_2_ compared to Hb in RBCs. This is expected because the partial pressure of O_2_ at which 50% of the hHb or PolyhHb is saturated with O_2_ (*P*_50_) of 35:1 T-State PolyhHb (34 mm Hg) is still less than *P*_50_ of mouse Hb in RBCs (42 mm Hg). Because of this increased *P*_50_, mouse Hb will, on average, deliver more O_2_ than T-State PolyhHb under elevated O_2_ tensions. Thus we expect that *OEF*_*HBOC*_ would increase for both of the species as the *OEF*_*Hb*_ approaches complete O_2_ delivery (*OEF*_*Hb*_ → 100%) under highly hypoxic conditions (pO_2_→ 0 mm Hg). Because of this increase in *OEF*_*HBOC*_ in the human models, we anticipate that T-State PolyhHb may be more effective at oxygenating tumors in humans.

### Tumor hypoxia

The lack of a dramatic decrease in overall tumor hypoxia is not in agreement with data from Teicher and Robinson *et al.* [5, 8, 9]. In these studies, the hypoxic fractions (< 5 mm Hg) under baseline conditions in the 13672 mammary carcinomas was 53% of tumor readings, and the 97 gliosarcomas was 49% of tumor readings. After infusion of low-affinity polymerized bovine Hb (bHb) (PolybHb) HBOC-201, these studies found the hypoxic readings decreased to 40% of total readings for the 13672 mammary carcinomas and 24% for the 9L gliosarcomas. However, the needle O_2_ electrode used in these studies had a maximum depth of 1 mm, which is still in the tumor periphery of 100 mm^3^ tumors.

In the boundary region between tumor and host tissue, we observed an increase in tissue pO_2_. However, there is considerably more hypoxic volume within this region. This primarily results from no longer including regions that are necrotic in our hypoxia evaluation within this region. These necrotic regions comprise a significant percentage (20 to 50%) of the simulated tumor mass. In both top-load and exchange infusion models, the presence of 35:1 T-State PolyhHb led to significant increases in boundary tissue pO_2_ and decreases in boundary tissue hypoxic volume fraction. This indicates that T-State PolyhHb is likely much more effective at oxygenating these tumors than either the non O_2_ carrying control or R-State PolyhHb.

Within this region, our results from the mouse model match trends observed by Teicher and Robinson *et al.* [5, 8, 9]. We also observed similar trends in the oxygenation for our model at the baseline pO_2_ (average of 12.5 mm Hg) and the exchange T-State PolyhHb infusion (18 mm Hg) compared to the early experimental data at the baseline (11.3 mm Hg) and the low-affinity HBOC infusion (20.8 mm Hg) [8]. This increase in the boundary region oxygenation may be related to the tumor growth delay observed after HBOC infusion in many of the previous experimental studies. Increased oxygenation of the tumor periphery can lead to increased survival of the host cells [48], which may result in the experimentally observed tumor growth delay.

Aditionally, the percent change in the total Hb saturation may be a predictor of increased hypoxia reduction for R-State PolyhHb. Decreases in the Hb saturation after T-State PolyhHb infusion translated to significant reduction in hypoxic volume. Unfortunately, this trend does not translate to R-State PolyhHb. For R-State PolyhHb there was no significant change as the tissue Hb saturation decreased. This is likely because the high O_2_ affinity R-State PolyhHb has relatively minor O_2_ release compared to T-State PolyhHb. Because of this, total Hb saturation is likely a poor marker to evaluate potential effectiveness of any high affinity O_2_ carrier for tumor oxygenation.

We also found the low *RBF* led to increased boundary hypoxia reduction. Typically, increases in the *RBF* is thought to increase tumor exposure to the circulated drug and thus its overall effectiveness [49]. This increase in efficacy is thought to occur due to an increase in O_2_, nutrient, and drug transport into tumor tissue. However, the reason we observed the performance enhancements associated with treatment models in this study is likely related to inadequate initial oxygenation of tumor tissue. For example, tumors with low initial *RBF* will likely be poorly oxygenated due to decreased exposure to circulating Hb in RBCs. Infusing a significant volume of PolyhHb increases the *RBF* via hemodilution, which will lead to greater decreases in the hypoxic volume of these tissues. Pre-infusion *RBF*s less than approximately 0.050 ml/(g min) in the top-load infusion model and 0.075 ml/g/min in the exchange infusion model were required but does not imply an increased reduction in the hypoxic volume.

### Metabolic rate of oxygen consumption

The tumor *MRO*_2_ was approximately two times higher in the human model when compared to the mouse model. This is in agreement with the increase in O_2_ consumption in humans under hypoxia [31, 50, 51]. This is likely a result of significantly decreased O_2_ release from the mouse Hb in RBCs under hypoxic conditions when compared to trends in the literature [31]. Additionally, the mouse host tissue *MRO*_2_ was significantly greater than human host tissue *MRO*_2_ (Data shown in S4 Appendix). Simulated infusion of the non-O_2_ carrying control lead to increases in the *MRO*_2_. The *MRO*_2_ was relatively similar between baseline and after T-State PolyhHb infusions.

Based on analysis of changes in tumor *MRO*_2_, we can predict that the proximal and distal tumors are typically more susceptible to *MRO*_2_ reduction compared to tumors growing in the periphery of arterial venous connections. This variance in *MRO*_2_ may contribute to the tumor growth delay observed in various animal studies [6, 8, 9]. Attenuating O_2_ consumption in tissue can also lead to decreases in the cell proliferation rate [52]. The oxyHb concentration would need to be above at least 45 μM before decreased *MRO*_2_ would be observed. Additionally, tumor *RBV* would likely need to be above 2.5 percent before these effects are observed in humans. In distal and proximal tumors (Types D-H), there is a dramatic divergence in the different treatment types depending on the oxyHb concentration. We also examined how pre-infusion Hb O_2_ saturation can predict changes in *MRO*_2_. The discontinuity between changes in mouse and human *MRO*_2_ indicates that comparing the tissue blood saturation between mice and humans may not fully represent the O_2_ status of the tissue. A similar trend is observed for the effect of tumor *RBF* on *MRO*_2_. For PolyhHb perfused human tumors and all control groups, we never observe a decrease in *MRO*_2_ compared to baseline conditions. This difference between tumors in different species is likely the result of the decreased Hb O_2_ affinity in murine RBCs which results in decreased *OEF* and *C*_*Hb*,*tis*_ despite increased *RBF* and O_2_ saturation. Thus, we predict that changes in *MRO*_2_ consumption should not be predicted via analysis of blood O_2_ saturation or *RBF*.

### Comparison with previous computational models

Previous computational models of vascular O_2_ transport to tumors extend upon previously developed Krogh tissue cylinder (KTC) models by instead modeling O_2_ and blood transport throughout an entire arterio-venous network connected by a capillary bed [53]. Early versions of these models used a Green’s function method to model O_2_ transport throughout a capillary network [54–56]. In general, these morphologically complex models gave more physiologically accurate estimations of hypoxia compared to the KTC models. [56]. This type of O_2_ transport model was used to explore the effects of changing the Hb O_2_ affinity on tissue oxygenation in vessels derived from confocal microscopy of an R3230AC mammary carcinoma in a 500 × 500 × 200 × μm space [57]. This study found that decreases in the *P*_50_ may lead to increased hypoxia in the tissue. Though this model did vary Hb O_2_ affinity, it did not explore the role of a variable heterogeneous tumor structure on the resulting O_2_ delivery. Additionally, this model only changed the *P*_50_ of the native Hb and did not consider the potentially complex dynamics between multiple O_2_ carrying species. To account for some of these effects, this model was translated to a complex network model with the addition of a low affinity diaspirin cross linked Hb (DCLB, *P*_50_ = 32 mm Hg, cooperativity coefficient (*n* = 2.4) and a genetically crosslinked recombinant Hb (rHb) 3261BR (*P*_50_ = 14.6 mm Hg, *n* = 2.15) [58]. These studies again predicted that decreasing Hb O_2_ affinity would lead to a significant increase in tissue pO_2_. In this study, Tsoukias *et al.* proposed that differences in the microvascular architecture may lead to drastically different results in different tissue types during infusion of either low- or high-affinity O_2_ carriers. Unfortunately, one of the weaknesses of these models is that they only consider a single tissue type for each vascular network (i.e. tumor or host tissue).

### Limitations of the simulations

Despite the quantity of applicable information gathered in these models, there are still some limitations resulting from the assumptions made during model construction documented in the S1 Appendix. For example, the continuum model for tumor expansion does not accurately model highly aggressive tumors that are typically associated with increased rates of metastasis. To handle this behavior, we would likely need to consider an agent based model. It may also be difficult to achieve stable intermediate constructs in the growth of a rapidly expanding tumor construct. To stabilize this system for analysis, we would need a significantly larger simulation domain to reduce edge effects. Despite development in the parallel application of this code, there is still a considerable computational cost associated with performing these simulations. For example, doubling the edge length results in an approximately 4× increase in the computational time and an 8× increase in the memory requirement. Therefore, to reach the 100 mm^3^ scale, the artificial tumor constructs would consume around 60× more resources per simulation.

Intratumoral acidosis is another factor which may have an impact on the results of the tumor simulations. Acidic pH in the tumor microenvironment and at the tumor-host boundary can result in tumor expansion and metastasis if the tumor cells are adapted to acidic conditions [59]. This acidic environment in tumors is primarily the result of increases in cellular respiration which results in increased production of carbon dioxide (CO_2_) and lactate [60]. Increased CO_2_ levels can result in significant reductions in Hb O_2_ affinity. This can lead to substantial increases in O_2_ delivery from Hb in RBCs due to the Bohr Effect [61]. Within tumors, increases in glycolysis boosts conversion of glucose to lactate. Lactate is directly related to the Warburg effect and is associated with a number of effects linked to inflammation, metastasis, and VEGF induction [62]. Lactate may also have an effect on radiotherapy and chemotherapy due to its antioxidant properties [63]. By modulating the *MRO*_2_ with administration of PolyHb, the pH and associated concentrations of CO_2_ and lactate may be altered within the tumor microenvironement. Because of these shifts, future models of tumor oxygenation should consider implementing systems of CO_2_ and lactate production as additional components within the simulation.

The architecture of this model may be limited by locking the vessel bifurcations in the model to a face-centered cubic grid. This grid significantly restricts the resulting angles of the vascular architecture, which may not adequately represent the correlations between daughter vessel size ratios and bifurcations that has been reported in the literature [64]. Despite the tendency of tumors to form leaky vasculature [65, 66], we did not model extravascular transport of the PolyhHb in this model. Currently, we have not observed any PolyhHb extravasation into the tumor tissue in experimental animal models. Because of this, we chose not to model extravascular transport of the PolyhHb solutions. If PolyhHb extravasation is observed in tumor tissues, these models should be repeated with the addition of extravascular PolyhHb transport in the tissue space. Additionally, we assumed that diffusive O_2_ transport is the dominant force. In some tumors, interstitial fluid flow may result in stronger extravascular O_2_ transport.

Due to limitations in the available computational resources, we were only able to numerically evaluate the effect of the PolyhHb infusions on a single type of cell line (FME human melanoma). Fortunately, the tumor architecture and bulk properties are similar to values measured in previous experimental studies. In the future, we could use anatomical data to explore our O_2_ transport models within an experimentally comparable vascular network.

Finally, our assumptions for the changes between human and mouse models only focused on changes in the microvascular density, blood flow, blood O_2_ offloading, and rate of O_2_ consumption in normal tissue. However, there may be other physiologic properties that change between the mouse and human models including inter-cellular interactions, varied production of vascular growth factors in mouse tissue, and differences in cellular composition in the host tissue.

### Conclusions

In this study, we performed simulations of PolyhHb mediated O_2_ transport within human and mouse artificial tumor vascular networks. These results were validated with data from experiments performed for this study and in the literature. We can use the models developed for this study to simulate the transport of O_2_ with both high and low-affinity HBOCs. The results from this mode of analysis can help highlight relationships between the vascular architecture and the ability of HBOCs like T-State and R-State PolyhHb to modulate tumor oxygenation. This makes the PolyhHb enhanced O_2_ transport model developed for this study a potential tool to evaluate and estimate clinically relevant benchmarks related to the translation of PolyhHb for use as a co-therapeutic in preclinical and clinical studies. Despite changes in the organism and group separation with PCA, the primary trends and variable correlations are relatively unchanged. This may indicate that biophysical parameters as measured in a mouse may still be useful to predict trends when translated into clinical studies. Motivated by our PCA and parameter analysis, we sought to observe how the correlated variables can be used to deduce which clinically measurable values might translate into treatment benchmarks. Performing simulations on artificial constructs provide the unique opportunity to explore how different treatment options would affect the O_2_ distribution and delivery given the same initial conditions. For example, our model suggests that tumors with decreased *RBF* and *RBV* are more susceptible to treatment with T-State and R-State PolyhHbs. However, we also found that large volume exchange infusion models are required to modulate hypoxia and *MRO*_2_ at a clinically relevant level.

Furthermore, we can use this model to help predict which clinically available non-invasive measurements should be taken to guide the development of PolyhHb for use in cancer treatment. Knowing the available techniques, we can then recommend targets and ranges to better test the efficacy and performance profiles of O_2_ carrying species as potential chemo-sensitizes. In our analysis of changes in the various bulk tumor properties, we determined that *MRO*_2_ was an exciting target for assessing the effect of HBOCs on tumor oxygenation and growth. In general, tumors that were more susceptible to hypoxia reduction likely had an increase in *MRO*_2_ after all treatment options. An increase in *MRO*_2_ may lead to increased effectiveness of chemotherapeutics that target rapidly dividing cells. Alternatively, reducing *MRO*_2_ and thus cell proliferation may be beneficial to limit tumor growth and thus, promote better drug delivery to tumors [52]. However, tumors that experienced a decrease in *MRO*_2_ typically had less reduction in the hypoxic volume. Because of this difference, future animal studies on the effect of PolyhHb enhanced oxygenation of tumors should consider the potential increase or decrease in *MRO*_2_ when selecting tumor types and model animals. Tumor locations should also be carefully considered to model these effects. From the results of this computational analysis, we recommend implanting the same tumor cell line in two locations: (1) a tissue region with high Hb concentration (*C*_*Hb*,*tis*_ = 50 μM) such as murine rear flank and (2) a tissue region with low Hb concentration (*C*_*Hb*,*tis*_ < 45 μM) such as murine mammary fat pad. During these studies, the transient changes in the concentrations of Hb and oxyHb in the host and tumor tissue should be measured throughout treatment using non invasive optical methods [67]. If possible, noninvasive methods for measuring *MRO*_2_ such as positron emission tomography (PET) [68, 69], magnetic resonance imaging (MRI) [70], or near infrared fluorescent dye [71] should also be considered.

## Methods

The simulations for this study were generated with a modified version of the Tumorcode simulation framework (https://github.com/thierry3000/tumorcode) [39]. The majority of simulations for this model were performed on the Owens Cluster at the Ohio Super Computing Center [72]. To simplify analysis of the resulting infusion models in artificial tumor constructs, various parameters relating oxygenation status and microvascular architecture were determined by iterating through completed files in Python v 3.6. Additional statistical comparisons such as t-tests and principal component analysis were performed with R v 3.6.0. Additional information on model construction and parameters can be found in the S1 Appendix.

### Polymerized hemoglobin enhanced vascular oxygen transport model

In this work, we expand upon the work of Welter *et. al*’s previously developed model of Hb facilitated O_2_ transport within the tumor microvascular architecture [39, 43]. With the addition of an HBOC, total blood O_2_ concentration (*C*_*O*_2_,*total*_) can be expressed as the sum of total O_2_ concentration dissolved in plasma (*C*_*O*_2_,*plasma*_), total O_2_ concentration bound to Hb in RBCs (*C*_*O*_2_,*RBC*_), and total O_2_ concentration bound to HBOC in the plasma (*C*_*O*_2_,*HBOC*_) as shown in Eq 1.

$$\begin{array}{c} C_{O_{2},total}=C_{O_{2},plasma}+C_{O_{2},RBC}+C_{O_{2},HBOC} \end{array}$$ (1)

The *C*_*O*_2_,*plasma*_ is proportional to pO_2_ depending on the solubility of O_2_ in plasma (*α*_*plasma*_). For O_2_ bound to Hb in RBCs we instead calculate *C*_*O*_2_, *RBC*_ by accounting for the concentration of Hb in RBCs (*C*_*Hb*,*RBC*_) on a heme basis. To calculate O_2_ bound to Hb in RBCs and HBOCs, we assumed that rate of O_2_ offloading (*k*_*off*,*O*_2__) is significantly faster than the residence time of Hb in RBCs and HBOCs within the vessels (Assumption 1). This would imply that hHb in RBCs and HBOCs are in equilibrium with the dissolved O_2_ (equilibrium saturation (*Y*) = current saturation (*S*). We can then calculate bound O_2_ by multiplying by hematocrit (*HCT*) and equilibrium saturation of O_2_ bound to Hb in RBCs as a function of the pO_2_ (*Y*_*Hb*_(*pO*_2_)) estimated with the Hill equation (Eq 2).

$$\begin{array}{c} Y=\frac{pO_{2}^{n}}{pO_{2}^{n}-P_{50}^{n}} \end{array}$$ (2)

The *C*_*O*_2_,*HBOC*_ can be calculated in a similar manner using the concentration of HBOC in the plasma (*C*_*HBOC*_) and equilibrium saturation of O_2_ bound to the HBOC in the plasma as a function of the pO_2_ (*Y*_*HBOC*_(*pO*_2_)). Performing these substitutions, we can then calculate *C*_*O*_2_,*total*_ as shown in Eq 3.

$$\begin{array}{c} C_{O_{2},total}\left(pO_{2}\right)=\alpha_{pls}pO_{2}+HCT\cdot C_{Hb,RBC}Y_{Hb,RBC}\left(pO_{2}\right)+\\ +C_{HBOC}Y_{HBOC}\left(pO_{2}\right) \end{array}$$ (3)

Continuing with the previously developed model, we approximate the intravascular pO_2_ as varying along the length (*z*) of the longitudinal axis of a cylindrical vascular tube segment with total length *l*. Therefore, we define the intravascular O_2_ flux (*j*_*O*_2_,*iv*_) as a product of the volumetric flow rate (*Q*) and *C*_*O*_2_,*total*_ as shown in Eq 4.

$$\begin{array}{c} j_{O_{2},iv}=QC_{O_{2},total}\left(pO_{2}\right) \end{array}$$ (4)

As blood flows through the vessel, a fraction of O_2_ is delivered from plasma, Hb in RBCs, and HBOC to surrounding tissue through the vessel wall. This value is quantified as the transavascular O_2_ flux (*j*_*O*_2_,*iv*_). This results in a change in the longitudinal O_2_ flux which is shown in Eq 5.

$$\begin{array}{c} \frac{dj_{O_{2},iv}}{dz}=Q(\alpha_{pls}\frac{dpO_{2}}{dz}+HCT\cdot C_{Hb,RBC}\frac{dY_{Hb,RBC}\left(pO_{2}\right)}{dP}\frac{dpO_{2}}{dz}\\ +C_{HBOC}\frac{dY_{HBOC}\left(pO_{2}\right)}{dP}\frac{dpO_{2}}{dz})=-2\pi rj_{O_{2},tv}\left(z\right) \end{array}$$ (5)

These equations can then be rearranged to solve for the derivative of pO_2_ along the longitudinal axis as shown in Eq 6.

$$\begin{array}{c} \frac{dP}{dz}=\frac{-2\pi rj_{O_{2},tv}\left(z\right)}{Q(\alpha_{pls}+HCT\cdot C_{Hb,RBC}\frac{dY_{Hb,RBC}\left(pO_{2}\right)}{dP}+C_{HBOC}\frac{dY_{HBOC}\left(pO_{2}\right)}{dP})} \end{array}$$ (6)

Similar to the previously developed 3D model, we are assuming that diffusive transport is dominant. This is also partially accounted for within the O_2_ transport Sherwood number $Sh_{O_{2}}$ correlation generated within the modified KTC model described in the S5 Appendix. To simplify *j*_*O*_2_,*iv*_ we use the mass transfer coefficient (*γ*) to relate *j*_*O*_2_,*iv*_ as the difference between pO_2_ in the blood (*pO*_2_(*z*) and pO_2_ in the tissue (*pO*_2,*tissue*_). Because vessels in the model are approximated as line sources, *pO*_2,*tissue*_ is approximated as being constant along the entire vessel segment. We can then calculate *γ* with the previously developed $Sh_{O_{2}}$, as shown in Eq 7.

$$\begin{array}{c} j_{o2_{t}v}=\frac{Sh_{O_{2}}D_{plasma}\alpha_{pls}\left(pO_{2}\left(z\right)-pO_{2,tissue}\right)}{2r} \end{array}$$ (7)

To calculate the vascular pO_2_ distribution we first isolate the mass balance of O_2_ around a single node with a set of adjacent upstream (I) and downstream (O) vessels. To simplify axial offloading, we assume that Hb in RBCs and HBOCs are in equilibrium with the common pO_2_ $p\tilde{O}$ at all outlets such that $pO_{2,j}=p{\tilde{O}}_{2}$ for j∈O (Eq 8).

$$\begin{array}{c} j_{O_{2},n}=\sum_{i\in I}Q_{i}\left(\alpha_{pls}pO_{2,i}+HCT_{i}C_{Hb,RBC}Y_{Hb,RBC}\left(pO_{2,i}\right)+C_{HBOC,i}Y_{HBOC}\left(pO_{2,i}\right)\right)\\ =\sum_{j\in O}Q_{j}(\alpha_{pls}p{\tilde{O}}_{2}+HCT_{j}C_{Hb,RBC}Y_{Hb,RBC}\left(p{\tilde{O}}_{2}\right)+C_{HBOC,j}Y_{HBOC}\left(p{\tilde{O}}_{2}\right) \end{array}$$ (8)

The O_2_ for the upstream vessels is known. Thus, rearranging the left side of Eq 8 we can obtain Eq 9.

$$\begin{array}{c} j_{O_{2},n}=[\sum_{j\in O}Q_{j}]\alpha_{pls}p{\tilde{O}}_{2}+[\sum_{j\in O}Q_{j}HCT_{j}]C_{Hb,RBC}Y_{Hb,RBC}\left(p{\tilde{O}}_{2}\right)\\ +[\sum_{j\in O}Q_{j}C_{HBOC,j}]Y_{HBOC}\left(p{\tilde{O}}_{2}\right) \end{array}$$ (9)

Here values in brackets correspond to the known blood, RBC, and HBOC flow rates determined from microvascular flow computation. The resulting Eq 9 can easily be solved with bisection methods.

To determine boundary conditions (BCs) for the mouse model, we began first with the simple blood vessel radius (*r*_*ves*_) dependent linear model developed by Welter *et al.* using the minimum inlet pO_2_ at the boundary (*pO*_2,*BC*,0_), maximum inlet pO_2_ at the boundary (*pO*_2,*BC*,*max*_), and rate of change of pO_2_ at the boundary (Δ*pO*_2,*BC*_ shown in Eq 10 [43].

$$\begin{array}{c} pO_{2,BC}=\text{min}\left(pO_{2,BC,0}+\Delta pO_{2,BC}r_{ves},pO_{2,BC,max}\right) \end{array}$$ (10)

Determining the pO_2_ distribution in the network can then be performed with a depth-first search, as described by Welter *et al.* [43]. The parameters used to populate the O_2_ transport model can be found in Table 1.

**Table 1:** Parameters used to compute PolyhHb enhanced oxygenation.

Symbol	Simulation Parameter	Value	Units	Source
*α*_*plasma*_	Solubility of O_2_ in plasma	1.71 × 10^−3^	mol/(m^3^ ⋅ mm Hg)	[57]
*α*_*tissue*_	Solubility of O_2_ in tissue	1.54 × 10^−3^	mol/(m^3^ ⋅ mm Hg)	[57]
*n*_*mHb*_	Cooperativity of murine Hb in murine RBCs	2.2	-	(a)
*P*_50,*mHb*_	*P*_50_ of murine Hb in murine RBCs	42.1	mm Hg	(a)
*n*_*hHb*_	Cooperativity of human Hb in human RBCs	2.9	-	[73]
*P*_50,*hHb*_	*P*_50_ of human Hb in humanRBCs	29.3	mm Hg	[73]
*D*_*O*_2_,*p*_	Diffusivity of O_2_ in plasma	1.85 × 10^−5^	cm^2^/s	[74]
*D*_*O*_2_,*t*_	Diffusivity of O_2_ in tissue space	6.30 × 10^−6^	cm^2^/s	[74]
*K*_*M*,*host*_	Michaelis constant for host tissue (mouse, human)	5	mm Hg	[57]
*K*_*M*,*tumor*_	Michaelis constant for tumor tissue	2	mm Hg	[57]
*V*_*M*,*mouse*_	Maximum rate of O_2_ consumption for mouse tissue	45	μM/s	[55]
*V*_*M*,*human*_	Maximum rate of O_2_ consumption for human tissue	15	μM/s	[55]
*V*_*M*,*tumor*_	Maximum rate of O_2_ consumption for tumor tissue	80	μM/s	[55]
*HCT*	Initial hematocrit	45	%	(a)
*pO*_2,*BC*,0_	Minimum inlet pO_2_ at the boundary	50	mm Hg	[75]
Δ*pO*_2,*BC*_	Rate of change of pO_2_ at the boundary	1	mm Hg / μm	[75]
*pO*_2,*BC*,*max*_	Maximum inlet pO_2_ at the boundary	100	mm Hg	[75]

^(a)^ Values determined with data from animal study performed in this study.

### Tissue oxygen consumption

For this system, the O_2_ concentration in the tissue (*C*_*O*_2_,*tissue*_) can be calculated by solving the steady-state diffusion equation on the tissue domain with Neumann boundary conditions with O_2_ exchange with vessels (*V*_*O*_2__) and the rate of O_2_ consumption (*R*_*O*_2__) as shown in Eq 11.

$$\begin{array}{c} \alpha_{tissue}D_{O_{2},tissue}\nabla^{2}pO_{2,tissue}-R_{O_{2}}\left(pO_{2,tissue}\right)+V_{O_{2}}=0 \end{array}$$ (11)

Here *R*_*O*_2__ is defined with the Michaelis-Menten model as defined in Eq 12.

$$\begin{array}{c} R_{O_{2}}=-\frac{V_{m}pO_{2}}{K_{M}+pO_{2}} \end{array}$$ (12)

Due to increased metabolism in mouse tissue compared to human tissue [76, 77], we assumed that the *V*_*M*_ of mouse tissue was approximately 3 times greater than the *V*_*M*_ for human tissue. We also assumed that the Michaelis-Menten coefficient (*K*_*M*_) remained unchanged between mice and humans.

*V*_*O*_2__ can be expressed as line sources with integration along the axis with respect to the Dirac (*δ*) distribution. These vessel segments are embedded within the tissue and can be expressed as shown in Eq 13 [56].

$$\begin{array}{c} V_{O_{2}}=\sum_{v\in V}\int_{v}2\pi r_{ves}j_{O_{2},tv}\delta \left(z-y\right)dz \end{array}$$ (13)

### Variations in simulation construction

For our infusion simulations, we considered two dose volumes (top-load, exchange) and three material types: a non O_2_ carrying control, a 35:1 T-State PolyhHb, and a 30:1 R-State PolyhHb. We also simulated a baseline condition where no treatment was delivered. Each of these 7 types of infusion simulations was performed on each simulated tumor construct modeled with the hypoxic (FME) tumor parameters. The biophysical properties of 35:1 T-State and 30:1 R-State PolyhHb were taken from the analysis performed on the PolyhHb prepared for use in the animal studies.

Unfortunately, T-State and R-State PolyhHb both have lower *P*_50_s compared to native mouse Hb in RBCs. Because of this, T-State PolyhHb delivers 45% less O_2_ and R-State PolyhHb delivers 96% less O_2_ than the native mouse RBCs in upstream arteries and arterioles at an equivalent concentration on a per-heme basis. Furthermore, the infusion mediated hemodilution results in a 10 to 22% decrease in O_2_ carried by blood. This reduction in O_2_ delivery indicates that the resulting pO_2_, at the BC (*pO*_2,*BC*_) is likely lower than the baseline (unsupplemented) condition. To calculate these adjusted values, we must assume that the maximum *pO*_2,*BC*_ is 100 mm Hg (assumption 8), and the tissue O_2_ consumption outside of the modeled tumor/host microvascular system is unchanged by hemodilution or HBOC infusion (assumption 9). Thus O_2_ extracted from blood between the lungs and the simulated system can be calculated by taking the O_2_ extracted as shown in Eq 14.

$$\begin{array}{c} OE=C_{O_{2},arteries}(pO_{2,BC,max}-C_{O_{2},BC}\left(pO_{2,BC}\left(r_{ves}\right)\right) \end{array}$$ (14)

Since we assume that O_2_ extraction upstream of the tumor is constant for both baseline, hemodilution, and HBOC infusion, we can solve for new coefficients for the linear correlation using Eq 15.

$$\begin{array}{c} OE_{baseline}\left(r_{ves}\right)=OE_{enhanced}\left(pO_{2},r_{ves}\right) \end{array}$$ (15)

In addition, we assume that despite its increased size, both 30:1 R-State and 35:1 T-State PolyhHb scavenge NO due to their increased exposure to the endothelial cell wall. This will lead to a decrease in the potency of the shear stress induced hemodynamic stimulus (*S*_*H*_) due to a reduction in the vasorelaxation signal. In experimental models, we observed that 30:1 R-State PolyhHb had a greater tendency to lead to vasoconstriction. Therefore *k*_*s*_ is further reduced for this species. We approximated each of these values using the data from intravital microscopy. The varied parameters for each of the simulations can be found in Table 2

**Table 2:** Parameter variations for top-load and exchange infusion simulations of a non O_2_ carrying control, 35:1 T-State PolyhHb, and R-State PolyhHb compared to baseline conditions.

Parameter	Baseline	*Top-Load infusion*			*Exchange infusion*		
		Control	35:1 T-State	30:1 R-State	Control	35:1 T-State	30:1 R-State
*HCT*	0.45	0.41	0.41	0.41	0.35	0.35	0.35
plasma viscosity (cP)	1.26	1.26	1.35	1.32	1.26	1.51	1.47
*C*_*HBOC*_ (mg/mL)	0	0	8	8	0	14	14
HBOC *P*_50_ (mm Hg)	-	-	34	1.3	-	34	1.3
HBOC *n*	-	-	1	1	-	1	1
*pO*_2,*BC*,0_ (mm Hg)	50	49.6	50.8	49.6	46.7	49.0	46.7
Δ*pO*_2,*BC*_ (mm Hg⋅*μ*m)	1.00	1.01	0.99	1.01	1.06	1.02	1.06
*k*_*s*_	1	1	0.96	0.95	1	0.95	0.90

### Quantifying bulk oxygenation data

As described previously, current tissue imaging techniques do not have adequate resolution to resolve the full vascular architecture of large three-dimensional tumors (> 10 mm^3^). Thus, to compare our simulated results to clinically measurable parameters, we must instead observe measurable tissue averaged properties. To calculate these bulk tumor properties, we iterate through each vessel (*v*) in the network of vessels (V) over the tissue volume (Ω). We use this method to calculate *MVD*, *RBV*, *RBF*, *C*_*Hb*,*tis*_), oxyHb concentration in the tissue (*C*_*oxyHb*,*tis*_), tissue Hb saturation (*S*_*Hb*,*tis*_), HBOC concentration in the tissue (*C*_*HBOC*,*tis*_), oxygenated HBOC concentration in the tissue (*C*_*oxyHBOC*,*tis*_), and tissue Hb saturation (*S*_*HBOC*,*tis*_). The full equations used to calculate these tissue properties can be found in S1 Appendix.

To examine functional O_2_ offloading for each of the treatment cases we calculate the percentage of O_2_ offloaded from each species across the full system to yield the *OEF*. For each of these systems, we calculate the mass flow of O_2_ into the system (*J*_*O*_2_, *in*_) through each of the inlet arterioles/arteries penetrating the surface of the system (v∈V∩I). We then subtract this value by the mass flow of O_2_ out of the system (*J*_*O*_2_, *out*_) through the outlet venules/veins penetrating the surface of the system (v∈V∩O) and divide by *J*_*O*_2_, *in*_. We also calculate O_2_ extracted from each O_2_ carrier (plasma, Hb in RBCs, and HBOC) to estimate how each contributes to overall O_2_ delivery to the tumor. For *OEF*_*plas*_ we can calculate the mass flow of dissolved O_2_ in plasma (*J*_*O*_2_, *plas*_) with *α*_*plasma*_, vascular *Q*, and vascular pO_2_ as shown in Eq 16.

$$\begin{array}{cc} OEF_{plas}= & \frac{J_{O_{2},in,pls}-J_{O_{2},out,pls}}{J_{O_{2},in,pls}}=\\ = & \frac{\alpha_{pls}\sum_{v\in V\cap I}Q_{v}pO_{2,v}-\alpha_{pls}\sum_{v\in V\cap O}Q_{v}pO_{2,v}}{\alpha_{pls}\sum_{v\in V\cap I}Q_{v}pO_{2,v}} \end{array}$$ (16)

We can also determine *OEF*_*Hb*_ with the mass flow of O_2_ bound to Hb in RBCs (*J*_*O*_2_, *Hb*_) using *C*_*Hb*,*RBC*_, vascular *HCT*, vascular *Q*, and *Y*_*Hb*_(*pO*_2_) as shown in Eq 17.

$$\begin{array}{cc} OEF_{Hb}= & \frac{J_{O_{2},in,Hb}-J_{O_{2},out,Hb}}{J_{O_{2},in,Hb}}=\\ = & \frac{C_{Hb,RBC}\sum_{v\in V\cap I}Q_{v}HCT_{v}Y_{Hb}\left(pO_{2,v}\right)-C_{Hb,RBC}\sum_{v\in V\cap O}Q_{v}HCT_{v}Y_{Hb}\left(pO_{2,v}\right)}{C_{Hb,RBC}\sum_{v\in V\cap I}Q_{v}HCT_{v}Y_{Hb}\left(pO_{2,v}\right)} \end{array}$$ (17)

The *OEF_HBOC_* can be calculated with a similar method using the vascular *C*_*HBOC*_ as shown in Eq 18.

$$\begin{array}{cc} OEF_{HBOC}= & \frac{J_{O_{2},in,HBOC}-J_{O_{2},out,HBOC}}{J_{O_{2},in,HBOC}}=\\ = & \frac{\sum_{v\in V\cap I}Q_{v}C_{HBOC,v}Y_{HBOC}\left(pO_{2,v}\right)-\sum_{v\in V\cap O}Q_{v}C_{HBOC,v}Y_{HBOC}\left(pO_{2,v}\right)}{\sum_{v\in V\cap I}Q_{v}C_{HBOC,v}Y_{HBOC}\left(pO_{2,v}\right)} \end{array}$$ (18)

We can then examine the total contribution of each O_2_ carrying species to the total *OEF* by taking a summation of the O_2_ mass flows into and out of the system as outlined in 19.

$$\begin{array}{c} OEF=\frac{J_{O_{2},in}-J_{O_{2},out}}{J_{O_{2},in}}=\\ =\frac{J_{O_{2},in,pls}+J_{O_{2},in,Hb}+J_{O_{2},in,HBOC}-J_{O_{2},out,pls}-J_{O_{2},out,Hb}-J_{O_{2},out,HBOC}}{J_{O_{2},in,plas}+J_{O_{2},in,Hb}+J_{O_{2},in,HBOC}} \end{array}$$ (19)

In addition to O_2_ delivery to the tissue, we can also examine *MRO*_2_ by taking the volume integral of the pO_2_ gradient dependent Michaelis-Menten equation (Eq 12) as shown in Eq 20.

$$\begin{array}{c} MRO_{2}=\frac{1}{\left|\Omega \right|}\int \Omega \frac{V_{M}pO_{2}\left(x\right)}{K_{M}+pO_{2}\left(x\right)}d^{3}x \end{array}$$ (20)

### Principal component analysis

Because there are a large number of variables associated with each tumor and treatment method, we employ PCA to linearly transform our data into a reduced factor space. All factors are scaled before PCA is performed. To perform this analysis, we used the prcomp function in R v. 3.6.0. The resulting data was then analyzed with PC score plots and loading factor analysis to better examine groupings and variable correlations.

### Statistical analysis

Data were represented as the mean ± SEM. All box plots depict the maximum, third quantile, median, first quantile, and minimum. Data were analyzed using a one way ANOVA with a Bonferroni’s correction for multiple comparisons. A p-value less than 0.05 was considered to be statistically significant between group comparisons. All statistical analysis was performed using R v. 3.6.0.

### Ethics statement

To validate the simulated O_2_ transport model, we compared the simulated results with fluid flow, vascular morphology, and pO_2_ measured with intravital microscopy. Mice were anesthetized with isoflurane (4% for induction, 1-2% for maintenance). All animals were euthanized with sodium pentobarbital. The protocols used to handle these mice were approved by the University of California San Diego Animal Care and Use Committee. The hHb used to prepare these materials was obtained from expired RBCs donated from Wexner Medical Center (Columbus, OH).

## Supporting information

S1 Appendix Additional description of the model. This file outlines additional segments of the model that have been applied from the Tumorcode framework including artificial blood vessel network generation, continuum model for tumor expansion, vascular remodeling during tumor growth, and quantifying bulk morphological data.
(PDF)

S2 Appendix Additional details on tumor growth and resulting properties. This file outlines the growth and resulting properties of the artificial mouse and tumor constucts described in this study.
(PDF)

S3 Appendix Synthesis and biophysical properties of T-State and R-State PolyhHb. This file documents the synthesis procedure and biophysical properties of the HBOCs prepared for this study.
(PDF)

S4 Appendix Comparison of mouse and human host tissue oxygenation and blood flow. This file documents the comparison between the oxygenation and blood flow observed between the simulated human and host tissue in which the artificial tumor constructs were produced.
(PDF)

S5 Appendix Determination of an oxygen transport Sherwood number. This file documents the method to determine the HBOC modified Sherwood number as estimated with linear regression on results from parametric sweeps on a KTC model.
(PDF)

S6 Appendix Computational model validation with animal experiments. This file documents an intravital microscopy and tumor growth experimental study that was used to validate the HBOC modified O_2_ transport model and vascularized tumor system used in this study.
(PDF)

S1 Dataset Dataset of the results from the oxygenation simulation. This file contains a dataset of the various tumor properties calculated in the hemoglobin-based oxygen carrier tumor simulation.
(CSV)
