# Supplementary material for: Tumor vascular status controls oxygen delivery facilitated by infused polymerized hemoglobins with varying oxygen affinity
Source: PLoS Comput Biol. 2020 Aug 20;16(8):e1008157. doi: 10.1371/journal.pcbi.1008157 (PMC7462268; doi:10.1371/journal.pcbi.1008157)
Supplement: S1 Appendix — This file outlines additional segments of the model that have been applied from the Tumorcode framework including artificial blood vessel network generation, continuum model for tumor expansion, vascular remodeling during tumor growth, and quantifying bulk morphological data. (PDF) [file pcbi.1008157.s001.pdf]

S1. Appendix. Additional description of model

Donald A. Belcher[1], Alfredo Lucas[2], Pedro Cabrales[2], Andre F. Palmer[1],

1 William G. Lowrie Department of Chemical and Biomolecular Engineering, The Ohio State University, Columbus, Ohio, USA
2 Department of Bioengineering, University of California, San Diego, La Jolla, CA, USA

Artificial Blood Vessel Network Generation

For this model, we continue with implementation of the three-dimensional artificial vascular network constructs as outlined by Welter *et al.* [1,2]. In brief, vessels are generated on a face-centered cubic lattice within a cubic tissue domain with a 6 mm edge length. Vessel generation begins at arterial and venous root nodes. An illustration of the various starting root node configurations for propagation of artificial blood vessel networks is shown in Fig A in S1 Appendix. Random elements consisting of linear or branching segments are added to each root node until vessel branches begin to overlap. Once vessel overlap occurs, connections are then formed between terminal vessels. These segments represent capillaries. Once vessels have been generated, the radii of terminal arterioles, capillaries, and venules are set to constant values. After this, the radii of the upstream and downstream vessel segments at branching connections are determined via Murray's Law.

$$r_p^{Mr} = r_1^{Mr} + r_2^{Mr} \quad (1)$$

Where r_p is the radius of the converging vessel, r_1 and r_2 are the radii of the diverging vessels, and Mr is a constant dependent on type of vessel (artery, vein) [3,4]. Blood flow is calculated throughout the network to determine the shear stress experienced by each vessel segment. Vessels with critically low shear stress (i.e., no perfusion) are pruned from the network. After vessels are removed, additional iterations of capillary generation/pruning are performed until the number of capillaries in the network stabilizes. After generation, this process is repeated a second time with 10× increased spatial resolution. The initial vessel network was generated with a lattice constant of 100 µm. An additional iteration was performed on the vascular network, which resulted in a lattice constant of 10 µm. Further parameters for the simulated artificial vascular network can be found in Table A in S1 Appendix.

Table A. Parameters used to compute the continuum model of tumor growth.

Symbol	Simulation Parameter	Value	Units	Source
$r_{cap,min}$	Minimum capillary radius	2.5	µm	-
$r_{art,min}$	Minimum arteriole radius	2.5	µm	-
$r_{ven,min}$	Minimum venule radius	3.8	µm	-
$r_{art,max}$	Maximum arteriole sprout radius	8.0	µm	-
$r_{ven,max}$	Maximum venule sprout radius	8.0	µm	-
MR_{art}	Murray's law constant for arteries and arterioles	3.00	-	[3,4]
MR_{ven}	Murray's law constant for veins and venules	2.74	-	[3,4]
$D_{\mathbb{V}}$	Edge-length of the simulation domain	6	mm	-
$h_{\mathbb{V}}$	Lattice constant of the vascular network	10	µm	-
$n_{\mathbb{V}}$	Size of the vascular network lattices	633×779×763	-	-

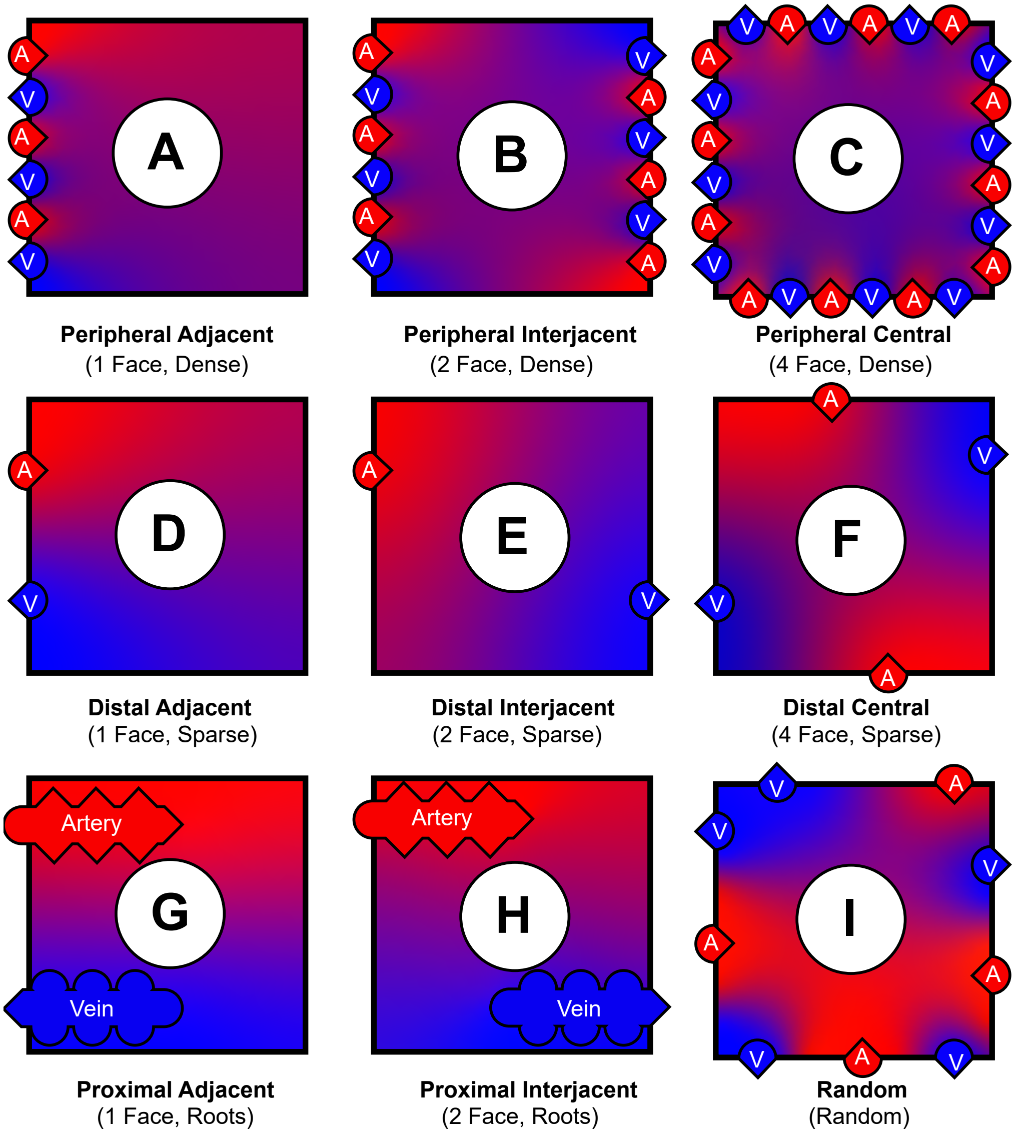

Fig A. Illustration of vascular root node configurations used as initial conditions for the simulations. Vascular bed types A, B, and C have alternating arterial (A) and venous (B) roots on each face of the cube. Bed types D, E, and F have nodes located at 33 % and 66 % along the face of the cube. Bed types G and H represent an artery or vein protruding into the tissue space. Bed types A, D, and G have roots only on a single face of the cube. Bed types B, E, and H have roots on two faces of the cube. Bed types C and F have roots on all 4 faces of the cube. Bed type I represents a cube with randomly generated root nodes that contain at least 1 arterial and 1 venous root. Modified from Welter M *et al.* PLOS ONE 2016 11(8): https://doi.org/10.1371/journal.pone.0161267 [2]

Previous studies have documented that murine tissue has higher rates of oxygen (O_2) consumption compared to human tissue. Because of the increase in O_2 consumption, healthy mouse tissue tends to have higher microvascular density (MVD) compared to human tissue [5]. To account for this difference in MVD, we varied the critical diffusion distance of O_2 (i.e., intercapillary distance) when generating vascular networks from 100 μm in human vascular generation to 45 μm in murine vascular generation . The decrease in O_2 diffusion in the murine model is directly related to the increased O_2 in healthy murine tissue [6, 7].

Continuum Model for Tumor Expansion

The model for tumor expansion is based on tissue models employed in the Tumorcode framework [1]. Here the tissue is modeled as a mixture of incompressible fluids including volume fraction of tumor cells (ϕ_T), volume fraction of normal cells (ϕ_N), volume fraction of necrotic cells (ϕ_D), volume fraction of the extracellular matrix (ECM) (ϕ_E), and volume fraction of the interstitial fluid (ϕ_F). Here volume fraction of cells (ϕ_C) can be expressed as a summation of various cell fractions ($\phi_C = \phi_T + \phi_N + \phi_D$). Additionally, ϕ_F is expressed as the remaining volume after the cells and matrix ($\phi_F = 1 - \phi_C - \phi_E$). We consider tumor cells and normal cells to be immiscibly separated at the interface of the tumor region (Ω_T) as defined in Equation 2.

$$\phi = \begin{cases} \phi_T + \phi_D & \in \Omega_T \\ \phi_N + \phi_D & \in \Omega \end{cases} \tag{2}$$

All cell fractions move along the cell fluid velocity field (v_ϕ) driven by a gradient in glspgrad as outlined by the momentum balance in Equations 3, 4, and 5 using cell mobility constant (K_ϕ), source term for cell growth (Q_ϕ), and source term for cell death (Q_D).

$$v_\phi = -K_\phi \nabla(\phi P)\phi \tag{3}$$

$$Q_\phi = \frac{\partial \phi}{\partial t} + \nabla \cdot (\phi v_\phi) \tag{4}$$

$$Q_D = \frac{\partial \phi_D}{\partial t} + \nabla \cdot (\phi_D v_\phi) \tag{5}$$

The corresponding values for Q_ϕ and Q_D are expressed as a summation of rate of cell expansion via proliferation (Q^P), rate of cell death via apoptosis (Q^A), and rate of cell death via necrosis (Q^D) as shown in Equations 6 and 7. Here when ϕ_N and ϕ_T die via necrosis, the corresponding volume fractions convert to ϕ_D which is unable to be cleared in the system. Apoptosis leads to complete elimination of cells.

$$Q_\phi = Q_N^P + Q_N^A + Q_N^D + Q_T^P + Q_T^A + Q_T^D \tag{6}$$

$$Q_D = -Q_T^D - Q_T^D \tag{7}$$

We model Q^P as a function of cell packing density (ϕ_C) and on the available nutrients in the environment (C). Here we model cell proliferation as a linear rate dependent on equilibrium density (homeostatic cell volume fraction (ϕ_0)), rate constant for cell proliferation (γ^P), and sensitivity to density variations (σ_ϕ^P). Additionally, we assume that at the critical low nutrient threshold for proliferation (ζ^P), all proliferative activity stops ($Q^P = 0$). Furthermore, when the nutrient concentration is suitably high, the rate constant for cell apoptosis (γ^A) is negligible. Taken together, this can be expressed as shown in Equation 8.

$$Q^P = \phi \max[\min\left[\frac{\gamma^P}{\sigma_\phi^P}(\phi_0 - \phi), \gamma^P \Theta(C - \zeta^P)\right], 0] \tag{8}$$

We can then calculate Q^A as a function of γ^A and cell fraction when the nutrient concentration is less than ζ^P, as shown in Equation 9.

$$Q^A = -\phi\gamma^A(1-\Theta(C-\zeta^P)) \tag{9}$$

Because necrosis only occurs when the nutrient concentration is below a critical low nutrient threshold for necrosis (ζ^D) ($C < \zeta^D < \zeta^P$), we use the smoothed Heaviside Function as shown in Equation 10 to calculate Q^D.

$$Q^D = -\phi\gamma^D(1-\Theta(C-\zeta^D)) \tag{10}$$

The rate of tumor expansion can then be described with a level set field on an auxiliary field ($\theta(x)$) of the distance (x) to the tumor interface such that $\theta(x) < 0$ for $x \in \Omega_T$ according to Equations 11 and 12.

$$\dot{x} \cdot n = v_\phi \cdot n \text{ for } x \in \partial\Omega_T \tag{11}$$

$$\frac{\partial \theta}{\partial t} + v_\phi \cdot \nabla\Theta = 0 \tag{12}$$

Where Θ is a smoothed Heaviside step function. The pressure can be expressed with a linear elastic system using the elastic modulus (E) where no pressure is exerted on cells when the ϕ_C is less than volume fraction of cells in the fully relaxed state (ϕ_R) as shown in Equation 13.

$$P_\phi(\phi_C) = \max(E(\phi_C - \phi_R), 0) \tag{13}$$

For this system, we follow the numerical implementation previously developed by Welter *et al.* [1]. The parameters used in this model can be found in Table B in S1 Appendix.

Table B. Parameters used to compute the continuum model of tumor growth modified from those used by Welter *et al.* [1]

Symbol	Simulation Parameter	Value	Units	Source
h_Ω	Tumor lattice constant	30	μm	-
n_Ω	Tumor lattice size	[211,225,227]	-	-
$R_{\Omega,0}$	Initial tumor diameter	500	μm	[1]
ϕ_E	ECM fraction	0.2	-	[1]
$K_\phi E$	Cell mobility × Eelastic modulus	5×10^3	μm^2/hr	[1]
γ_T^P	Tumor cell proliferation rate	1/24	hr^{-1}	[1]
γ_N^P	Normal cell proliferation rate	1/24	hr^{-1}	[1]
γ_T^A	Tumor cell apoptosis rate	0	hr^{-1}	[1]
γ_N^A	Normal cell apoptosis rate	1/240	hr^{-1}	[1]
γ_T^D	Tumor cell necrosis rate	1/48	hr^{-1}	[1]
γ_N^D	Normal cell necrosis rate	1/48	hr^{-1}	[1]
σ_ϕ	Cell pressure sensitivity	0.1	-	-
$\phi_{0,T}$	Tumor homeostatic cell fraction	0.6	-	[1]
$\phi_{0,N}$	Normal homeostatic cell fraction	0.4	-	[1]
ζ^P	Nutrient proliferation threshold	0.3	-	[1]
ζ^P	Nutrient necrosis threshold	0.03	-	[1]

Vascular Remodeling During Tumor Growth

As with the previously developed models, we continue to use the 5 part vascular remodeling system developed previously [2, 8, 9]. These 5 parts include: (1) angiogenic sprouting, (2) sprout progression, (3) circumferential expansion, (4) vessel wall degradation, and (5) vessel wall collapse. Angiogenic sprouting occurs on lattice sites occupied by vessels with a vessel tip cell sprouting probability (p_{sprout}) proportional to the concentration of vascular endothelial growth factor (VEGF) (C_{VEGF}) surrounding the vessel node, the vessel tip cell sprouting probability ($p_{sprout,max}$) and the characteristic time step (Δt) as described in Equation 14.

$$p_{sprout} = \frac{p_{sprout,max} C_{VEGF} \Delta t}{V_{sprout} + C_{VEGF}} \tag{14}$$

Once new sprouts have emerged, an exclusion radius is defined around the base of the vasculature where no new tip cells can be generated. Additionally, new sprouts are only able to emerge if there is an empty node available. Once the new sprout has emerged it can move to an adjacent node with a C_{VEGF} biased probability depending on the occupancy of surrounding nodes (N_j, N_k), lattice length scale (l_s), sprout cell mobility constant (K_{sprout}), sprout mobility constant (M_c), chemotactic sensitivity (γ_c), and Δt as shown in Equation 15.

$$p(i \rightarrow j) = \frac{\Delta t K_{sprout}}{l_s^2 \Delta x^2} \frac{(1-N_j)\left(1+\gamma_c \frac{C_{VEGF,i} - C_{VEGF,j}}{l_s \Delta x}\right)}{\sum_{k \in \Omega}(1-N_k) + Mc} \tag{15}$$

When environmental C_{VEGF} concentration exceeds critical VEGF concentration (ζ_{VEGF}), vessels switch from sprouting to circumferential growth. In this regime, we assume that endothelial cells (ECs) proliferate at a constant rate (time for EC proliferation (t_{EC}^{P})) leading to an increase in blood vessel radius (r_{ves}) up to an upper limit for the r_{ves} ($r_{ves,max}$). Inside the tumor, vessel wall stability (w) can degrade at a constant rate (Δw). Additionally, elevated solid pressure (P_ϕ) can lead to pinching of blood vessels if P_ϕ is less than P_{ves}, wall shear stress (τ_w) is less than critical wall shear stress ($\tau_{w,crit}$), and w is less than vessel wall stability (w_{crit}). Here we calculate τ_w using the difference in blood pressure (P) between connected nodes (ΔP), l_s, and r_{ves} as shown in Equation 16.

$$\tau_w = \frac{r_{ves} \Delta P}{l_s} \tag{16}$$

Assumptions

To prepare the model we made the following assumptions: (1) The rate of O_2 offloading (k_{off,O_2}) of hemoglobin (Hb) in red blood cells (RBCs) and Hb-based O_2 carriers (HBOCs) is considerably faster than the vessel residence time. (2) Zeroth order approximation that the variation of the partial pressure of dissolved O_2 (pO_2), hematocrit (HCT), and fluid velocity ($\bar{v}$) is negligible in radial direction as described previously. (3) Transvascular diffusive O_2 transport is dominant compared to transvascular convective O_2 transport. (4) Blood flow can be modeled according to Hagen-Poiseuille's law for flow through tubes. (5) The effective viscosity (μ_{eff}) of blood in microvessels varies according to the Fahraeus-Lindqvist effect. (6) RBCs tend to flow into branches with higher blood flow according to the Zweifach-Fung effect [10–13]. (7) HBOCs are small enough to maintain constant concentration within the plasma. (8) The maximum normal arterial pO_2 is 100 mm Hg. (9) Tissue O_2 consumption outside of the system is unchanged by hemodilution or HBOC transfusion.

Microvascular Flow Model

For this model, vascular segments are approximated as 1-dimensional tubular constructs connecting two nodes within the face-centered cubic lattice. Within this lattice, we calculate the

blood pressure (P) at each node along with blood volumetric flow rate (Q) and HCT for each vessel. Here Q is calculated according to the Hagen-Poiseuille law for flow through tubes using the vessel length (l), r_{ves}, plasma viscosity (μ_{plasma}), relative blood viscosity (μ_{rel}), and ΔP as shown in Eq 17 (Assumption 4). Here we assume that μ_{plasma} varies with the dose of HBOC as described previously [14].

$$Q = \frac{\pi r_{ves}^4 \Delta P}{8\mu_{rel}\mu_{plasma} l} \tag{17}$$

Where μ_{rel} is calculated with r_{ves} and HCT according to the Fahraeus-Lindqvist effect (Assumption 5) using the *in vivo* derived correlations for μ_{rel} developed by Pries *et al.* [15]. While this model was originally developed using data obtained from the rat mesentry, the viscosity of rate blood is relatively similar to human blood [16]. Thus, we continue to use it for simulating human blood flow in our model. The equations relating HCT and r_{ves} to μ_{rel} is shown in Eqs 18, 19 and 20.

$$\mu_{rel} = \left[1 + (\mu_{0.45}^* - 1) \cdot \frac{(1-H)^C - 1}{(1-0.45)^C - 1} \cdot \left(\frac{2r_{ves}}{2r_{ves} - 1.1}\right)^2\right] \cdot \left(\frac{2r_{ves}}{2r_{ves} - 1.1}\right)^2 \tag{18}$$

$$\mu_{0.45}^* = 6e^{-0.17 r_{ves}} + 3.2 - 2.44 e^{-0.12 r_{ves}^{0.645}} \tag{19}$$

$$C = (0.8 + e^{-0.15 r_{ves}}) \cdot (-1 + (1 + 10^{-11}(2r_{ves})^{12})^{-1}) + (1 + 10^{-11}(2r_{ves})^{12})^{-1} \tag{20}$$

The pressure at the boundary condition (BC) (P_{BC}) is set at a fixed value depending on r_{ves} and if the vessel is an artery ($A_3 = -1$) or vein ($A_3 = 1$) as defined by Eq 21 [2, 17, 18].

$$P_{BC}(r_{ves}) = A_2 + \frac{A_1 - A_2}{1 + e^{\left(\frac{A_3 r_{ves} - r_0}{\Delta r}\right)}} \tag{21}$$

Unfortunately, mice have much lower whole blood viscosity compared to both humans and rats [16, 19]. At equivalent hematocrits and low, the viscosity of mouse blood in microcirculatory networks is half of the viscosity of human and mouse blood. However; at sufficiently high shear rates the viscosity of mouse blood instead only around 80-90 percent that of human and mouse blood [16]. Given this, we assume that μ_{rel} is approximately 75 percent the value estimated by Pries *et al.* [20, 21]. This change to the blood viscosity calculation was verified with data from intravital microscopy described in a following section.
Additionally, we assume that RBCs in this model tend to flow into branches that have higher $\bar{v}$ in accordance with the Zweifach-Fung effect (Assumption 6) [10–13]. This phase separation effect results in heterogeneous distributions of HCT in daughter arterioles and capillaries. To calculate these effects, we use the correlations developed by Pries *et al.* [20]. We continue to use Tumorcode's built-in depth-first graph traversal algorithm to determine HCT distribution in the microvascular network [2, 22]. In addition, we assume that the diffusivity of HBOCs within the vascular network is high enough such that it has a constant concentration in the plasma phase (Assumption 7). This would imply that the total amount of Hb and thus O_2 bound to Hb may increase in poorly perfused vessels, as illustrated in Fig B in S1 Appendix.
To judge if this assumption was appropriate, we examined the Reynolds number (Re) and the Stokes number (Stk). In arterioles, capillaries, and venules, Re is very low (< 0.7) such that the Stokes flow approximation is appropriate [23]. Thus, Stk for the HBOC (polymerized human hemoglobin (hHb) (PolyhHb)) can be calculated by Eq 22.

$$Stk = \frac{\bar{v}\rho_{PolyhHb} D_{PolyhHb}}{36\mu_{plasma} r_{ves}} \tag{22}$$

Based on a PolyhHb density ($\rho_{PolyhHb}$) of 1 g/mL, a maximum observed hydrodynamic diameter of PolyhHb ($D_{PolyhHb}$) of 100 nm, arteriole $\bar{v}$ of 2 cm/s, enhanced μ_{plasma} of 1.8 cp

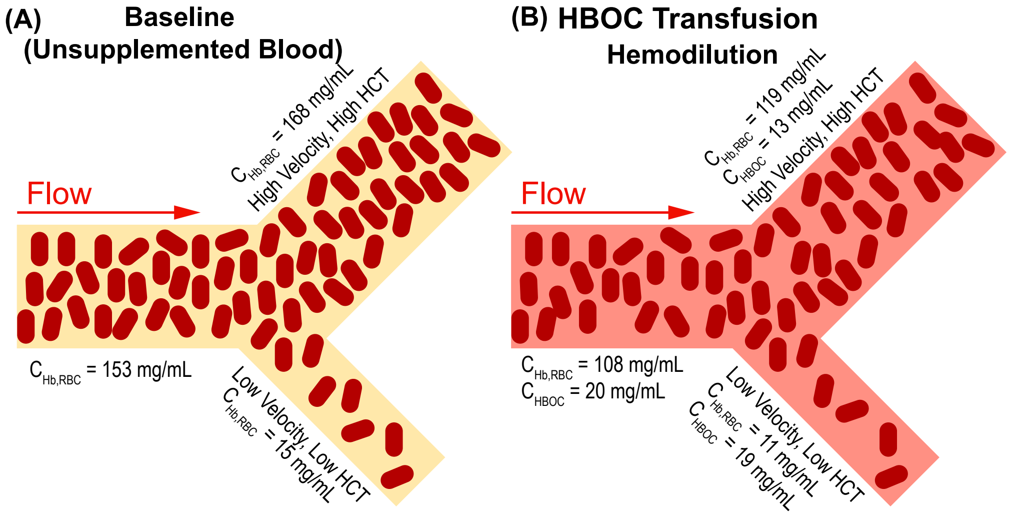

Fig B. Illustration of how HBOC perfusion will alter vascular Hb distributions. In the unspupplemented case (A) there is much less Hb available in the poorly perfused vessel. During HBOC transfusion, the HBOC in the plasma increases the total Hb available in the poorly perfused branch.

and a capillary diameter of 5 μm, the resulting Stk number is 1.2×10^{-6}. Since the resulting stokes number is small ($<< 1$), the motion of the HBOC is tightly coupled with fluid motion. Because of this, we coupled the concentration of HBOC in the plasma (C_{HBOC}) with the plasma volume as described in Eq 23.

$$C_{HBOC,v} = C_{HBOC,plasma}(1 - HCT) \tag{23}$$

Taking these systems together, we can perform a mass balance across the non-boundary nodes (v) accounting for total blood flow Q, RBCs (HCT), HBOCs(C_{HBOC}). as described in Eqs 24, 25, and 26.

$$\sum_v Q_v = 0 \tag{24}$$

$$\sum_v HCT_v Q_v = 0 \tag{25}$$

$$\sum_v C_{HBOC,v} Q_v = 0 \tag{26}$$

To solve this system, we use the Trilinos based Tumorcode solution package outlined by Welter and Fredrich *et al.* [2, 18].

Adaptation in the Vascular Network

Because the volume of HBOC transfusion results in a significant change in HCT and an increase in μ_{eff}, we must also take into account vascular adaptation due to smooth muscle cell stimulated contraction and relaxation. Here we employ the 3 part dynamic vascular adaptation model developed by Pries *et al.* [24]. For this model, we consider 3 stimuli: (1) the hemodynamic stimulus (S_H), (2) the metabolic stimulus (S_M),and (3) the conducted stimulus (S_C). In addition to these three stimuli, we model the tendency for the vessel to constrict as a constant. To update the vasculature, we assume that small changes in vasculature structure occur at a shorter timescale than endothelial cell division. Because surface area scales linearly with the radius, we limit this by restricting the final radius to no more than $1.75 \times$ the initial radius. We

can then model a small scale temporal evolution of the vascular segments using the vascular adaptation stimuli ($\sum S$) and the stimuli sensitivity (γ_s) as described in Eq 27.

$$r_{ves}(t+\Delta t) = r_{ves}(t)(1+\gamma_s \Delta t(S_H + S_M + S_C) - k_s) \tag{27}$$

S_H is calculated using τ_w, reference wall shear stress (τ_{ref}), and set point wall pressure ($\tau(\bar{P})$) as outlined in 28.

$$S_H = k_w \log(\tau_w) - k_p \log((\tau(\bar{P})) \tag{28}$$

The first term in this equation approximates the vasodilative effect of shear stress experienced by endothelial cells regulating smooth muscle cell relaxation [25]. The second term in the equation represents the stimuli from the transmural pressure, which balances vasorelaxation. This transmural stress is approximated with Eq 29

$$\tau(\bar{P}) = 100 - 86\exp(-5000[\log_{10}[\log_{10}(\bar{P})]]^{5.4}) \tag{29}$$

The S_M is used to approximate the metabolic needs of the tissue surrounding the vessel using the metabolic stimuli constant (k_{meta}), reference flow (Q_{ref}), Q, and HCT as shown in Eq 30.

$$S_M = k_{meta} \log\left(\frac{Q_{ref}}{Q \cdot HCT} + 1\right) \tag{30}$$

Where k_{meta} is a composite term that encompasses blood flow and changes in metabolic state. For example, if the metabolic demand of the tissue space outstrips the O_2 supply, then the segment can be stimulated to increase vascular flow. This effectively stabilizes the vascular diameters when the Q (and thus τ_w) is relatively low.

S_C represents the regulatory signals that propagate throughout the microvascular network. This stimuli is calculated by taking the sum of metabolic and conducted stimuli from downstream vessel segments. Here the conducted signal is assumed to decay exponentially along the network according to a length constant (L) as shown in Eq 31.

$$S_{C,f} = S_{M,A} + S_{M,B} + S_{c,a} e^{-x_a/L} + S_{c,b} e^{-x_b/L} \tag{31}$$

Because of the computational cost of this recursive system, S_C is damped with a reference constant (k_{cond}) and stimuli ($S_{C,ref}$) as defined in Eq 32.

$$S_C = k_{cond} \frac{S_{C,f}}{S_{C,f} + S_{C,ref}} \tag{32}$$

Each of these terms is balanced by the tendency of vessels to shrink without any stimuli (k_s). For normal tissue we use the parameters estimated by Pries *et al.* [24] and recently reverified by Fredrich *et al.* [26] However because these parameters have been obtained from healthy vascular tissue, they may not apply to tumor tissue characterized by aberrant microvascular structures. For example, S_C represents the ability of endothelial cells to communicate via electrical signals at the endothelial cell gap junction [27–29]. Within the tumor micro-environment, the structure of the endothelial cells, smooth muscle cells, and membrane is uncoordinated [30–32]. This can lead to a reduction in both electrical signals between cells (S_C) and vasorelaxation from the absence of smooth muscle cell coupling (k_s) Because of these characteristic changes within the tumor structure, we instead use the altered vascular stimuli within the tumor micro-environement determined by Secomb *et al.* [33]. The model parameters used in our adaptation model are shown in Table C in S1 Appendix.

Table C. Parameters used to compute transfusion enhanced microvascular adaptation.

Symbol	Simulation Parameter	Normal	Tumor	Units	Source
γ_s	Stimuli sensitivity	1	1	-	[24]
Δt	Adaptation timestep	0.1	0.1	-	[26]
k_w	Wall shear sensitivity constant	1	1	-	[24]
k_s	Transmural pressure sensitivity constant	1	1	-	[24]
k_{meta}	Metabolic sensitivity constant	0.83	0.83	-	[24]
Q_{ref}	Reference flow rate	40	40	nL/min	[24]
k_{cond}	Conducted sensitivity constant	2.74	0.2	-	[24, 33]
$S_{C,ref}$	Reference conducted stimuli	20	20	-	[24]
L	Reference length	1500	1500	μm	[24]
k_s	Shrinking constant	1.79	0.8	-	[24, 33]

Quantifying Bulk Morphological Data

We approximate MVD as the volume averaged length density using vessel l as shown in Equation 33.

$$MVD = \frac{1}{|\Omega|} \sum_{v \in \mathbb{V}} l_v \tag{33}$$

We approximate regional blood volume (RBV) by calculating the volume of each vessel with l and r_{ves} as shown in Equation 34.

$$RBV = \frac{1}{|\Omega|} \sum_{v \in \mathbb{V}} \pi r_{ves,v}^2 l_v \tag{34}$$

For regional blood flow (RBF) we take a volume average of Q through inlet vessel nodes at the boundary of the tissue space ($\mathbb{V} \cap \mathbb{I}$) as shown in Equation 35.

$$RBF = \frac{1}{|\Omega|} \sum_{v \in \mathbb{V} \cap \mathbb{I}} Q_v \tag{35}$$

In addition to calculating morphological data, we can also examine bulk properties associated with tissue Hb content and overall O_2 transport. Hb concentration in the tissue ($C_{Hb,tis}$) can be calculated with mean corpuscular Hb concentration ($MCHC$), HCT, and blood volume as defined in Equation 36.

$$C_{Hb,tis} = \frac{MCHC}{|\Omega|} \sum_{v \in \mathbb{V}} HCT \pi r^2 l_v \tag{36}$$

To calculate the percentage of Hb saturated with O_2 within the tumor we first calculate oxygenated Hb (oxyHb) concentration in the tissue ($C_{oxyHb,tis}$) by taking the integral of equilibrium saturation (Y) along vessel (v) as shown in Equation 37. We can then determine tissue Hb saturation ($S_{Hb,tis}$) by dividing $C_{oxyHb,tis}$ by $C_{Hb,tis}$ as shown in Equation 38.

$$C_{oxyHb,tis} = \frac{MCHC}{|\Omega|} \sum_{v \in \mathbb{V}} \left[HCT \pi r^2 \int_{l_v} Y_{Hb}(pO_{2,v}, z) dz \right] \tag{37}$$

$$S_{Hb,tis} = \frac{C_{oxyHb,tis}}{C_{Hb,tis}} \tag{38}$$

For the HBOC transfusions, we can calculate similar values for HBOC concentration in the tissue ($C_{HBOC,tis}$), oxygenated HBOC concentration in the tissue ($C_{oxyHBOC,tis}$), and tissue Hb saturation ($S_{HBOC,tis}$) using the blood vessel volume, C_{HBOC} and HBOC equilibrium saturation (Y_{HBOC}) as described in Equations 39, 40, and 41.

$$C_{HBOC,tis} = \frac{1}{|\Omega|} \sum_{v \in \mathbb{V}} C_{HBOC} \pi r^2 l_v \tag{39}$$

$$C_{oxyHBOC,tis} = \frac{1}{|\Omega|} \sum_{v \in \mathbb{V}} \left[C_{HBOC} \pi r^2 \int_{l_v} Y_{HBOC}(pO_{2,v}, z) dz \right] \tag{40}$$

$$S_{HBOC,tis} = \frac{C_{oxyHBOC,tis}}{C_{HBOC,tis}} \tag{41}$$

References

1. Welter M, Rieger H. Interstitial Fluid Flow and Drug Delivery in Vascularized Tumors: A Computational Model. PLoS ONE. 2013;8(8):e70395. doi:10.1371/journal.pone.0070395.

2. Welter M, Fredrich T, Rinneberg H, Rieger H, Hoff DV, Hingorani S. Computational Model for Tumor Oxygenation Applied to Clinical Data on Breast Tumor Hemoglobin Concentrations Suggests Vascular Dilatation and Compression. PLOS ONE. 2016;11(8):e0161267. doi:10.1371/journal.pone.0161267.

3. Sherman TF. On connecting large vessels to small. The meaning of Murray's law. The Journal of general physiology. 1981;78(4):431–53. doi:10.1085/jgp.78.4.431.

4. Luo T, Gast TJ, Vermeer TJ, Burns SA. Retinal Vascular Branching in Healthy and Diabetic Subjects. Investigative ophthalmology & visual science. 2017;58(5):2685–2694. doi:10.1167/iovs.17-21653.

5. Viacava P, Naccarato AG, Bocci G, Fanelli G, Aretini P, Lonobile A, et al. Angiogenesis and VEGF expression in pre-invasive lesions of the human breast. The Journal of Pathology. 2004;204(2):140–146. doi:10.1002/path.1626.

6. Wang Z, Zhang J, Ying Z, Heymsfield SB. Organ-Tissue Level Model of Resting Energy Expenditure Across Mammals: New Insights into Kleiber's Law. ISRN Zoology. 2012;2012:1–9. doi:10.5402/2012/673050.

7. Kummitha CM, Kalhan SC, Saidel GM, Lai N. Relating tissue/organ energy expenditure to metabolic fluxes in mouse and human: experimental data integrated with mathematical modeling. Physiological Reports. 2014;2(9):e12159. doi:10.14814/phy2.12159.

8. Olsen MM, Siegelmann HT. Multiscale Agent-based Model of Tumor Angiogenesis. Procedia Computer Science. 2013;18:1016–1025. doi:10.1016/J.PROCS.2013.05.267.

9. Owen MR, Alarcón T, Maini PK, Byrne HM. Angiogenesis and vascular remodelling in normal and cancerous tissues. Journal of Mathematical Biology. 2009;58(4-5):689–721. doi:10.1007/s00285-008-0213-z.

10. Fung YC. Stochastic flow in capillary blood vessels. Microvascular Research. 1973;5(1):34–48. doi:10.1016/S0026-2862(73)80005-6.

11. Obrist D, Weber B, Buck A, Jenny P. Red blood cell distribution in simplified capillary networks. Philosophical Transactions of the Royal Society A: Mathematical, Physical and Engineering Sciences. 2010;368(1921):2897–2918. doi:10.1098/rsta.2010.0045.

12. Li X, Popel AS, Karniadakis GE. Blood-plasma separation in Y-shaped bifurcating microfluidic channels: a dissipative particle dynamics simulation study. Physical biology. 2012;9(2):026010. doi:10.1088/1478-3975/9/2/026010.

13. Clavica F, Homsy A, Jeandupeux L, Obrist D. Red blood cell phase separation in symmetric and asymmetric microchannel networks: effect of capillary dilation and inflow velocity. Scientific reports. 2016;6:36763. doi:10.1038/srep36763.

14. Belcher DA, Ju JA, Baek JH, Yalamanoglu A, Buehler PW, Gilkes DM, et al. The quaternary state of polymerized human hemoglobin regulates oxygenation of breast cancer solid tumors: A theoretical and experimental study. PLoS ONE. 2018;13(2). doi:10.1371/journal.pone.0191275.

15. Pries AR, Secomb TW, Geßner T, Sperandio MB, Gross JF, Gaehtgens P. Resistance to blood flow in microvessels in vivo. Circulation Research. 1994;75(5):904–915. doi:10.1161/01.RES.75.5.904.

16. Windberger U, Bartholovitsch A, Plasenzotti R, Korak KJ, Heinze G. Whole Blood Viscosity, Plasma Viscosity and Erythrocyte Aggregation in Nine Mammalian Species: Reference Values and Comparison of Data. Experimental Physiology. 2003;88(3):431–440. doi:10.1113/eph8802496.

17. Pries AR, Secomb TW, Gaehtgens P. Design principles of vascular beds. Circulation research. 1995;77(5):1017–23.

18. Fredrich T, Welter M, Rieger H. Tumorcode: A framework to simulate vascularized tumors. European Physical Journal E. 2018;41(4):216903. doi:10.1140/epje/i2018-11659-x.

19. Vogel J, Kiessling I, Heinicke K, Stallmach T, Ossent P, Vogel O, et al. Transgenic mice overexpressing erythropoietin adapt to excessive erythrocytosis by regulating blood viscosity. Blood. 2003;102(6):2278–2284. doi:10.1182/blood-2003-01-0283.

20. Pries AR, Secomb TW, Gaehtgens P, Gross JF. Blood flow in microvascular networks. Experiments and simulation. Circulation Research. 1990;67(4):826–834. doi:10.1161/01.RES.67.4.826.

21. Pries AR, Neuhaus D, Gaehtgens P. Blood viscosity in tube flow: dependence on diameter and hematocrit. American Journal of Physiology-Heart and Circulatory Physiology. 1992;263(6):H1770–H1778. doi:10.1152/ajpheart.1992.263.6.H1770.

22. Welter M, Bartha K, Rieger H. Emergent vascular network inhomogeneities and resulting blood flow patterns in a growing tumor. Journal of Theoretical Biology. 2008;250(2):257–280. doi:10.1016/j.jtbi.2007.09.031.

23. Secomb TW. Blood Flow in the Microcirculation. Annual Review of Fluid Mechanics. 2017;49(1):443–461. doi:10.1146/annurev-fluid-010816-060302.

24. Pries AR, Secomb TW, Gaehtgens P. Structural adaptation and stability of microvascular networks: theory and simulations. American Journal of Physiology-Heart and Circulatory Physiology. 1998;275(2):H349–H360. doi:10.1152/ajpheart.1998.275.2.H349.

25. Cabrales P, Tsai AG, Frangos JA, Intaglietta M. Role of endothelial nitric oxide in microvascular oxygen delivery and consumption. Free Radical Biology and Medicine. 2005;39(9):1229–1237. doi:10.1016/j.freeradbiomed.2005.06.019.

26. Fredrich T, Welter M, Rieger H. Dynamic vessel adaptation in synthetic arteriovenous networks. bioRxiv. 2019; p. 429027. doi:10.1101/429027.

27. Pogoda K, Kameritsch P, Mannell H, Pohl U. Connexins in the control of vasomotor function. Acta Physiologica. 2019;225(1):e13108. doi:10.1111/apha.13108.

28. Segal SS. Cell-to-cell communication coordinates blood flow control. Hypertension. 1994;23(6_pt_2):1113–1120. doi:10.1161/01.HYP.23.6.1113.

29. Segal S. Regulation of Blood Flow in the Microcirculation. Microcirculation. 2005;12(1):33–45. doi:10.1080/10739680590895028.

30. Ribeiro AL, Okamoto OK. Combined effects of pericytes in the tumor microenvironment. Stem Cells International. 2015;2015:1–8. doi:10.1155/2015/868475.

31. Hashizume H, Baluk P, Morikawa S, McLean JW, Thurston G, Roberge S, et al. Openings between Defective Endothelial Cells Explain Tumor Vessel Leakiness. The American Journal of Pathology. 2000;156(4):1363–1380. doi:10.1016/S0002-9440(10)65006-7.

32. Carmeliet P, Jain RK. Angiogenesis in cancer and other diseases. Nature. 2000;407(6801):249–257. doi:10.1038/35025220.

33. Secomb TW, Dewhirst MW, Pries AR. Growth and Structural Adaptation of Blood Vessels in Normal and Tumor Tissues. In: New Perspectives in Mathematical Biology; 2010. p. 21.
