# Supplementary material for: Tumor vascular status controls oxygen delivery facilitated by infused polymerized hemoglobins with varying oxygen affinity
Source: PLoS Comput Biol. 2020 Aug 20;16(8):e1008157. doi: 10.1371/journal.pcbi.1008157 (PMC7462268; doi:10.1371/journal.pcbi.1008157)
Supplement: S2 Appendix — This file outlines the growth and resulting properties of the artificial mouse and tumor constucts described in this study. (PDF) [file pcbi.1008157.s002.pdf]

S2. Appendix. Additional details on tumor growth and resulting properties

Donald A. Belcher[1], Alfredo Lucas[2], Pedro Cabrales[2], Andre F. Palmer[1],

1 William G. Lowrie Department of Chemical and Biomolecular Engineering, The Ohio State University, Columbus, Ohio, USA
2 Department of Bioengineering, University of California, San Diego, La Jolla, CA, USA

Artificial Tumor Construct Growth

After polymerized human hemoglobin (hHb) (PolyhHb) enhanced oxygen (O_2) mass transport system was validated with data from intravital microscopy, we proceeded with generation of mouse and human 3D tumor constructs. Using the continuum model for tumor growth and the simplified vascular adaptation model, we generated 9 types (A-I) of tumors within the 6 mm artificial vascular networks. To better visualize how tumor architecture changes over time, we plotted cross sections of volume percentage of various components (vasculature, necrotic, and tumor) as the vessel grew. In Fig A in S2 Appendix we show these cross sections depicting progression of vascular network (blue), tumor tissue (green), and necrotic tissue (red) over 40 day growth period for a tumor grown in a murine type A vascular bed. Initially, the volume fraction of the vascular network is evenly distributed throughout the tissue space. As the tumor expands a region of rapid angiogenesis surrounds the expanding tumor mass. Additionally, tumor volume continues to expand for all 40 days, which eventually leads to a significant necrotic core without vascularization. For this tumor, the necrotic core begins in the top region of the tumor at day 10 and grows as the tumor continues to expand. At the end of 40 days, the selected tumor is around 2.5 mm in diameter. Visual observations confirms that this method of simulating tumor growth leads to a heterogeneous distribution of vasculature, tumor tissue, and necrotic tissue throughout the tumor mass. At day 40, bud like regions began to form at the tumor-host interface. This resulted in an uneven tumor-host tissue interface that is similar to previous experimental studies [1–3].

To observe how tumors progress during the simulated growth, we recorded various tumor properties including the tumor radius, rate of radial expansion, tumor sphericity, necrotic volume %, regional blood volume (RBV) and hemoglobin (Hb) concentration in the tissue ($C_{Hb,tis}$) every 48 hours during the growth of each artificial tumor construct. These growth curves are shown in Fig B for tumors grown in murine vascular beds. Upon analysis of this data, we found a correlative relationship in development of tumor RBV and tumor $C_{Hb,tis}$ during tumor expansion. Here, both tumor RBV and tumor $C_{Hb,tis}$ also increase rapidly during tumor expansion until they reach maximum saturation. This asymptotic behavior is tied directly to limitations of blood supply within host vascular networks. While there is a stable trend for $C_{Hb,tis}$ after initial growth, RBV gradually decreases as the tumor continues to grow. This is a result of vascular degradation within the tumor core leading to poorly vascularized regions within the tumor mass. Additionally, As tumors expand, sphericity tends to decrease as the surface deforms, and tumor shapes propagate towards regions with increased vascular perfusion. Tumor sphericity is calculated as a ratio of the tumor volume and the surface area ($Sph = \pi^{1/3}(6V_p)^{2/3}/A_p$)).

The rate of radial expansion for the Type D, E, and H was higher than the other vessel configurations. Despite this, radial expansion during tumor growth was not constant. In general, tumors rapidly expanded until they reach a stable rate of expansion, which differs for each vessel bed configuration. Tumor vessel configurations that have lower rates of radial expansion (0.5 µm /hr) tend also to have higher necrotic volumes (50 %). This is likely because tumor expansion is limited by O_2 delivery from host vascular networks. Despite observing a plateau in

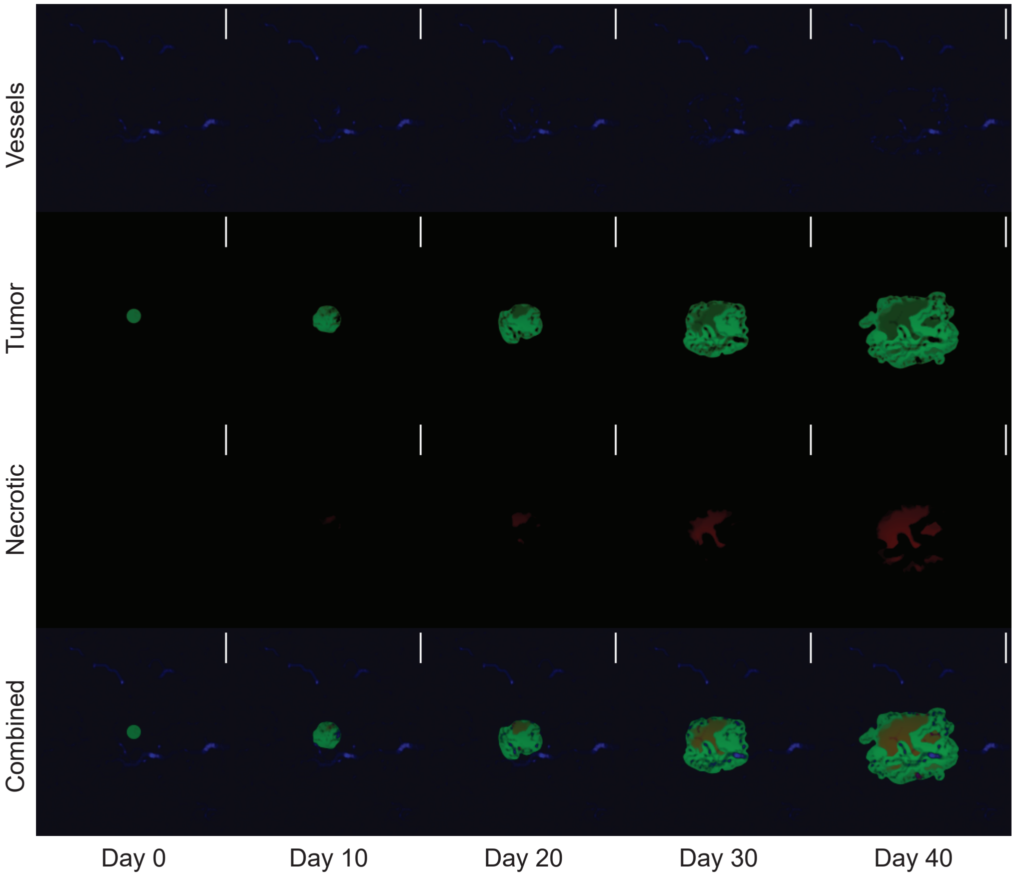

Fig A. Visualization of vessel (blue), tumor (green) and necrotic (red) volume fraction cross sections for artificial tumor grown in a mouse vascular network over 40 days. The tumor shown here was grown in a type A vascular bed. Scale bar = 1 mm.

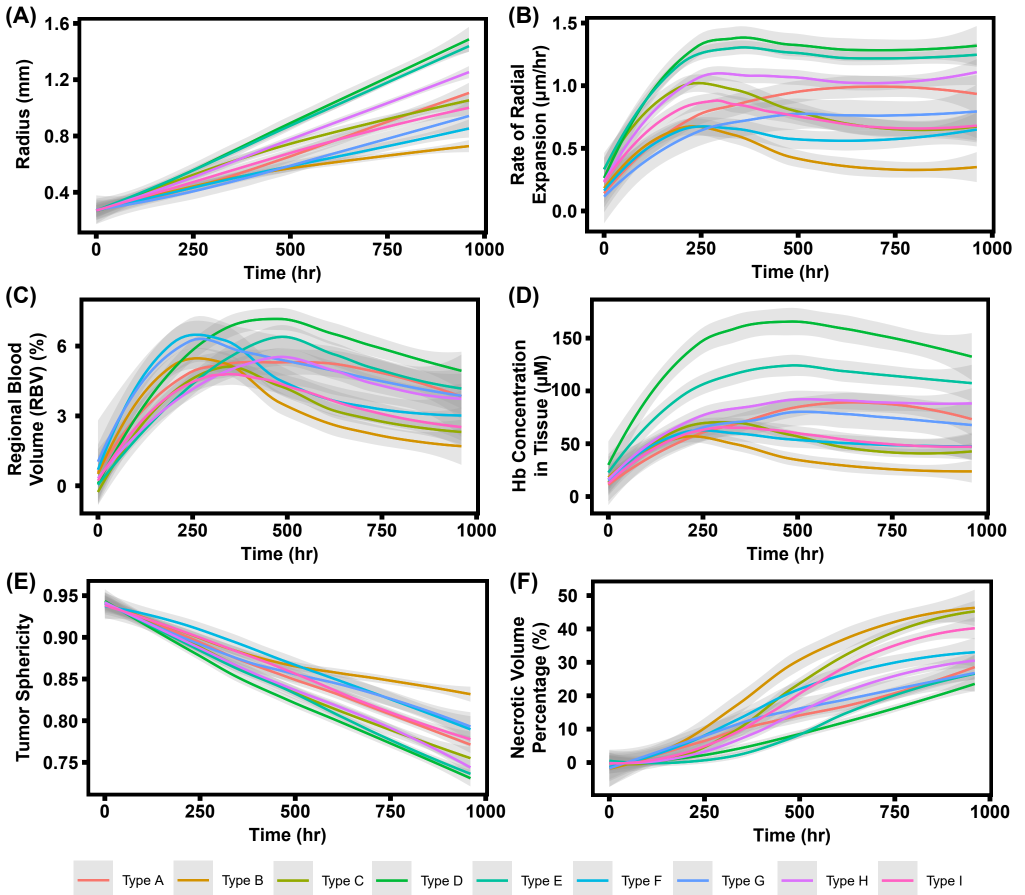

Fig B. Tumor growth and property curves during expansion of the artificial tumors over the 40 day growth period. In this figure the (A) radius, (B) rate of radial expansion, (C) RBV, (D) $C_{Hb,tis}$, (E) tumor sphericity, and necrotic volume percentages are shown for each vessel bed type (Type A-I). Shaded areas in the plot represent a 95% confidence interval across each type of vessel bed configuration.

the expansion rate, tumor growth is still approximately exponential. This is expected given that the low tumor volumes (6.97 $\pm$ 5.11 mm^3) place them within the exponential region of tumor growth [4, 5]. Tumors would need to be at least ten times larger before we would expect any growth limitations resulting from host tissue limitations. Fortunately, tumor growth profile we observed here matches the data obtained with flat panel detector volume computed tomography (fpVCT) [6].

Artificial Tumor Construct Cohort Properties

To visually observe spatial variance of resulting simulated tumor constructs, we plotted combined volume percentages (vascular:blue, tumor:green, necrotic:red) at the endpoint (t = 40 days) for each of tumor construct. Images of these tumors can be found in Fig C and D in S2 Appendix. Visual examination confirms that the generated artificial tumors are topologically diverse with significant variations between the vessel configurations. As mentioned previously, the type D, E, and H tumors are all significantly larger than the other tumor types ($p < 0.05$). In addition, the larger tumors tend to have a heterogeneous distribution of tumor, vasculature, and necrotic regions throughout the tumor mass. Smaller tumors tend to be more spheroid with a necrotic

core, low vascularization, and only active tumor cells in the periphery. Most of the generated tumors also tend to have interesting non spheroid shapes with protrusions (murine-E3), oblong shapes (murine-G7), multiple clusters (murine-F3), and concave regions (murine-A6). In general, it appears that tumor sections closer to arteries tend to have less necrosis. Additionally, clusters of tumor growth have formed around these regions as a result of increased regional O_2 supply.

After visually confirming the heterogeneous composition of the artificial tumor constructs, we analyzed how tumor growth impacted remodeling of vascular architecture. Selected volume averaged properties that described status of vascular networks are shown in Fig E and F. In general, tumor growth led to an increase in the microvascular density (MVD), RBV, vascular surface density, and $C_{Hb,tis}$. For each of these factors, increased values in the host tissue was required to obtain elevated values in the tumor tissue. Here the MVD (approximated as the length density) is a good indicator for number of vessels in tissues and is a good indicator of angiogenic progression in tumors. These values are in the range of the values from the micro CT data from Stametalos *et al.* (9-101 mm^{-2}) [7], the immunohistochemical count data from Dhakal *et al.* (0-157 mm^{-2}) [8], and immunohistochemical count data from Fernández-Guinea *et al.* (9-100 mm^{-2}) [9].

RBV values are in agreement with micro CT data from Stametalos *et al.* (0.2 - 3.4 %) [7], values measured with optical mammography by Grosenick *et al.* (2.4 $\pm$ 1.5 %) [10], and values measured with positron emission tomography by Bleaney (4.3 $\pm$ 1.3 %) [11]. To compare the rate of angiogenesis to the MVD, we calculated the vascular surface density as a composite of the MVD and RBV. The vascular surface density follows a similar trend to the MVD and RBV. We observe stable values for the surface density (2.2 $\pm$ 0.45 mm^{-1}), which is in the range of the micro CT data from Stametalos *et al.* (0.5-5.4 mm^{-1}) [7].

Compared to the host MVD, RBV and vascular density, the host $C_{Hb,tis}$ has distinct grouping based on the vessel bed configuration. Vessel bed types B, C, and I each have lower $C_{Hb,tis}$ (18 $\pm$ 1.8 μM) than the other vessel types. Despite this variation in the host $C_{Hb,tis}$, the tumor $C_{Hb,tis}$ is much more evenly distributed than the host $C_{Hb,tis}$. These values for the tumor $C_{Hb,tis}$ are comparable with optical mammography data measured by Grosenick *et al.* (53 $\pm$ 32 μM) [10] and values measured by Spinelli *et al.* (70 $\pm$ 35 μM) [12]. However, if we take only the values for the larger tumors grown in vessel beds D, E, and H, $C_{Hb,tis}$ (62 $\pm$ 19 μM) is in agreement with Spinelli's values.

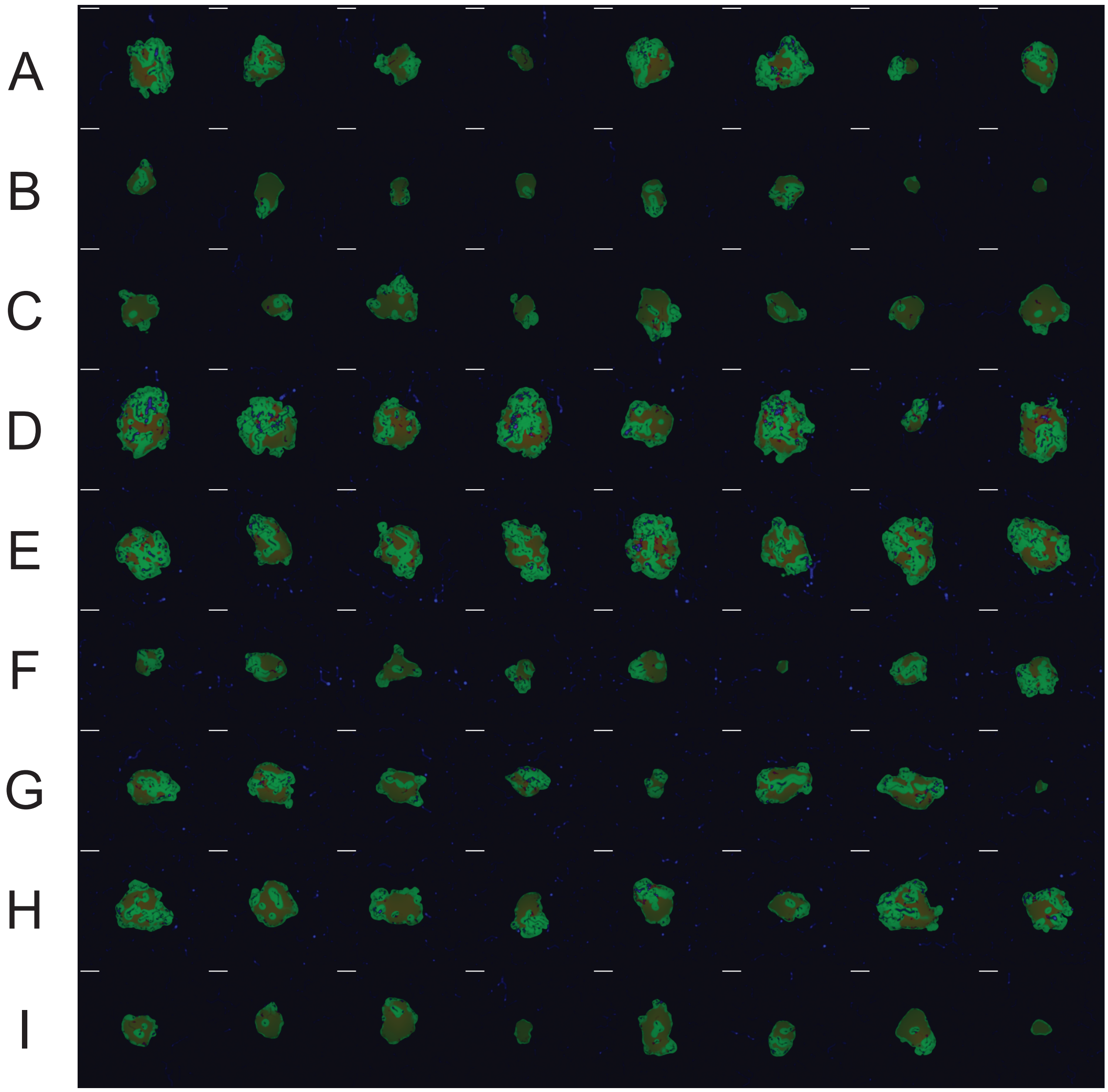

Fig C. Visualization of combined volume fraction (blue:vessel, green:tumor, red:necrotic) cross sections for each artificial murine tumor construct. Each row contains tumors for each of the vessel configurations described in the microvascular network section. Scale bar = 1 mm.

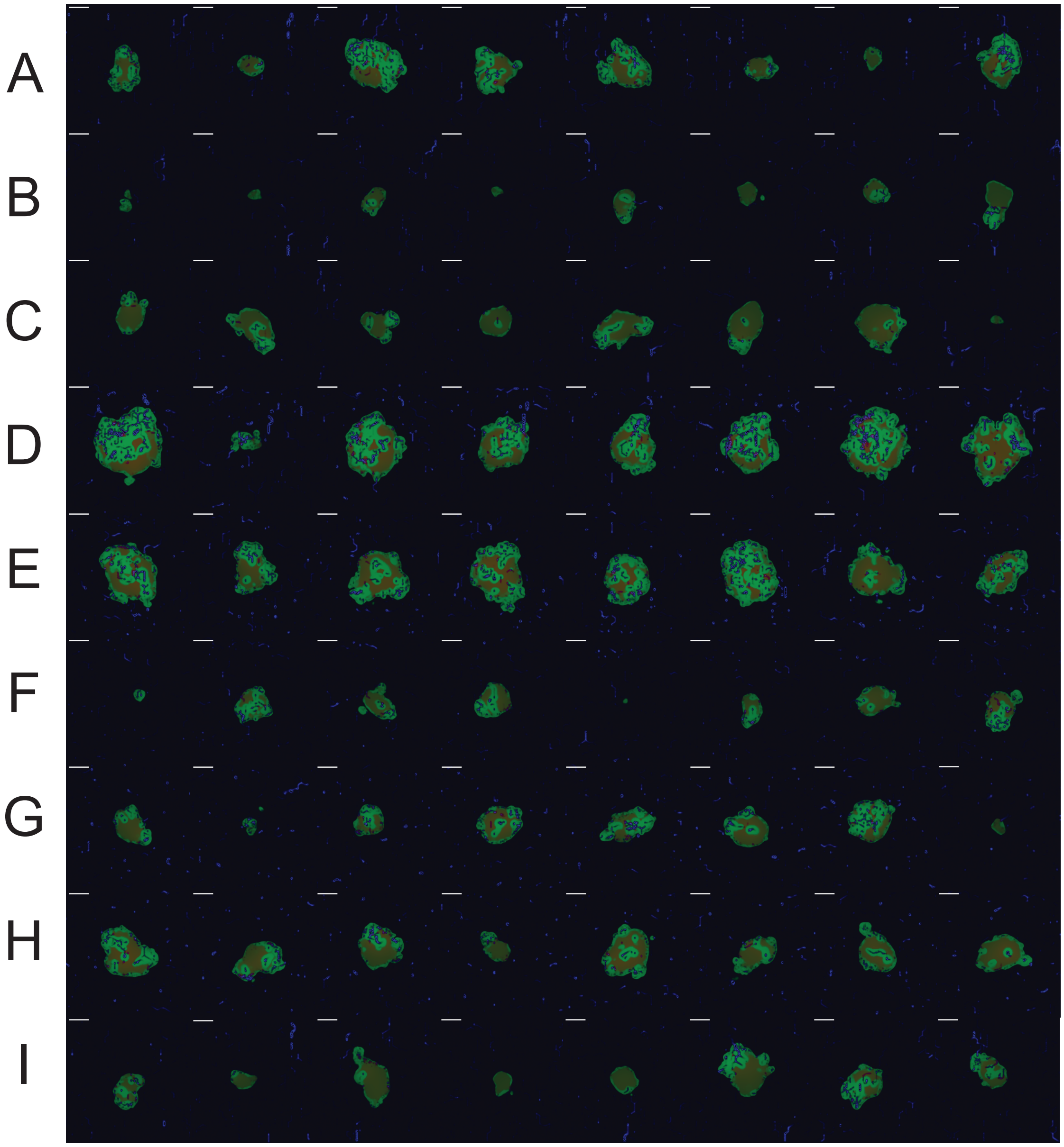

Fig D. Visualization of combined volume fraction (blue:vessel, green:tumor, red:necrotic) cross sections for each artificial human tumor construct. Each row contains tumors for each of the vessel configurations described in the microvascular network section. Scale bar = 1 mm.

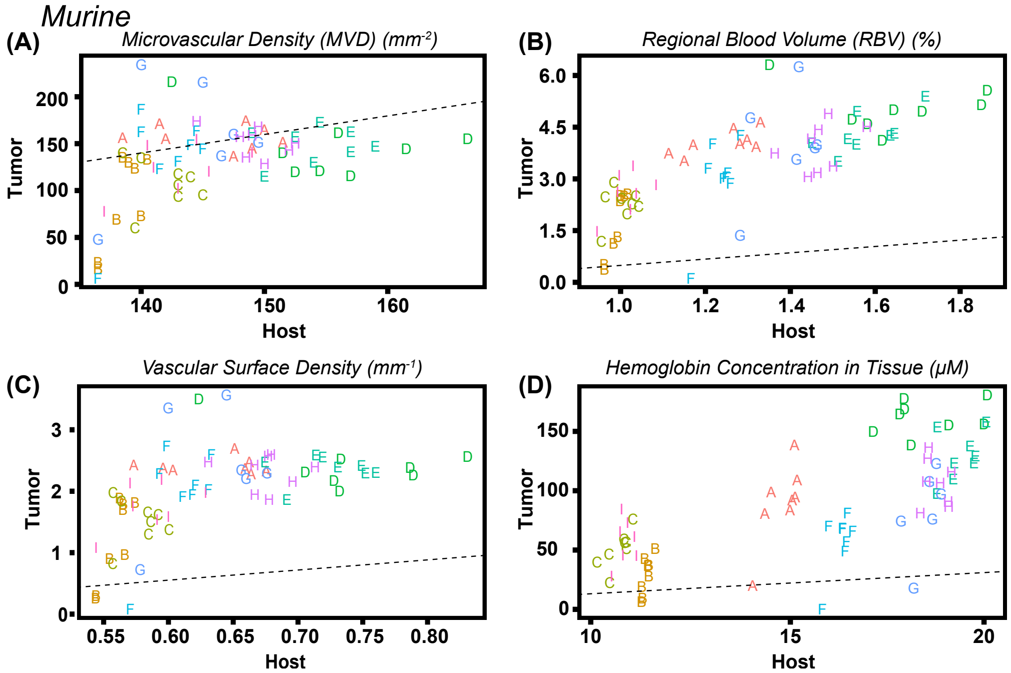

Fig E. Comparison between the (A) MVD approximated as the length density, (B) RBV, (C) vascular surface density, and (D) $C_{Hb,tis}$ between mouse tumors and host tissue in artificial tumor constructs. Letter labels indicate the vessel configuration. Dashed line separates the tumor properties greater than and less than the host properties

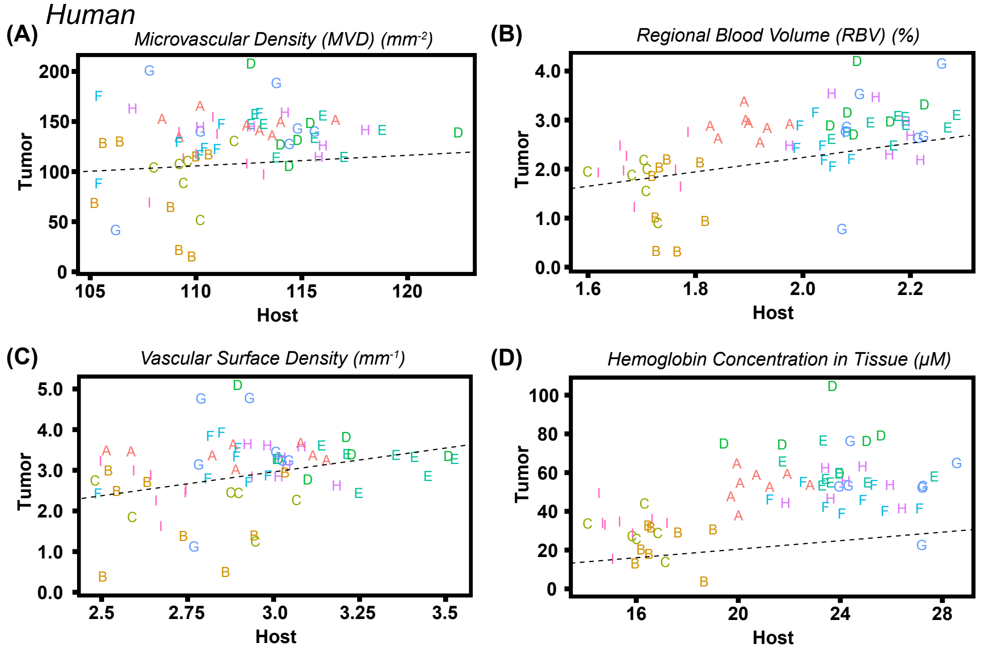

Fig F. Comparison between the (A) MVD approximated as the length density, (B) RBV, (C) vascular surface density, and (D) $C_{Hb,tis}$ between human tumor and host tissue in artificial tumor constructs. Letter labels indicate the vessel configuration. Dashed line separates the tumor properties greater than and less than the host properties

References

1. Demir C, Gultekin SH, Yener B. Learning the topological properties of brain tumors. IEEE/ACM Transactions on Computational Biology and Bioinformatics. 2005;2(3):262–269. doi:10.1109/TCBB.2005.42.

2. Rangayyan RM, Nguyen TM. Fractal analysis of contours of breast masses in mammograms. Journal of Digital Imaging. 2007;20(3):223–237. doi:10.1007/s10278-006-0860-9.

3. Nguyen TM, Rangayyan RM. Shape Analysis of Breast Masses in Mammograms via the Fractal Dimension. In: 2005 IEEE Engineering in Medicine and Biology 27th Annual Conference. IEEE; 2005. p. 3210–3213. Available from: `http://ieeexplore.ieee.org/document/1617159/`.

4. Worschech A, Chen N, Yu YA, Zhang Q, Pos Z, Weibel S, et al. Systemic treatment of xenografts with vaccinia virus GLV-1h68 reveals the immunologic facet of oncolytic therapy. BMC genomics. 2009;10:301. doi:10.1186/1471-2164-10-301.

5. Murphy H, Jaafari H, Dobrovolny HM. Differences in predictions of ODE models of tumor growth: a cautionary example. BMC Cancer. 2016;16(1):163. doi:10.1186/s12885-016-2164-x.

6. Missbach-Guentner J, Dullin C, Kimmina S, Zientkowska M, Domeyer-Missbach M, Malz C, et al. Morphologic changes of mammary carcinomas in mice over time as monitored by flat-panel detector volume computed tomography. Neoplasia. 2008;10(7):663–673. doi:10.1593/neo.08270.

7. Stamatelos SK, Kim E, Pathak AP, Popel AS. A Bioimage Informatics Based Reconstruction of Breast Tumor Microvasculature with Computational Blood Flow Predictions. Microvascular research. 2014;91:8. doi:10.1016/J.MVR.2013.12.003.

8. Dhakal HP, Bassarova A, Naume B, Synnestvedt M, Borgen E, Kaaresen R, et al. Breast carcinoma vascularity: a comparison of manual microvessel count and Chalkley count. Histology and histopathology. 2009;24(8):1049–59. doi:10.14670/HH-24.1049.

9. Fernández-Guinea O, Álvarez-Cofiño A, Eiró N, González LO, del Casar JM, Fernandez-Garcia B, et al. Low microvascular density at the tumor center is related to the expression of metalloproteases and their inhibitors and with the occurrence of distant metastasis in breast carcinomas. International Journal of Clinical Oncology. 2013;18(4):629–640. doi:10.1007/s10147-012-0428-2.

10. Grosenick D, Wabnitz H, Moesta KT, Mucke J, Schlag PM, Rinneberg H. Time-domain scanning optical mammography: II. Optical properties and tissue parameters of 87 carcinomas. Physics in Medicine and Biology. 2005;50(11):2451–2468. doi:10.1088/0031-9155/50/11/002.

11. Beaney RP. Positron emission tomography in the study of human tumors. Seminars in Nuclear Medicine. 1984;14(4):324–341. doi:10.1016/S0001-2998(84)80006-9.

12. Spinelli L, Torricelli A, Pifferi A, Taroni P, Danesini G, Cubeddu R. Characterization of female breast lesions from multi-wavelength time-resolved optical mammography. Physics in Medicine and Biology. 2005;50(11):2489–2502. doi:10.1088/0031-9155/50/11/004.
