# Supplementary material for: Tumor vascular status controls oxygen delivery facilitated by infused polymerized hemoglobins with varying oxygen affinity
Source: PLoS Comput Biol. 2020 Aug 20;16(8):e1008157. doi: 10.1371/journal.pcbi.1008157 (PMC7462268; doi:10.1371/journal.pcbi.1008157)
Supplement: S3 Appendix — This file documents the synthesis procedure and biophysical properties of the HBOCs prepared for this study. (PDF) [file pcbi.1008157.s003.pdf]

S3 Appendix. Synthesis and biophysical properties of T-State and R-State PolyhHb

Donald A. Belcher[1], Alfredo Lucas[2], Pedro Cabrales[2], Andre F. Palmer[1],

1 William G. Lowrie Department of Chemical and Biomolecular Engineering, The Ohio State University, Columbus, Ohio, USA
2 Department of Bioengineering, University of California, San Diego, La Jolla, CA, USA

Methods

PolyhHb synthesis

Human hemoglobin (hHb) used in this process was purified from human red blood cells (RBCs) using protocols developed by Palmer *et al.* [1]. Deoxygenated hemoglobin (Hb) (deoxyHb) and oxygenated Hb (oxyHb) were polymerized with glutaraldehyde using methods developed previously [2–4]. To prepare 35:1 (molar ratio of glutaraldehyde to hHb) tense quaternary state (T-State) (deoxygenated) polymerized hHb (PolyhHb), the hHb solution is devoid of dissolved oxygen (O_2) before and during the polymerization reaction. The presence of minute quantities of dissolved O_2 will lead to formation of PolyhHb that is not exclusively in the T-State. To synthesize 30:1 relaxed quaternary state (R-State) (oxygenated) PolyhHb, the hHb solution is fully saturated with O_2 during the polymerization reaction to yield PolyhHb molecules exclusively in the R-State. The hHb content in the reactor was maintained at 20 mg/mL in phosphate buffered saline (PBS) (0.1 M, 7.4 pH). The hHb was passed over Liquicell G453 gas membrane contactor (3M, Saint Paul, MN) via a peristaltic pump. This equipment facilitates rapid hHb gas exchange without causing bubble formation, which may lead to hHb denaturation. Pure O_2 gas was used to fully saturate the hHb solution to prepare R-State PolyhHb Pure nitrogen (N_2) gas was used to partially desaturate the hHb solution. Complete O_2 removal was obtained by addition of 333 mg of sodium dithionite ($Na_2S_2O_4$) in deoxygenated PBS 60 mL of a 0.13 to 0.18 M glutaraldehyde solution diluted in deoxygenated/oxygenated PBS (0.1 M, pH 7.4) was injected at a constant rate for 2 hours to polymerize hHb. After glutaraldehyde addition was completed, the solution was allowed to react for an additional hour before quenching with sodium cyanoborohydride ($NaCNBH_3$). After $NaCNBH_3$ was added to the reaction vessel, the solution was placed in an ice bath in a refrigerator with gentle stirring overnight.

PolyhHb was prepared using a 32-cycle two-stage continuous diafiltration process. First, 250 mL of reacted PolyhHb solution was added to the stage 1 filtration system. The solution was then filtered on a 0.2 μm polyester sulfone (PES) filter with the permeate transferred to the stage 2 filtration system. Once 250 mL of stage 1 permeate was transferred to stage 2, purification was initiated on a 100 kDa modified polyester sulfone (mPES) tangential flow filtration (TFF) module (Spectrum Labs, Rancho Dominguez, CA). PolyhHb solutions were then excipient exchanged into an isotonic modified Ringer's lactate buffer (sodium chloride (NaCl) 115 mM, potassium chloride (KCl) 4 mM, calcium chloride ($CaCl_2{\bullet}2H_2O$) 1.4 mM, sodium hydroxide (NaOH) 13 mM, sodium lactate 27 mM, and N-acetyl-L-cysteine (NALC) 12.3 mM). After 16× volume exchanges, stage 1 permeate contained insignificant concentrations of PolyhHb. At this point, stage 1 permeate was replaced with the excipient solution. After 32× volume exchanges, the permeate was measured via UV-visible spectroscopy to estimate complete removal of small hHb species. Excipient exchanged solutions were then concentrated to at least 100 mg/mL.

Quantification of biophysical properties

The cyanomethemoglobin method was used to measure the Hb concentration and the methemoglobin (metHb) level of hHb/PolyhHb solutions [5, 6]. The size distribution by volume of PolyhHb was measured using dynamic light scattering (DLS) (Brookhaven Instrument Inc. BS-200M, Holtsville, NY). O_2-hHb/PolyhHb equilibrium binding curves were measured using a Hemox Analyzer (TCS Scientific Corp., New Hope, PA). The hHb/PolyhHb rate of O_2 offloading (k_{off,O_2}) kinetics were measured with an Applied Photophysics SF-17 microvolume stopped-flow spectrophotometer (Applied Photophysics Ltd., Surrey, United Kingdom) using protocols previously described by Rameez and Palmer [7, 8]. The rheology of PolyhHb was measured using a DV3T-CP cone and plate rheometer (Brookfield AMETEK, Middleboro, MA) with cone spindle CPA-40Z as described previously [9]. The molecular weight (MW) distribution was estimated with an Acclaim SEC-1000 column (Thermo Scientific, Waltham, MA) on a Thermo Scientific Dionex Ultimate UHPLC system. The flow rate of the mobile phase (0.5 mM 7.4 phosphate buffer) was maintained at 0.35 mL/min for all runs. Full-spectrum UV-visible (200 to 700 nm) absorbance spectra were collected for the duration of each run. All quantitative measurements of PolyhHb composition were performed from the absorbance at 413 nm. To estimate the MW distribution of low MW PolyhHb, we used the manufacturer provided calibration of the SEC column corrected for holdup time in the lines.

Biophysical properties of PolyhHb

After synthesizing and purifying PolyhHb, we measured concentration, metHb level, hydrodynamic diameter, polydispersity index (*PDI*), estimated MW, fluid viscosity (μ), partial pressure of O_2 at which 50% of the hHb or PolyhHb is saturated with O_2 (P_{50}), cooperativity coefficient (n), and k_{off,O_2} of the resulting PolyhHbs that were used in this study. A summary of these properties is shown in Table A in S3 Appendix.

The protein concentration of T-State and R-State were not significantly different. However, T-State PolyhHb had significantly higher metHb level (5.8 $\pm$ 0.8 %) compared to R-State PolyhHb (4.2 $\pm$ 0.9 %). This can be explained by the duration of hHb deoxygenation before polymerization and addition of $Na_2S_2O_4$ to fully deoxygenate the hHb. For R-State PolyhHb the polymerization reaction is initiated after 1-1.5 hours of oxygenation, while in T-State it is 2 hours after deoxygentation. The extended period of time hHb was maintained at 37 ºC during the deoxygenation step may have contributed to higher metHb levels for T-State PolyhHb. Both T-State and R-State PolyhHb had significantly higher metHb levels compared to unmodified hHb (1.2 $\pm$ 0.2).

Table A. Summary of biophysical properties of hHb, 35:1 T-State PolyhHb, and 30:1 R-State PolyhHb. a: $p < 0.05$ compared to hHb; b: $p < 0.05$ compared to 35:1 T-State PolyhHb; c: $p < 0.05$ compared to 30:1 R-State PolyhHb

	hHb	35:1 T-State PolyhHb	30:1 R-State PolyhHb
Concentration (mg/mL)	-	101 $\pm$ 1.3	100 $\pm$ 3.1
metHb level (%)	1.2 $\pm$ 0.2^{bc}	5.8 $\pm$ 0.8^{ac}	4.2 $\pm$ 0.9^{ab}
Diameter (nm)	5.5 [10]^{bc}	63.7 $\pm$ 7.3^{ac}	38.0 $\pm$ 5.3^{ab}
PDI	-	0.10 $\pm$ 0.01	0.09 $\pm$ 0.01
Estimated MW (kDa)	64 $\pm$ 1^{bc}	1040 $\pm$ 110^{ac}	890 $\pm$ 140^{ab}
μ (cP)	1.9 $\pm$ 0.1^{bc}	7.5 $\pm$ 2.2^{ac}	4.7 $\pm$ 1.7^{ab}
P_{50} (mm Hg)	12.4 $\pm$ 1.3^{bc}	34.0 $\pm$ 1.1^{ac}	1.31 $\pm$ 0.07^{ab}
n	2.51 $\pm$ 0.06^{bc}	0.91 $\pm$ 0.02^{a}	0.96 $\pm$ 0.09^{a}
k_{off,O_2} (s^-1)	41.0 $\pm$ 5.12^{bc}	49.3 $\pm$ 7.1^{ac}	23.1 $\pm$ 5.1^{ab}

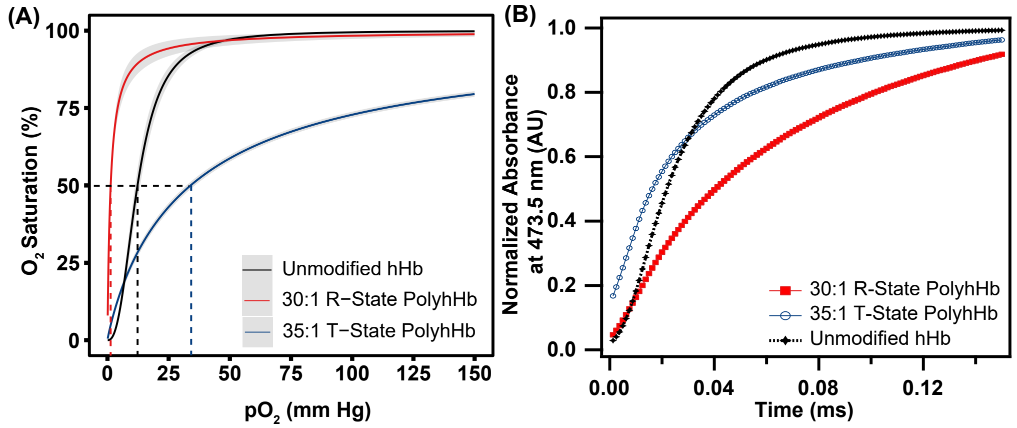

Fig A. OECs and deoxygenation kinetic time courses for PolyhHb and hHb. (A) OECs for PolyhHb and hHb (B) Comparison of time course for deoxygenation in the presence of 1.5 mg/mL sodium dithionite for hHb, 30:1 R-State PolyhHb, and T-State PolyhHb. For OECs (A) The shaded region represents the 95% confidence interval for each quaternary state with 3 runs per sample. For deoxygenation (B), the experimental data shows the average of 10-15 kinetic traces. For deoxygenation, the reactions were monitored at 437.5 nm and 20°C in 0.1 M pH 7.4 PBS. Symbols represent experimental data, and corresponding lines of the same color represent curve fits.

The O_2 equilibrium curves (OEC) for each species used in these studies are shown in Fig A in S3 Appendix. Polymerization in the T-State significantly increased the P_{50} (34.0 $\pm$ 1.1 mm Hg) compared to unmodified hHb (12.4 $\pm$ 1.3 mm Hg). In contrast, polymerization in the R-State significantly decreases the P_{50} (1.31 $\pm$ 0.07 mm Hg). Polymerization also resulted in a complete loss of sigmoidal O_2 binding as indicated by n values close to 1. These values are consistent with previous studies [3, 4, 11].

The k_{off,O_2}s for T-State PolyhHb was significantly higher than that obtained for R-State PolyhHb and unmodified hHb. Representative O_2 offlaoding curves are shown in Fig A in S3 Appendix.

Also, k_{off,O_2} values obtained for R-State PolyhHb are significantly lower than the rate constant obtained for unmodified hHb. These observations are consistent with the literature and are expected given the contrasting O_2 affinities of T-State and R-State PolyhHbs [3, 11].

Polymerization with glutaraldehyde significantly increases the diameter of both 35:1 T-State PolyhHb (63.7 $\pm$ 7.3 nm) and 30:1 R-State PolyhHb (38.0 $\pm$ 5.3 nm). Representative size distributions as measured with DLS are shown in Fig B in S3 Appendix. Both of these PolyhHbs are significantly smaller than the PolyhHb prepared and used previously [4, 9]. However, they have much lower *PDI* compared to the previous materials, which indicates more uniform polymer distributions.

The MW distribution was estimated with HPLC-SEC. The resulting chromatograms and approximate composition are shown in Fig B in S3 Appendix. As with the diameter, glutaraldehyde polymerization significantly increased the MW of the PolyhHb. However, after purification, T-State PolyhHb still contained a significant ($>5\%$) of low MW and unpolymerized hHb. Despite this, the MW of 35:1 T-State PolyhHb is still significantly higher than the MW of R-State PolyhHb.

Polymerization also increased viscosity of both the 35:1 T-State and 30:1 R-State PolyhHb. 35:1 T-State PolyhHb had significantly higher viscosity (7.5 $\pm$ 2.2 cP) compared to 30:1 R-State PolyhHb (4.7 $\pm$ 1.7 cP). This effect is likely partially correlated with the increased size of these materials. These materials were also significantly less viscous than the materials prepared previously [9].

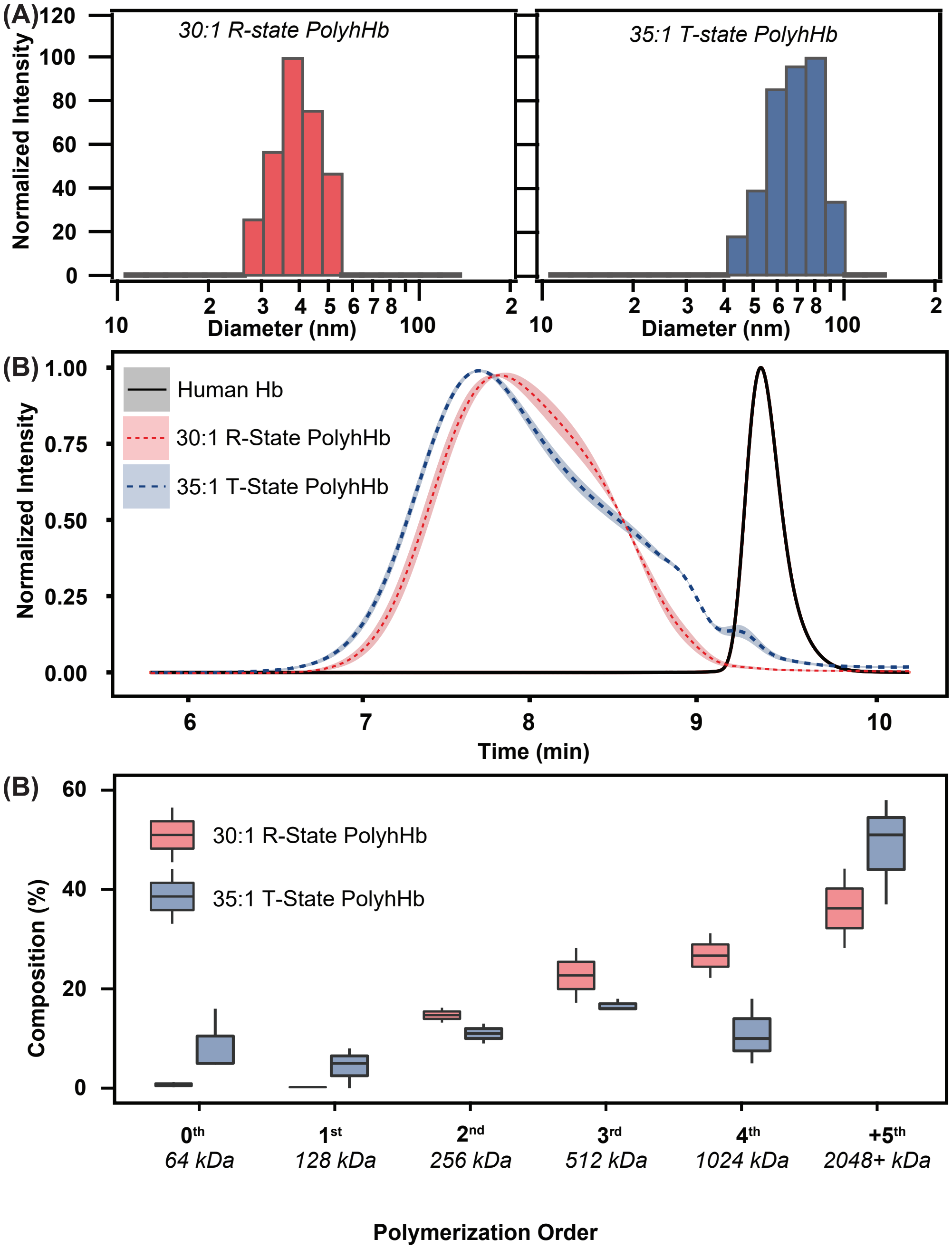

Fig B. Hydrodynamic diameter distributions, Size exclusion chromatograms, and polymer distributions of unmodified hHb, 35:1 T-State PolyhHb and 30:1 R-State PolyhHb. (A) Representative intensity distributions of the hydrodynamic diameter of 30:1 R-State and 35:1 T-State PolyhHb. (B) Normalized SEC intensity distributions of R-State and PolyhHb compared to unmodified hHb. The shaded region represents the 95% confidence interval for the average of the produced PolyhHb. (C) Polymer order distribution for 35:1 T-State and 30:1 R-State PolyhHb. Polymer distribution was calculated on a percent by heme basis via analysis of the 413 nm absorbance wavelength. The corresponding approximate sizes of the polymer orders are shown below each group.

References

1. Palmer AF, Sun G, Harris DR. Tangential flow filtration of hemoglobin. Biotechnology Progress. 2009;25(1):189–199. doi:10.1002/btpr.119.

2. Palmer AF, Sun G, Harris DR. The quaternary structure of tetrameric hemoglobin regulates the oxygen affinity of polymerized hemoglobin. Biotechnology Progress. 2009;25(6):1803–1809. doi:10.1002/btpr.265.

3. Zhang N, Jia Y, Chen G, Cabrales P, Palmer AF. Biophysical properties and oxygenation potential of high-molecular-weight glutaraldehyde-polymerized human hemoglobins maintained in the tense and relaxed quaternary states. Tissue Engineering - Part A. 2011;17(7-8):927–940. doi:10.1089/ten.tea.2010.0353.

4. Belcher DA, Banerjee U, Baehr CM, Richardson KE, Cabrales P, Berthiaume F, et al. Mixtures of tense and relaxed state polymerized human hemoglobin regulate oxygen affinity and tissue construct oxygenation. PLoS ONE. 2017;12(10):e0185988. doi:10.1371/journal.pone.0185988.

5. Hawk PB. Blood analysis. Hawk's Physiological Chemistry 14th New York: McGraw-Hill. 1965; p. 1090–9.

6. Arifin DR, Palmer AF. Determination of Size Distribution and Encapsulation Efficiency of Liposome-Encapsulated Hemoglobin Blood Substitutes Using Asymmetric Flow Field-Flow Fractionation Coupled with Multi-Angle Static Light Scattering. Biotechnology Progress. 2003;19(6):1798–1811. doi:10.1021/bp034120x.

7. Rameez S, Guzman N, Banerjee U, Fontes J, Paulaitis ME, Palmer AF, et al. Encapsulation of hemoglobin inside liposomes surface conjugated with poly(ethylene glycol) attenuates their reactions with gaseous ligands and regulates nitric oxide dependent vasodilation. Biotechnology Progress. 2012;28(3):636–645. doi:10.1002/btpr.1532.

8. Rameez S, Palmer AF. Simple method for preparing poly(ethylene glycol)-surface-conjugated liposome-encapsulated hemoglobins: Physicochemical properties, long-term storage stability, and their reactions with O2, CO, and NO. Langmuir. 2011;27(14):8829–8840. doi:10.1021/la201246m.

9. Belcher DA, Ju JA, Baek JH, Yalamanoglu A, Buehler PW, Gilkes DM, et al. The quaternary state of polymerized human hemoglobin regulates oxygenation of breast cancer solid tumors: A theoretical and experimental study. PLoS ONE. 2018;13(2). doi:10.1371/journal.pone.0191275.

10. Xu HX, Bjerneld EJ, Käll M, Börjesson L, Kall M, Borjesson L. Spectroscopy of Single Hemoglobin Molecules by Surface Enhanced Raman Scattering. Physical Review Letters. 1999;83(21):4357–4360. doi:10.1103/PhysRevLett.83.4357.

11. Buehler PW, Zhou Y, Cabrales P, Jia Y, Sun G, Harris DR, et al. Synthesis, biophysical properties and pharmacokinetics of ultrahigh molecular weight tense and relaxed state polymerized bovine hemoglobins. Biomaterials. 2010;31(13):3723–3735. doi:10.1016/j.biomaterials.2010.01.072.
