# Supplementary material for: Tumor vascular status controls oxygen delivery facilitated by infused polymerized hemoglobins with varying oxygen affinity
Source: PLoS Comput Biol. 2020 Aug 20;16(8):e1008157. doi: 10.1371/journal.pcbi.1008157 (PMC7462268; doi:10.1371/journal.pcbi.1008157)
Supplement: S4 Appendix — This file documents the comparison between the oxygenation and blood flow observed between the simulated human and host tissue in which the artificial tumor constructs were produced. (PDF) [file pcbi.1008157.s004.pdf]

S4. Appendix. Comparison of mouse and human host tissue oxygenation and blood flow.

Donald A. Belcher[1], Alfredo Lucas[2], Pedro Cabrales[2], Andre F. Palmer[1],

1 William G. Lowrie Department of Chemical and Biomolecular Engineering, The Ohio State University, Columbus, Ohio, USA
2 Department of Bioengineering, University of California, San Diego, La Jolla, CA, USA

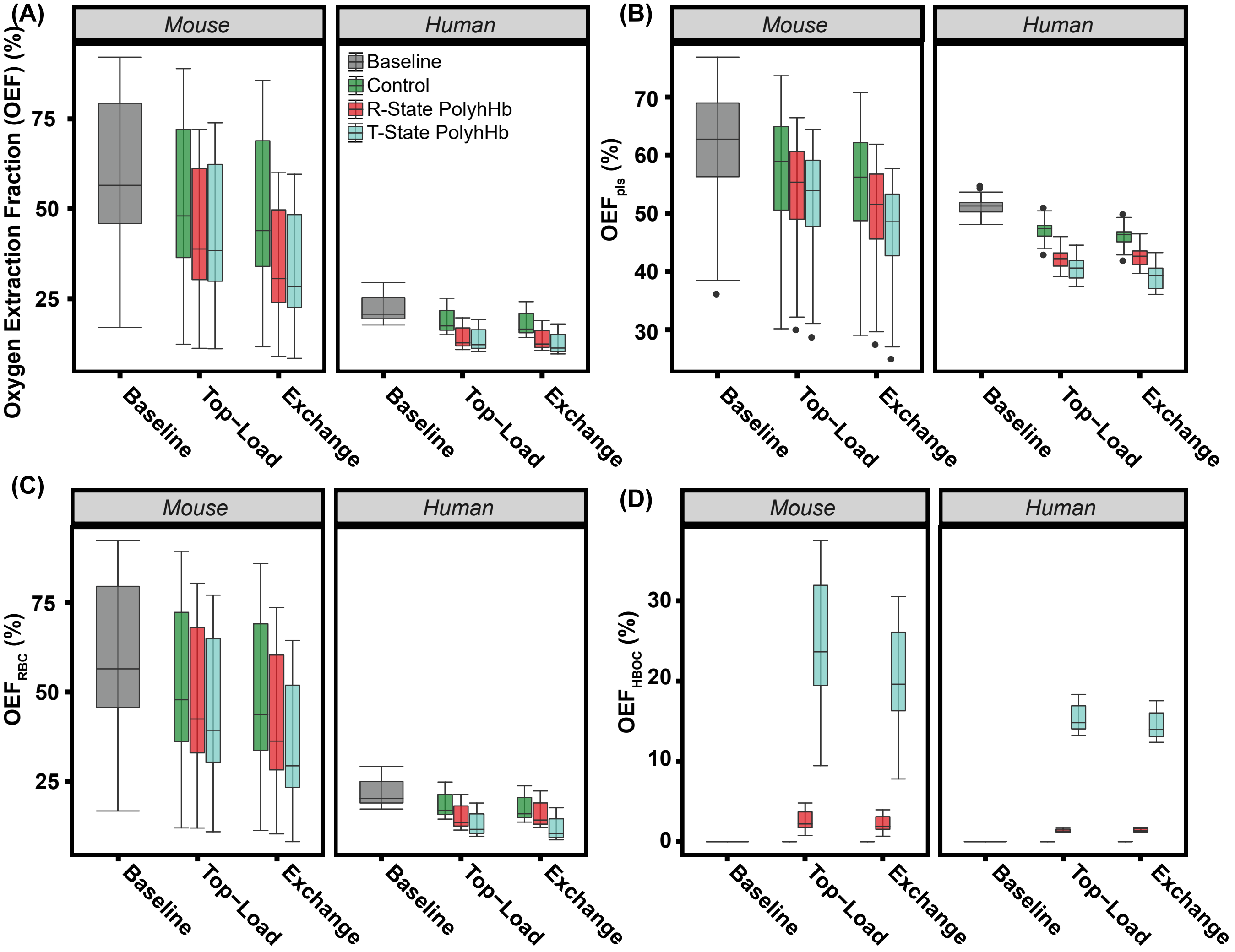

Fig A. Changes in oxygen (O_2) extraction fraction (OEF) for the simulated polymerized human hemoglobin (hHb) (PolyhHb) enhanced transfusion model in host tissue. Variations in (A) OEF, (B) O_2 extraction fraction from plasma (OEF_{plas}), (C) O_2 extraction fraction from hemoglobin (Hb) in red blood cells (RBCs) (OEF_{Hb}) O_2 saturation in RBC, and (D) O_2 extraction fraction from Hb-based O_2 carriers (HBOCs) (OEF_{HBOC}) (HBOC) concentration transfusion of the control, 30:1 relaxed quaternary state (R-State) PolyhHb, and the tense quaternary state (T-State) PolyhHb.

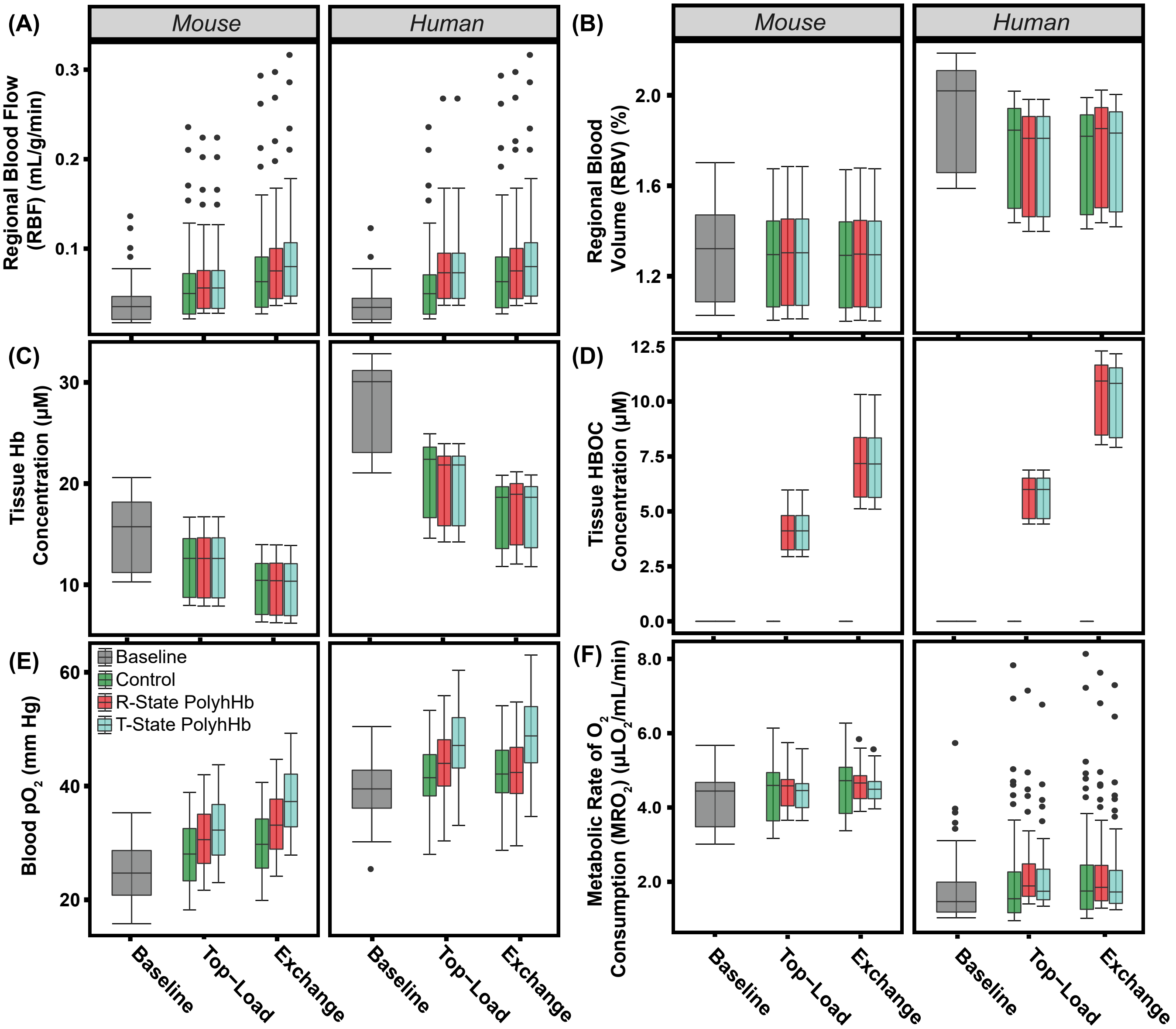

Fig B. Changes in the tumor vascular architecture and Hb/PolyhHb concentrations for the simulated PolyhHb enhanced transfusion model in host tissue. Variations in host tissue (A) regional blood flow (*RBF*), (B) regional blood volume (*RBV*), (C) Hb concentration in the tissue ($C_{Hb,tis}$), (D) PolyhHb (HBOC) concentration, (E) blood partial pressure of dissolved O_2 (pO_2), and (F) metabolic rate of O_2 consumption (MRO_2) for the baseline, top-load, and exchange transfusion of the control, 30:1 R-State PolyhHb, and the T-State PolyhHb.
