# Supplementary material for: Tumor vascular status controls oxygen delivery facilitated by infused polymerized hemoglobins with varying oxygen affinity
Source: PLoS Comput Biol. 2020 Aug 20;16(8):e1008157. doi: 10.1371/journal.pcbi.1008157 (PMC7462268; doi:10.1371/journal.pcbi.1008157)
Supplement: S5 Appendix — This file documents the method to determine the HBOC modified Sherwood number as estimated with linear regression on results from parametric sweeps on a KTC model. (PDF) [file pcbi.1008157.s005.pdf]

S5. Appendix. Determination of an oxygen transport Sherwood number

Donald A. Belcher[1], Alfredo Lucas[2], Pedro Cabrales[2], Andre F. Palmer[1],

1 William G. Lowrie Department of Chemical and Biomolecular Engineering, The Ohio State University, Columbus, Ohio, USA
2 Department of Bioengineering, University of California, San Diego, La Jolla, CA, USA

The oxygen (O_2) transport Sherwood number (Sh_{O_2}), sometimes referred to as the O_2 mass transport Nusselt number, represents the ratio of the convective mass transfer to the rate of diffusive mass transport. This dimensionless number is especially useful for calculating the transvascular mass transport coefficient that has been described in various experimental studies [1,2]. The equation to calculate the Sh_{O_2} is shown in Eq 1

$$Sh_{O_2} = \frac{2r_{ves}J_w}{(C_{O_2,tissue} - C_{O_2,plasma}) \cdot D_{O2,plasma}} \tag{1}$$

To estimate how PolyhHb modulates Sh_{O_2} in a single vascular segment we prepared a finite element model in COMSOL multiphysics as previously described [3]. To evaluate how individual properties of a vessel can affect Sh_{O_2} after various doses, we performed a parametric sweep over parameters that led to changes in overall O_2 transfer rate (k_0) determined during sensitivity analysis. The values for this sweep are shown in Table A in S5 Appendix S5. After the sweep was performed, the resulting values were numerically evaluated with nonlinear least squares
regression to determine a correlation between varied parameters and Sh_{O_2}. The correlation was selected to match the correlation empirically determined by Welter *et al.* [4]. Specifically, we sought to determine the fitting parameters baseline Sh_{O_2} parameter (p_1), offset Sh_{O_2} parameter (p_2), and gradient Sh_{O_2} parameter (p_3) shown in Eq 2.

$$Sh_{O_2} = p_1\left(1 - p_2 e^{\left(\frac{r_{ves}}{p_1}\right)}\right) \tag{2}$$

Table A. Varied parameters for determination of the Sh_{O_2} correlation.

Parameter	Values	Unit
transfusion volume equal to a set percentage of the blood volume ($TL\%$)	0, 10, 20, 30, 40	%
fluid velocity ($\bar{v}$)	0.01, 0.1, 1	cm/s
blood vessel radius (r_{ves})	3, 5, 7, 13, 19, 25, 31, 38, 44, 50	µm
hematocrit (HCT)	0.0, 0.1, 0.2, 0.3, 0.4, 0.5	
inlet partial pressure of dissolved O_2 (pO_2) of the vessel ($pO_{2,in}$)	1, 5, 10, 20, 30, 40, 50, 60, 70, 80	mm Hg

From our previous work with a modified Krogh tissue cylinder (KTC) model, we know that the polymerized human hemoglobin (hHb) (PolyhHb) enhanced O_2 flux varies with pO_2 of the vascular segment [3]. This is in contrast to unsupplemented blood vessels wherein in the O_2 flux is relatively constant as a function of the vessel pO_2. Therefore, the previous definition of the Sh_{O_2}, also referred to as the O_2 mass transport Nusselt number, defined by Welter *et. al* does not apply for PolyhHb transfusion [4].
Before determining the functions that describe each of the Sh_{O_2} parameters, we performed a sensitivity analysis using a false detection rate (FDR) test on a parameteric sweep of the KTC data. This allowed us to determine what variables should be examined for use in a Sh_{O_2}

correlation. During the estimation of the overall Sh_{O_2} we found that r_{ves}, HCT, $pO_{2,in}$, and the top-load $TL\%$ each had a substantial effect on the Sh_{O_2} (FDR greater than 0.1). Despite having a substantial impact on k_0, the $\bar{v}$ had a minor effect on Sh_{O_2} compared to the other factors (FDR less than 0.1). We then examined how each of the varied parameters affected each of the fitted Sh_{O_2} parameters (p_1,p_2, p_3). Beginning with p_2, we found that this value was independent of each variable (FDR less than 0.1). Nonlinear regression was used to determine that p_2 was approximately equal to 0.75 ± 0.01. Continuing with the examination of p_1, we found that r_{ves}, HCT, and $pO_{2,in}$ each had a significant effect (FDR greater than 0.1). For the case where there was no O_2 binding species in solution (ie. only dissolved O_2 in the plasma), p_1 was approximately equal to 4.6. When O_2 carriers were present in solution, we found that p_1 increased by a value proportional to the amount of O_2 carrier and an equation in the form of either $p_{O_2,in}^{-3} + p_{O_2,in}^{-2}$ for unsupplemented vessels or $p_{O_2,in}^{-2} + p_{O_2,in}^{-1}$ after PolyhHb transfusion. Given this information, we propose that the first derivative of the equilibrium saturation (Y) with respect to the pO_2 (Equation 3) appears to be appropriate to describe p_1. Applying nonlinear regression to the full simulated data set we found that Equation 4 can be used to calculate p_1. This indicates that O_2 offloading is likely a function of the total O_2 available from the plasma, red blood cells (RBCs), and hemoglobin (Hb)-based O_2 carriers (HBOCs) at a given pO_2. This O_2 dependence may explain the controversy in the literature regarding changes in the Sh_{O_2} in the different systems [5,6]. We also anticipate that the coefficients that adjust the first derivative of Y with respect ot the vessel pO_2 are likely related to the total O_2 available from O_2 carriers when fully saturated divided by the solubility of O_2 in plasma.

$$\frac{dY}{dpO_2} = \frac{n \cdot P_{50}^n \cdot pO_2^{n-1}}{(P_{50}^n + pO_2^n)^2} \tag{3}$$

$$p_1 = 4.6 + 68 \cdot HCT \cdot \frac{dY_{Hb}}{dpO_2} + 279 \cdot TL\% \cdot \frac{dY_{HBOC}}{dpO_2} \tag{4}$$

Determining the value for p_3 presented an interesting challenge due to drastically varying performance under normoxic and hypoxic conditions. Through analysis of varying logarithmic and exponential equations, we determined that a combination of an inverse and logarithmic pO_2 dependent equation would approximate the behavior of p_3. Nonlinear regression analysis was used to estimate the coefficients shown in equation 5.

$$p_3 = \frac{a_1 \cdot TL\% - 30 \cdot HCT + 30}{pO_2} + (2.2 + HCT) \cdot ln(pO_2) \tag{5}$$

The calculated Sh_{O_2} as a function of r_{ves} is shown in Fig A in Appendix S5. The resulting equation allows us to estimate the average O_2 flux through the vessel wall ($\bar{J_w}$) proportional to the gradient between the inter- and intra-vascular pO_2. In general, we observed asymptotic behavior as r_{ves} increases past 50 µm. At these large vessel sizes, the O_2 gradient is likely negligible compared to r_{ves}. Because of this, we treat these large vessels as large reservoirs of blood where $\bar{J_w}$ is primarily a function of the surface area as described previously [4]. As the vessel size decreases, we observe a downward trend that is similar to the values observed experimentally [1,2]. For the
unsupplemented case, we observe that a local maximum Sh_{O_2} is observed around the partial pressure of O_2 at which 50% of the hHb or PolyhHb is saturated with O_2 (P_{50}) for hHb in human RBC (P_{50} = 24 mm Hg). When supplementing with tense quaternary state (T-State) and relaxed quaternary state (R-State) PolyhHb, we observed a continuous increase in Sh_{O_2} at decreasing $pO_{2,in}$. For T-State PolyhHb, we observed that this increase began to taper off at extremely low $pO_{2,in}$s (< 1 mm Hg) for small vessels (< 50 µm). This effect indicates that the O_2 delivering capacity of T-State PolyhHb is completely exhausted by the tumor tissue under hypoxic conditions.
In contrast, R-State PolyhHb has a massive increase in Sh_{O_2} when under these highly hypoxic conditions. This increased offloading likely results from the high O_2 affinity of R-State PolyhHb. At 1 mm Hg, R-State PolyhHb still retains approximately 35% of its maximum O_2

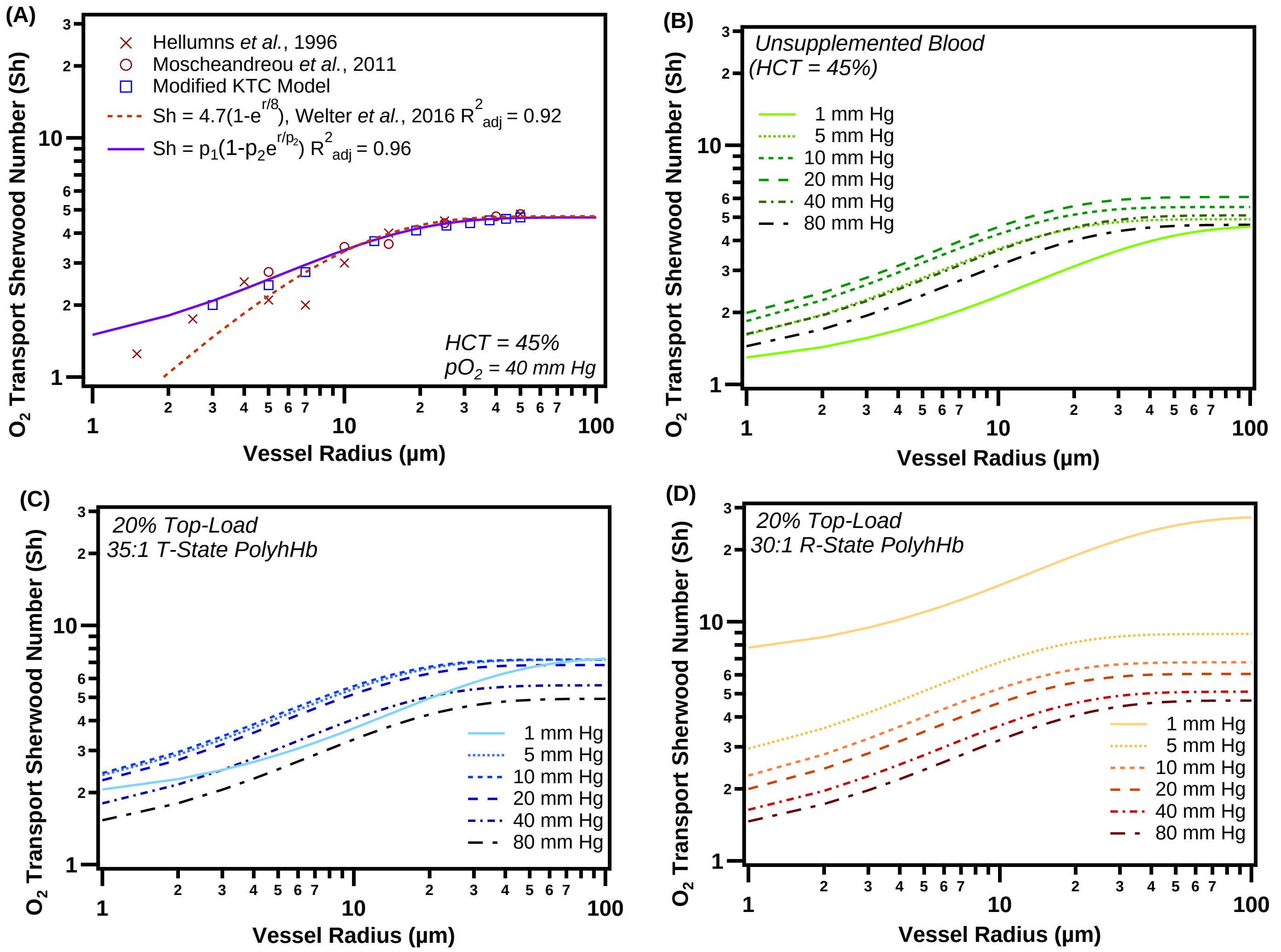

Fig A. Sh_{O_2} correlations as a function of r_{ves} and $pO2_{,in}$. (A) Sh_{O_2} comparisons with experimental and simulated studies by Hellums *et al.* [1], Moschaendreou *et al.* [2], Welter *et al.* [4], and this study. For the simulated data the $pO2_{,in}$ was set to 40 mm Hg. Also shown is Sh_{O_2} as a function of r_{ves} and $pO2_{,in}$ for (B) unsupplemented blood (C) 20% top-load with 35:1 T-State PolyhHb, and (D) 20% top-load with 35:1 R-State PolyhHb. For each of these simulated parameters $HCT = 0.45$.

carrying capacity. Under these hypoxic conditions, T-State PolyhHb only retains 2.8% of its maximum O_2 carrying capacity.

Additionally, the resulting non-linear correlation had an adjusted R^2 of 0.96 compared to data from the modified KTC model. When comparing to previous experimental and simulated data [1,2], the newly developed Sh_{O_2} correlation had a higher adjusted R^2 (0.96) compared to the model used by Welter *et al.* (adjusted R^2 (0.92)) [4]. This indicates to us that the newly developed model for the Sh_{O_2} better describes the performance of unsupplemented blood compared to the previously developed model.

Unfortunately, the experiments needed to validate the hypoxic range of the new Sh_{O_2} model are susceptible to significant noise due to low O_2 readings. Instead, we evaluated the performance of this model with O_2 distribution data obtained via intravital microscopy on tumors grown within a chamber window model.

References

1. Hellums JD, Nair PK, Huang NS, Ohshima N. Simulation of intraluminal gas transport processes in the microcirculation. Annals of Biomedical Engineering. 1995;24(S1):1–24. doi:10.1007/BF02770991.

2. Moschandreou TE, Ellis CG, Goldman D. Influence of tissue metabolism and capillary oxygen supply on arteriolar oxygen transport: A computational model. Mathematical Biosciences. 2011;232(1):1–10. doi:10.1016/J.MBS.2011.03.010.

3. Belcher DA, Ju JA, Baek JH, Yalamanoglu A, Buehler PW, Gilkes DM, et al. The quaternary state of polymerized human hemoglobin regulates oxygenation of breast cancer solid tumors: A theoretical and experimental study. PLoS ONE. 2018;13(2). doi:10.1371/journal.pone.0191275.

4. Welter M, Fredrich T, Rinneberg H, Rieger H, Hoff DV, Hingorani S. Computational Model for Tumor Oxygenation Applied to Clinical Data on Breast Tumor Hemoglobin Concentrations Suggests Vascular Dilatation and Compression. PLOS ONE. 2016;11(8):e0161267. doi:10.1371/journal.pone.0161267.

5. Vadapalli A, Pittman RN, Popel AS. Estimating oxygen transport resistance of the microvascular wall. American Journal of Physiology-Heart and Circulatory Physiology. 2000;279(2):H657–H671. doi:10.1152/ajpheart.2000.279.2.H657.

6. Tsai AG, Friesenecker B, Mazzoni MC, Kerger H, Buerk DG, Johnson PC, et al. Microvascular and tissue oxygen gradients in the rat mesentery. Proceedings of the National Academy of Sciences. 1998;95(12):6590–6595. doi:10.1073/pnas.95.12.6590.
