# Supplementary material for: Tumor vascular status controls oxygen delivery facilitated by infused polymerized hemoglobins with varying oxygen affinity
Source: PLoS Comput Biol. 2020 Aug 20;16(8):e1008157. doi: 10.1371/journal.pcbi.1008157 (PMC7462268; doi:10.1371/journal.pcbi.1008157)
Supplement: S6 Appendix — This file documents an intravital microscopy and tumor growth experimental study that was used to validate the HBOC modified O2 transport model and vascularized tumor system used in this study. (PDF) [file pcbi.1008157.s006.pdf]

S6. Appendix. Computational model validation with animal experiments

Donald A. Belcher[1], Alfredo Lucas[2], Pedro Cabrales[2], Andre F. Palmer[1],

1 William G. Lowrie Department of Chemical and Biomolecular Engineering, The Ohio State University, Columbus, Ohio, USA
2 Department of Bioengineering, University of California, San Diego, La Jolla, CA, USA

Dorsal chamber window model

Adult female 8- to 10-week old female BALB/c-nu/nu mice were used for the xenografted tumors according to protocols approved by the University of California San Diego Animal Care and Use Committee. Mice were instrumented with dorsal chamber window models as described previously [1, 2]. Human melanomas (FME and LOX) were initiated by implanting a 200-500 μm xenograft into the fascial side of the intact skin layer of the chamber window model. After implantation, tumors were allowed to grow for 7 days before analysis. Mice were divided into two groups based on the implanted tumor cell lines (FME or LOX). Each of these groups was further subdivided into three cohorts: (1) an unsupplemented baseline, (2) transfusion of 30:1 relaxed quaternary state (R-State) polymerized human hemoglobin (hHb) (PolyhHb) and (3) transfusion of 35:1 tense quaternary state (T-State) PolyhHb. At this point, mice underwent a 20% top load (20% of the mouse blood volume calculated by weight) transfusion of a 30:1 R-State PolyhHb or a 35:1 T-State PolyhHb concentrated to 100 mg/mL. PolyhHb was infused via tail vein injection. After the transfusion, the animal was placed into a restraining tube. Once in the tube, the protruding chamber window was fixed to a microscopic stage of a BX51WI intravital microscope (Olympus, New Hyde Park, NY). Tissue images were then projected to a 4815 charge coupled device camera (Cohu Industries, Poway, CA). A LUMPFL-WIR $\times$40 numerical 0.8 apeture water immersion objective (Olympus, New Hyde Park, NY) was used to carry out the measurements. Phosphorescence quenching microscopy (PQM) was used to analyze the oxygen (O_2) distribution in the tissue and vascular space as described previously [3]. This high-resolution method allows us to resolve the partial pressure of dissolved O_2 (pO_2) of arterioles and venules within the growing tumor. To determine the pO_2 in this method, we measure the decay rate of the excited palladium-mesotetra-(4-carboxyphenyl)porphyrin (Frontier Scientific Porphyrin Products, Logan, UT) bound to albumin. To observe changes in the arteriole and venule diameter, we used a video image shearing method to determine the diameters [4].

Computational oxygen transport model validation with intravital microscopy

Before we proceeded to examine tumors generated with the continuum model, we sought to validate the numerical model of O_2 transport with additional vascular O_2 distribution data gathered via intravital microscopy from a mouse chamber window model. To perform this validation, we generated an approximately two-dimensional tumor (15 mm $\times$ 15 mm $\times$ 0.05 mm) within a type E vascular network. Tumor growth was simulated for seven days with an initial radius of 0.5 mm. Before performing the PolyhHb enhanced O_2 simulation, we first adjusted the shear stress stimuli constant until we observed a similar change in flow for each PolyhHb species at the same range of vessel sizes. The resulting flow rates are shown in Fig B in S6 Appendix. A comparison of the artificially generated FME tumor and experimental tumors is shown in Fig A in S6 Appendix.
After this, we simulated PolyhHb enhanced O_2 transport with vascular adaptation. We then analyzed vessel pO_2 and hemoglobin (Hb) saturation between the *in vivo* and *in silico* data. This

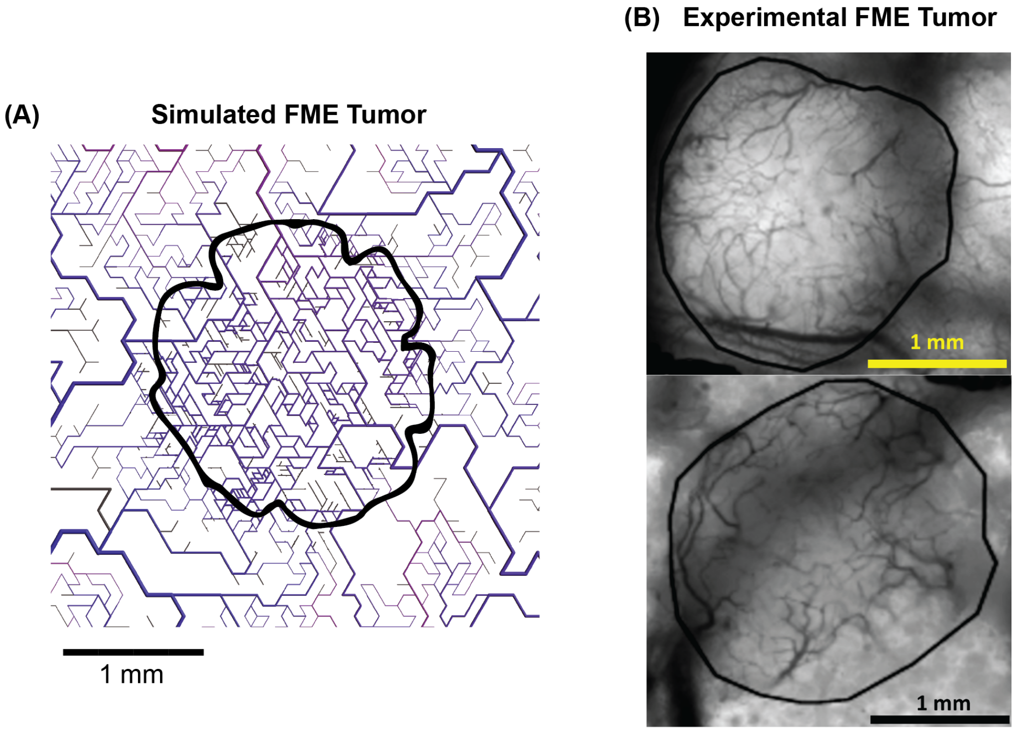

Fig A. Comparison of the experimental and simulated FME tumors. (A) Artificially generated tumor using the tumorcode simulation framework. (B) experimental FME tumor observed within the chamber window model. Black lines outline the tumor region in the images.

comparison can be found in Fig B in S6 Appenidix. Additionally, we analyzed saturation of T-State and R-State PolyhHb. The results of this analysis is shown in Fig B in S6 Appendix. There is a general agreement between the *in vivo* and *in silico* data for each of the experimental parameters. With the simulated tumor we were able to analyze every vessel within the tumor space from the arteries (blood vessel radius (r_{ves}) > 50 µm) to the capillaries ($r_{ves} = 2.5$ µm). However, in the experimental study we were only able to accurately measure the vascular pO_2 for arterioles and venules. Because of this, we isolated our comparisons to the arteriole and venule domain (25.0 -12.5 µm). Even with this restriction, the artificial vessels had a smaller average size than the experimental data. Because of this, we expect some variation in our comparisons.

In general, experimental and simulated data have similar trends for each measured parameter. The average flow rates for each vessel type is comparable; however, the experimental data has a small number of vessels, with around three times the average flow rate. This is likely because our simulation accounts for more vessels than the experimental chamber window model analysis. This broader set of simulated vessel data include larger arterioles proximal to arteries. Continuing with vascular pO_2, most vessels have an approximately normal distribution. The only exception is found during transfusion of 30:1 R-State PolyhHb to the hypoxic (FME) tumor. Under these conditions, the simulated pO_2 distribution curves are shifted to lower pO_2s as a result of severe hypoxia in the FME tumor. This trend is also found in the saturation of Hb in red blood cells (RBCs). Furthermore, the saturation of Hb in RBCs is broader in the arterioles. In the simulated normoxic (LOX) tumor arterioles, this broadened saturation distribution leads to an average that is much lower than the experimental data. The saturation of PolyhHb in the artificial tumor matches the experimental data under most conditions. However, 30:1 R-State PolyhHb is significantly less saturated in the simulation compared to the experimental conditions. The abnormal saturation of Hb in the normoxic arterioles and the PolyhHb in the hypoxic venules likely result from the simulation containing more small vessels than the

experimental data set.
One concerning feature of this data set is the increase in hypoxia after transfusion of 30:1 R-State PolyhHb. This is in direct contrast with the *in vivo* performance observed in a previous mouse model of tiple-negative breast cancer [5]. In those experimental models, tumors were less hypoxic after treatment with PolyhHb. If the shear adaptation constant was unaltered, no hypoxia formation was observed in the simulation for 30:1 R-State PolyhHb enhanced oxygenation. However, to match with the current *in vivo* data, we have kept the decrease in the shear adaptation constant throughout all remaining computational models.

Tumor growth model

Adult female 8- to 10-week old female BALB/c-nu/nu mice were used for the xenografted tumors according to protocols approved by the University of California San Diego Animal Care and Use Committee. Approximately 4×10^5 cells of the human melanoma cell lines FME and LOX were injected into the mouse flank. Mice were divided upon the tumor cell lines (FME or LOX). Tumors were allowed to grow for two weeks. After two weeks fluorescently labeled microspheres were used to estimate tumor regional blood flow (RBF) in tumors as described previously [6]. Harvested tumors were fixed in PBS 4% paraformaldehyde, embedded in paraffin cases, cut into sections, and stained. Microvascular density (MVD) was assessed by counting the number of capillary profiles within a 0.8 mm square field of view. Positive capillaries were only counted if a lumen and a brown staining endothelial cell were identified.

Comparison of averaged properties of three dimensional tumors

A comparison of the measured RBF and MVD from the experimental FME tumor in comparison with the library of generated murine tumors is shown in Fig C in S6 Appendix. The RBF measured in the experimental FME study was comparable to the Type D tumors. In addition, the MVD of the FME tumors were similar to Type A, D, E, F, G, and H tumors. Given these similarities, we think that these FME tumors would be best characterized as growing within Type D vascular beds. This is consistent with the increase in vascularization and fluid flow anticipated within the highly oxygenated flank tissue of mice.

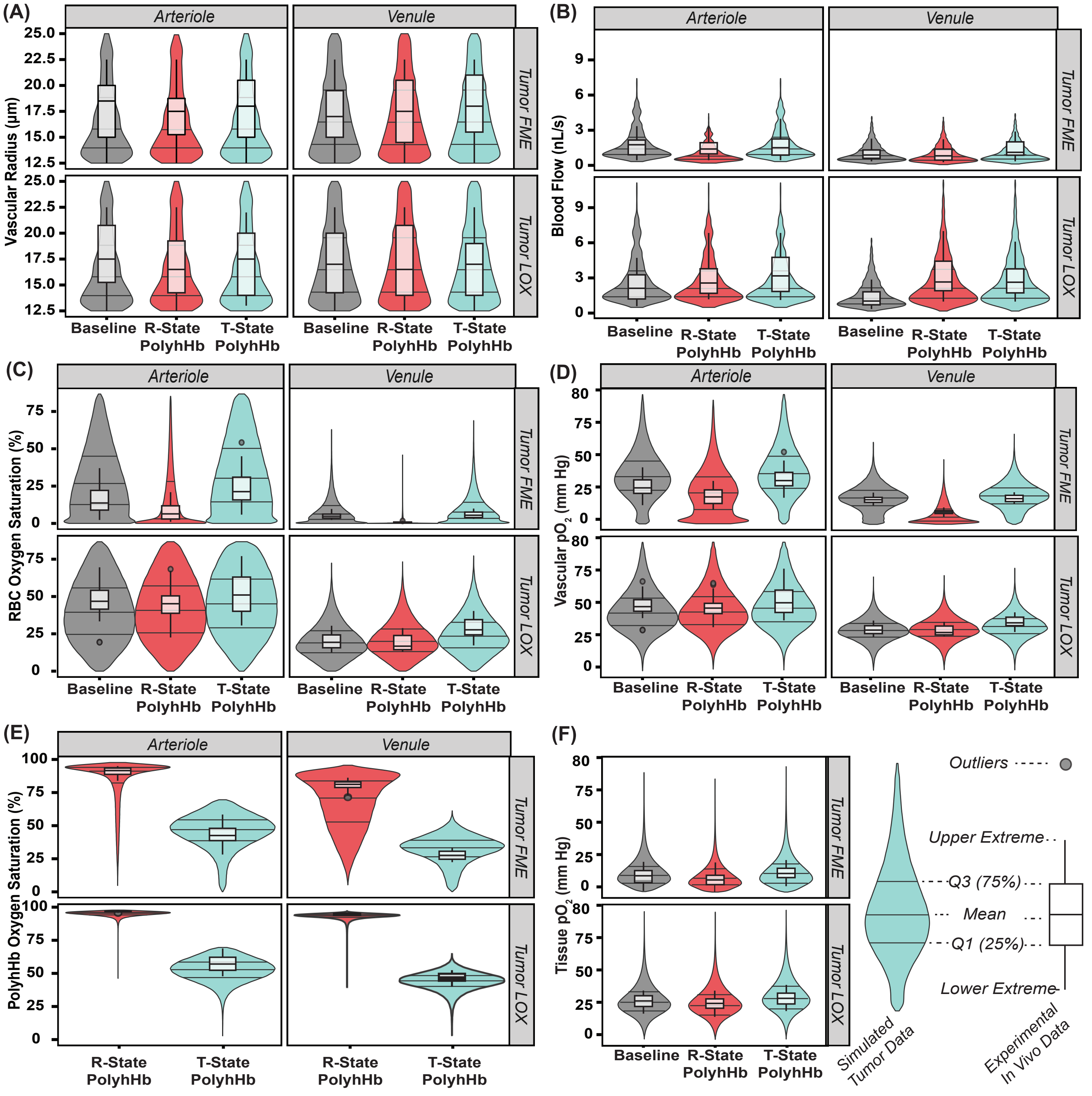

Fig B. Comparison of the experimental and simulated tumor properties in hypoxic and normoxic tissue. In this figure data is shown as for the arteriole and venule side (A) vascular radii and (B) microvascular flow data, (C) RBC Hb saturation, (D) vascular pO_2, and PolyhHb saturation in hypoxic (FME) and normoxic (LOX) tumor xenografts. Additionally, This figure shows the tissue pO_2s distribution. Experimental (*in vivo*) data is depicted as box plots. Simulated (*in silico*) data is depicted as violin plots with the first quantile (25%), mean (50%) and third quantile (75%) shown. Data is shown for the baseline and with transfusion of a 30:1 R-State PolyhHb and a 35:1 T-State PolyhHb

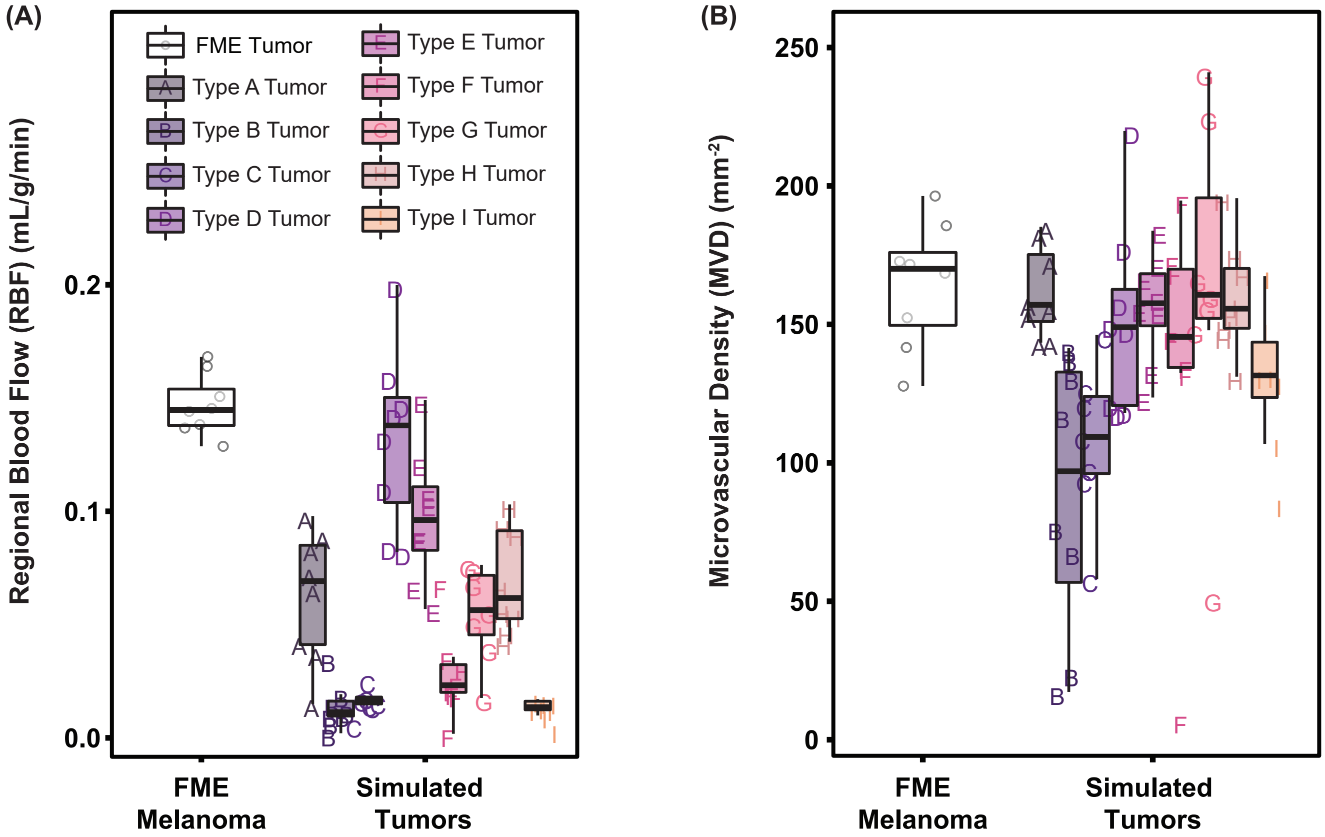

Fig C. Comparison of experimental (FME) and simulated tumor (A) regional blood flow (RBF) and (B) microvascular density (MVD).

References

1. Cabrales P, Tsai AG, Frangos JA, Intaglietta M. Role of endothelial nitric oxide in microvascular oxygen delivery and consumption. Free Radical Biology and Medicine. 2005;39(9):1229–1237. doi:10.1016/j.freeradbiomed.2005.06.019.

2. Dufu K, Yalcin O, Ao-ieong ESY, Hutchaleelala A, Xu Q, Li Z, et al. GBT1118, a potent allosteric modifier of hemoglobin O2 affinity, increases tolerance to severe hypoxia in mice. American Journal of Physiology-Heart and Circulatory Physiology. 2017;313(2):H381–H391. doi:10.1152/ajpheart.00772.2016.

3. Kerger H, Groth G, Kalenka A, Vajkoczy P, Tsai AG, Intaglietta M. pO2 measurements by phosphorescence quenching: characteristics and applications of an automated system. Microvascular Research. 2003;65(1):32–38. doi:10.1016/S0026-2862(02)00027-4.

4. Ortiz D, Briceño JC, Cabrales P. Microhemodynamic parameters quantification from intravital microscopy videos. Physiological Measurement. 2014;35(3):351–367. doi:10.1088/0967-3334/35/3/351.

5. Belcher DA, Ju JA, Baek JH, Yalamanoglu A, Buehler PW, Gilkes DM, et al. The quaternary state of polymerized human hemoglobin regulates oxygenation of breast cancer solid tumors: A theoretical and experimental study. PLoS ONE. 2018;13(2). doi:10.1371/journal.pone.0191275.

6. Wallace M, Green CR, Roberts LS, Lee YM, McCarville JL, Sanchez-Gurmaches J, et al. Enzyme promiscuity drives branched-chain fatty acid synthesis in adipose tissues. Nature Chemical Biology. 2018;14(11):1021–1031. doi:10.1038/s41589-018-0132-2.
